# Genome-wide diversity and selective pressure in the human rhinovirus

**DOI:** 10.1186/1743-422X-4-40

**Published:** 2007-05-03

**Authors:** Amy L Kistler, Dale R Webster, Silvi Rouskin, Vince Magrini, Joel J Credle, David P Schnurr, Homer A Boushey, Elaine R Mardis, Hao Li, Joseph L DeRisi

**Affiliations:** 1 Department of Microbiology and Immunology, University of California, San Francisco, California, USA; 2 Department of Biochemistry and Biophysics, University of California, San Francisco, California, USA; 3 Biological and Medical Informatics Program, University of California, San Francisco, California, USA; 4 Department of Medicine, University of California, San Francisco, California, USA; 5 Department of Genetics, Genome Sequencing Center, Washington University School of Medicine, St. Louis, Missouri, USA; 6 California Department of Health Services, Richmond, California, USA; 7 Howard Hughes Medical Institute, University of California, California, USA

## Abstract

**Background:**

The human rhinoviruses (HRV) are one of the most common and diverse respiratory pathogens of humans. Over 100 distinct HRV serotypes are known, yet only 6 genomes are available. Due to the paucity of HRV genome sequence, little is known about the genetic diversity within HRV or the forces driving this diversity. Previous comparative genome sequence analyses indicate that recombination drives diversification in multiple genera of the picornavirus family, yet it remains unclear if this holds for HRV.

**Results:**

To resolve this and gain insight into the forces driving diversification in HRV, we generated a representative set of 34 fully sequenced HRVs. Analysis of these genomes shows consistent phylogenies across the genome, conserved non-coding elements, and only limited recombination. However, spikes of genetic diversity at both the nucleotide and amino acid level are detectable within every locus of the genome. Despite this, the HRV genome as a whole is under purifying selective pressure, with islands of diversifying pressure in the VP1, VP2, and VP3 structural genes and two non-structural genes, the 3C protease and 3D polymerase. Mapping diversifying residues in these factors onto available 3-dimensional structures revealed the diversifying capsid residues partition to the external surface of the viral particle in statistically significant proximity to antigenic sites. Diversifying pressure in the pleconaril binding site is confined to a single residue known to confer drug resistance (VP1 191). In contrast, diversifying pressure in the non-structural genes is less clear, mapping both nearby and beyond characterized functional domains of these factors.

**Conclusion:**

This work provides a foundation for understanding HRV genetic diversity and insight into the underlying biology driving evolution in HRV. It expands our knowledge of the genome sequence space that HRV reference serotypes occupy and how the pattern of genetic diversity across HRV genomes differs from other picornaviruses. It also reveals evidence of diversifying selective pressure in both structural genes known to interact with the host immune system and in domains of unassigned function in the non-structural 3C and 3D genes, raising the possibility that diversification of undiscovered functions in these essential factors may influence HRV fitness and evolution.

## Background

Human rhinoviruses (HRV) are the major cause of the common cold, accounting for as much as 80% of upper respiratory infections in the fall cold season (reviewed in [1]). In the United States, the common cold is estimated to account for approximately 1 billion upper respiratory infections per year, 22 million days of missed school, and $40 billion in direct and indirect costs due to lost work and productivity [2]. Thus, despite typically presenting as a mild, self-limited upper respiratory infection, HRVs exact a significant health and economic burden on society in general. Moreover, recent evidence suggests that HRV infections may not always be mild or restricted to the upper respiratory tract. Results from *in vitro *and *in vivo *experimental studies have demonstrated that HRVs can both penetrate and damage bronchial epithelial cells in the lower respiratory tract [3-8]. HRV infections can cause acute bronchitis in healthy children and adults (especially the elderly), precipitate exacerbations in patients with asthma, chronic obstructive pulmonary disease, and cystic fibrosis, and can lead to fatal pneumonia in immunocompromised patients (reviewed in [9-12]).

Despite the ubiquity of HRV infections among healthy populations and their potentially severe clinical consequences in vulnerable populations, no preventive or curative therapies are currently available. Development of such therapies against HRV has in large part been hampered by the great diversity within the HRV genus, and the fact that multiple serotypes co-circulate during each cold season. This diversity has been traditionally characterized via a set of distinct types of phenotypic assays. Antisera neutralization studies performed in the 1960s to 1970s identified 102 distinct HRV serotypes [13]. Subsequent drug susceptibility analysis divided these 102 HRV prototype strains into two major groupings, subgroup A (HRVA), with 77 serotypes, and subgroup B (HRVB), with 25 serotypes [14]. A single serotype, HRV87, falls into neither of these two groups and is actually more similar to human enteroviruses (HEVs) than human rhinoviruses [15,16]. Identification of two cellular receptors for HRV further divided these serotypes into 2 additional groups [17,18]: the major cellular receptor (intracellular adhesion molecule 1, ICAM1) group, composed of 90 HRV serotypes [19,20], and the minor cellular receptor (low density lipoprotein receptor, LDLR) group, made up of 11 HRV serotypes [21].

More recent molecular genetic analyses of a number of subgenomic regions of HRV have largely corroborated these phenotypic classifications of the HRVs [17,22-29]. However, due to the paucity of available HRV genome sequences, it is unclear how well the diversity detected in these assays reflects the genome-wide diversity present among the characterized HRV serotypes. The genomes of only six HRV serotypes are publicly available (HRV2 [30], HRV16 [31], HRV1b [32], HRV14 [33,34], HRV89 [35], and HRV39 [36]). These genome sequences represent only a small fraction of the HRV genomic sequence space, and provide limited insight into the genome-wide diversity within this genus, or how this diversity is generated and continues to propagate from year to year.

Here, we expand this set of 6 fully sequenced HRV genomes to a more representative set of 34 genomes through whole genome shotgun sequencing of 27 diverse HRV reference serotypes and a single clinical isolate of HRV associated with an outbreak of severe lower respiratory illness in an elder care facility in Santa Cruz, CA [37]. We have used this larger and more diverse set of HRV genomes to analyze the genome-wide diversity in HRVs and to determine the selective pressure operating at each codon of the HRV genome. Mapping these selective pressure data onto available three dimensional HRV protein structures relative to known functional domains has provided insight into the underlying biology driving evolution of these HRV prototypes and serves as a springboard for future analyses of novel and currently circulating HRVs and the drugs developed to inhibit them.

## Results

### Generation of a representative set of HRV genome sequences for analysis

In order to obtain an accurate picture of the genetic diversity and selective pressure across the HRV genome, our first task was to expand the set of 6 fully sequenced HRV serotypes to a larger set of HRV genomes that more fully captured the genetic diversity of the known set of 102 serotypes. Since the capsid region has been found to be the most variable portion of other fully sequenced picornavirus genomes [38,39], we utilized previously generated capsid gene phylogenies of the 102 HRV serotypes [25,26,28] to identify an additional set of HRV serotypes that would prove most informative for our analysis. We identified 28 additional serotypes from across the HRV gene capsid phylogenies (Additional File 1, Figure S1) that yielded selective pressure results for the VP1 gene that were well-correlated with the results obtained from the full set of 102 HRV serotype VP1 gene sequences (Materials and Methods, Additional file 1, Figure S2). We thus focused our whole genome shotgun sequence analysis efforts on recovery of genome sequence from these 28 HRV serotypes. Combined with the 6 previously sequenced HRV genomes and the rhino/entero HRV87 genome, this provided a larger, more representative set of 35 HRV genomes for further analysis.

### Consistent phylogenetic pattern observed at every locus of the HRV genome

With this expanded set of HRV genomes in hand, we next examined the agreement between the HRV genomic and subgenomic phylogenies. Prior comparative sequence analysis of two other picornaviruses, the human enteroviruses (HEVs) and the Foot-and-Mouth Disease viruses (FMDVs) have uncovered significant incongruences between the genomic and subgenomic phylogenies of these viruses that suggest that recombination plays a significant role in generating diversity in the picornavirus family [38,40-42]. Comparison of the phylogenies of more extensively sequenced structural and non-structural subgenomic regions of the HRV genome have suggested that similar phylogenetic incongruences may be present in the HRV genome [25,26,28,29]. However, more recent analysis of the prior set of 5 fully sequenced HRVA genomes and a review of the subgenomic data has cast doubt on these conclusions [43].

**Figure 1:**
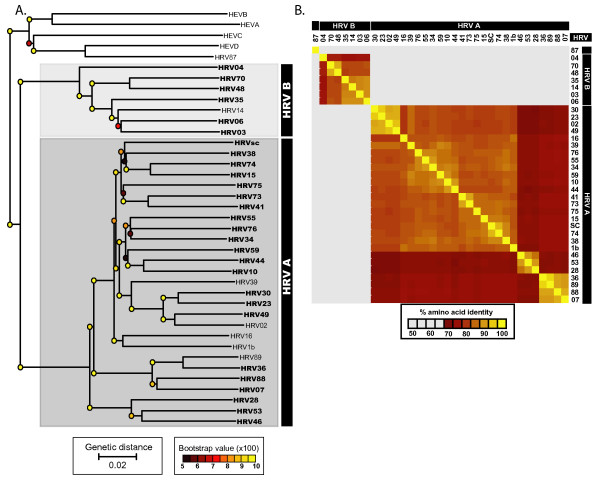
**Genetic relationship among 35 diverse HRV genomes**. A. Neighbor-joining phylogenetic tree based on whole genome nucleotide sequence of HRVs and representative HEV species. Dark gray box, HRV subgroup A genomes (27 genomes), pale gray box, HRV subgroup B genomes (7 genomes). Bold, HRV strains sequenced in this study (28 genomes); plain text, whole genome sequences for previously sequenced HRV genomes (NCBI accession numbers: HRV001b, 221708; HRV002, 61098; HRV014, 9626735; HRV016, 409463; HRV039, 53987041; HRV89, 9627730; HRV87/HEV68, 41019061) and HEV genome sequences (NCBI accession numbers: HEVA, NC_001612; HEVB, NC_001472; HEVC, NC_001428; HEVD, NC_001430). B. Whole genome pairwise amino acid identity matrix. Deduced amino acid sequences from the coding region of the 35 fully sequenced HRV genomes were compared in all possible pairwise combinations then clustered on both the X and Y-axis according to similarity in pairwise sequence identity profiles. HRV serotype is indicated by number on X and Y-axis flanking the matrix, HRVA and HRVB subgroup membership is shown in black bar above serotype identifiers.

Our analysis indicates that the whole genome phylogeny of HRV is essentially identical to the subgenomic phylogenies derived from every locus of the HRV genome, at both the nucleotide and amino acid level (Figure 1A; Additional file 1, Figure S3; Additional file 1, Data S1 and Data S2). The HRVs separated into two main branches, HRVA and HRVB, which correlated directly with their prior classification based on drug susceptibility [14]. Within each of these two major HRV genetic subgroups, the HRVs further clustered in a manner consistent with previously described cellular receptor usage [19,20] and antisera inhibition and cross-neutralization properties [13]. Consistent with its reclassification as a member of HEVD, HRV87 clustered more closely with HEVs than HRVs [28].

### Pairwise sequence analysis shows consistent diversity across the genome

**Figure 2:**
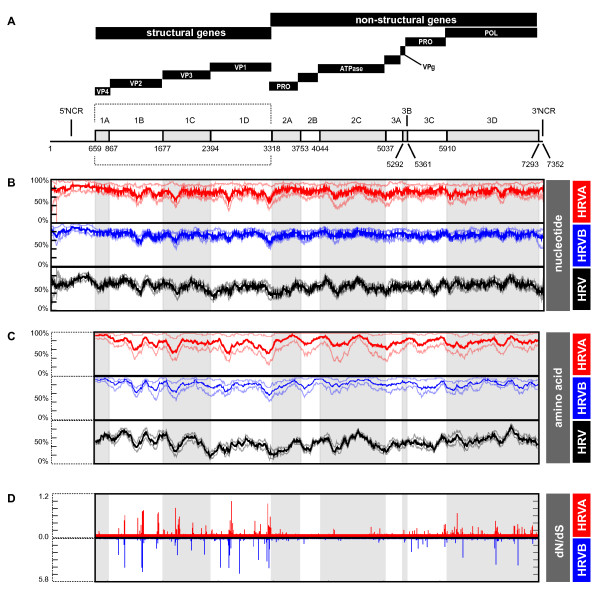
**Genetic diversity and selective pressure in the HRVA and HRVB genomes**. A. HRV genome organization. Genome schematic depicting genes in coding regions (boxes) and the non-coding regions (lines). Black bars above genome schematic indicate classes of gene products and gene product identities, where known VP = viral protein; PRO = viral protease; ATPase = DEXH-box ATPase protein; VPg = viral protein genomic (highlighted by dotted box); POL = RNA dependent RNA polymerase; NCR = non-coding region; coordinates of gene boundaries derived from alignment of available HRV genome sequences; gray shading of every other gene is provided for orientation in lower panels. B. Pairwise nucleotide identity scans within and between HRVA and HRVB genomes in a window of 100 nucleotides, advanced in single nucleotide steps across the genome. C. Pairwise amino acid identity scans within and between HRVA and HRVB genomes in a window of 50 amino acids, advanced in single amino acid steps across the genome. D. Ratio of the number of non-synonymous to synonymous mutations (dN/dS) across the genome inferred from the sequences of the HRVA (red plot) and HRVB (blue plot) genomes. Maximal dN/dS for window size of 3 codons, advanced in single codon step, are plotted. For panels B and C, bold plots, correspond to average % pairwise sequence identity values; pale plots, minimum and maximum % pairwise sequence identity values.

Average pairwise sequence analysis of both the genomic and subgenomic regions of the HRVA and HRVB genomes corroborated our phylogenetic findings (Figure 1B), revealing a consistent level of sequence identity at every locus of HRV genome (Tables 1 and 2). However, spikes of genetic diversity were detectable in multiple loci (1B, 1C, 1D, 2C, 3A, 3C, and 3D genes) at both the nucleotide (Figure 2B) and amino acid level (Figure 2C). These profiles are quite distinct from those previously observed for other picornaviral genome sequences which display high diversity in the structural genes and low diversity in the non-structural genes (Additional file 2, Figure S4 [43]). This distinct pattern of pairwise sequence identity and the lack of detectable incongruence between HRV genomic and subgenomic phylogenies raises the possibility that in contrast to other picornaviruses, recombination may not be the major driver of diversification of the HRV genome.

**Table 1:** Average % pairwise identity detected among HRV nucleotide sequences

Genome locus	HRV ave (min, max)*	HRVA ave (min, max)**	HRVB ave (min, max)^#^	HRVAvsHRVB ave (min, max)^$^
Genome	68 (56,88)	74 (69,88)	75 (71,82)	57 (56,58)
5'NCR	77 (64,95)	82 (74,95)	84 (78,91)	68 (64,71)
1A	74 (53,90)	82 (75,90)	80 (75,86)	58 (53,63)
1B	69 (59,88)	73 (66,88)	74 (69,78)	62 (59,65)
1C	66 (53,87)	71 (65,87)	73 (66,80)	56 (53,59)
1D	63 (48,87)	70 (63,87)	72 (68,78)	50 (48,53)
2A	68 (43,89)	78 (68,89)	73 (68,81)	48 (43,52)
2B	67 (46,87)	75 (63,87)	75 (66,82)	52 (46,57)
2C	67 (53,89)	72 (61,89)	76 (70,84)	55 (53,57)
3A	67 (49,91)	72 (60,91)	76 (67,86)	55 (49,61)
3B	69 (48,94)	75 (48,94)	70 (54,86)	57 (48,70)
3C	68 (53,88)	75 (68,88)	73 (69,83)	56 (53,60)
3D	69 (58,87)	73 (67,87)	74 (70,82)	60 (58,62)
3'NCR	ND	ND	ND	ND

*n = 34; **n = 27; ^#^n = 7; ^$^n = 34

**Table 2:** Average % pairwise identity detected among HRV amino acid sequences

Genome locus	HRV ave (min, max)*	HRVA ave (min, max)**	HRVB ave (min, max)^#^	HRVAvsHRVB ave (min, max)^$^
Genome	70 (50,96)	80 (70,96)	83 (76,92)	51 (50,52)
1A	82 (52,100)	96 (90,100)	94 (90,99)	56 (52,58)
1B	74 (57,96)	80 (72,96)	82 (75,89)	61 (57,64)
1C	68 (48,97)	76 (66,97)	83 (73,92)	53 (48,56)
1D	61 (37,94)	71 (61,94)	77 (71,86)	40 (37,43)
2A	71 (34,99)	88 (75,99)	81 (72,92)	37 (34,41)
2B	69 (40,99)	82 (60,99)	88 (75,98)	45 (40,49)
2C	70 (47,98)	80 (63,98)	88 (75,95)	49 (47,52)
3A	68 (41,99)	79 (63,99)	88 (74,96)	47 (41,54)
3B	79 (52,100)	89 (76,100)	83 (65,100)	60 (52,67)
3C	72 (43,97)	83 (71,97)	84 (76,96)	49 (43,53)
3D	72 (54,96)	80 (69,96)	85 (77,91)	57 (54,60)

*n = 34; **n = 27; ^#^n = 7; ^$^n = 34

### Recombination scan predicts only small, scattered events in the HRV genome

To directly compare the type and frequency of recombination events in HRV relative to other members of the picornavirus family, we performed a genome-wide scan for recombination events among the fully sequenced HRV genomes (Materials and Methods). This analysis identified ten putative recombination events (Additional file 2, Table S1). However, in contrast to the large-scale single crossover events that have been previously detected between the structural and non-structural genes of HEV and FMDV genomes [38-44], all of the events detected in the HRV genomes were small in size (average length: 281 bp, range: 84–474 bp) and predicted to result from double crossover events localized mainly in the 5'NCR of the genome and a few distinct loci scattered throughout the coding region of the genome (Additional file 2, Table S1). Thus, the extent and scope of recombination predicted to have occurred in these representative HRV genomes is indeed quite different from that seen for HEVs and FMDVs.

### Selective pressure across the human rhinovirus genome

We next investigated how HRV diversity might have arisen by analyzing the types of evolutionary forces acting on the HRV genome. We utilized the genome-based HRV phylogeny and the available genome sequences to compute the ratio of non-synonymous to synonymous changes (dN/dS) for each codon in the HRVA and HRVB genomes (Materials and Methods). Such calculations allowed us to create selective pressure profiles for the HRVA and HRVB genomes as a whole, providing an overview of the evolutionary landscape of the HRV genome (Figure 2D).

Overall, we detected similar selective pressure profiles for the HRVA and HRVB genomes (Figure 2D). Intriguingly, this selective pressure analysis reveals that a large proportion of the genome is under purifying selective pressure (82.65% for HRVA and 86.74% for HRVB), exhibiting codon-specific dN/dS ratios at the lower limits of detection (<0.06), despite the high level of genetic diversity we detected across the HRV genomes by scanning pairwise analysis. However, this purifying selective pressure is not distributed uniformly across the genome. It predominates in the central region of the genome that includes a set of non-structural genes (2A, 2B, 2C, 3A, and 3B) that interact with both viral factors and essential host cell factors during the viral replication cycle, and is also detectable across the majority of the 1A gene, which encodes the VP4 capsid protein that assembles on the interior side of the viral particle. Interrupting these regions of purifying selective pressure are two major clusters of residues with elevated dN/dS values: one in a subset of the structural genes (1B, 1C, and 1D) which lie on the outer surface of the viral capsid, and another in a pair of the non-structural genes (3C and 3D) which encode a protease and polymerase essential for viral replication.

### Structure-function mapping of diversifying residues in structural genes

To gain insight into the functional significance of these clusters of diversifying selective pressure detected within the HRV genome, we next examined how the location of the clusters of diversifying residues correlated with previously characterized functional and structural domains within the HRV genome. We first focused on the diversifying structural genes and examined the location of diversifying capsid residues relative to three previously characterized functional domains of the HRV virion: the neutralizing immunogen (NIm) sites, the cellular receptor contacts, and the binding pocket of pleconaril, a potent capsid inhibitor of HRVs and HEVs [45].

**Figure 3:**
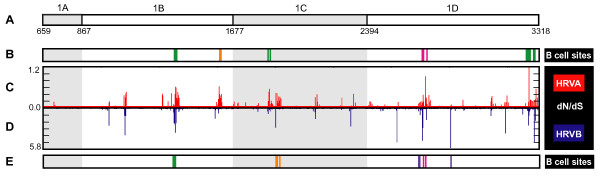
**Location of selective pressure and known immunogenic sites in capsid genes**. A. Zoom-in on capsid region of genome (boxed region from Figure 2), schematized as described in Figure 2. B. Location of HRVA antigenic sites A (magenta), B (green), and C (orange) based on studies of HRV2 (Appleyard et al., 1990; Hastings et al., 1990; Speller et al., 1993; Hewat and Blaas, 1996; Hewat et al., 1998). C, D. Zoom-in on dN/dS plot for capsid genes of HRVA and HRVB, respectively. E. Location of HRVB antigenic sites NimIA (magenta), NimIB (violet), NimII (green) and NimIII (orange) based on studies of HRV14 (Sherry and Rueckert, 1985; Sherry et al., 1986).

**Figure 4:**
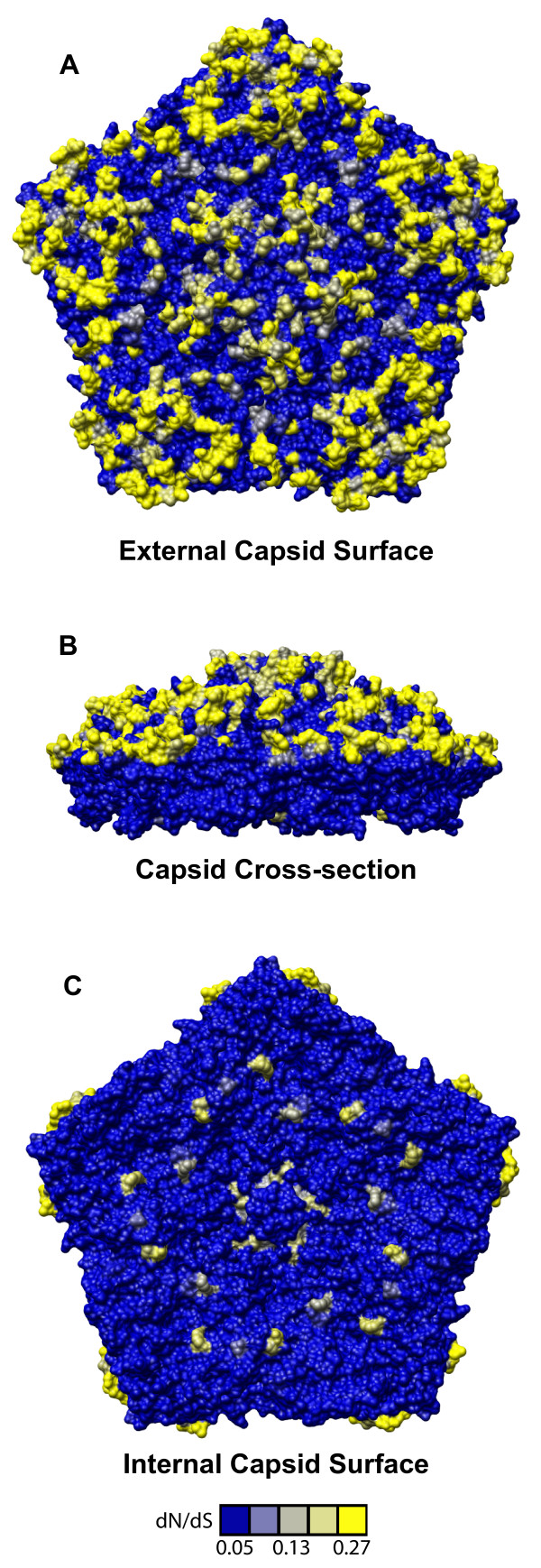
**Distribution of selective pressure on the HRV capsid pentamer subunit**. Capsid pentamer subunit from the HRV16 viral particle crystal structure (Hadfield, et al., 1997) with residues shaded in yellow according to their corresponding dN/dS values (scale bar below panel C). A. External view. B. Cross-sectional (inside/outside) view. C. Internal face.

**Figure 5:**
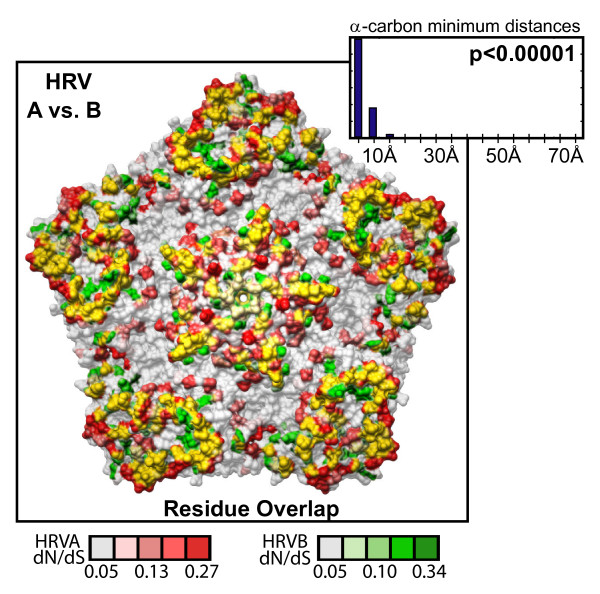
**Comparison of selective pressure in HRVA and HRVB capsid genes**. Overlay of diversifying selective pressure detected on the HRV capsid pentamer structure for HRVA (based on HRV2 capsid structure (Verdauger et al., 2000)) and HRVB (based on HRV14 capsid structure (Stanway et al., 1984)); HRVA and HRVB residues are shaded according to their corresponding dN/dS values as indicated below by the scale bar, with directly overlapping diversifying residues highlighted in yellow. Inset histogram, distribution of minimal distances between α-carbons of diversifying residues in HRV2 and HRV14; Y-axis is simple frequency count; p value provides frequency at which an average minimum distance similar to that for the observed distribution was detected when the locations of the diversifying residues were randomized on each pentamer surface, overlaid, and measured (n = 100,000 randomizations).

**Figure 6:**
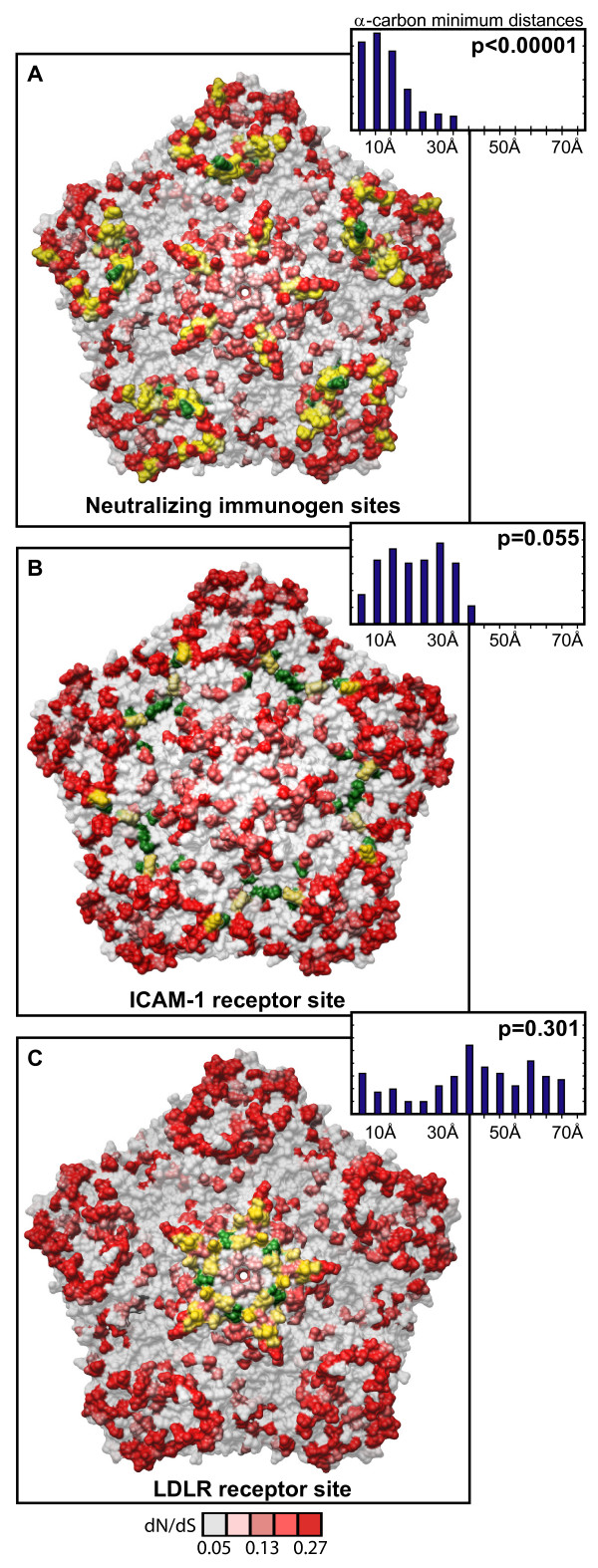
**Distribution of diversifying capsid residues relative to functional domains**. Diversifying residues in the HRV2 capsid pentamer (Verdauger et al., 2000) overlaid onto the characterized HRV antigenic sites (Appleyard et al., 1990; Hastings et al., 1990; Speller et al., 1993; Hewat and Blaas, 1996; Hewat et al., 1998). B. Diversifying residues in the HRV16 capsid pentamer (Hadfield, et al., 1997) overlaid onto the characterized ICAM1 cellular receptor contacts (Bella et al., 1999). C. Diversifying residues in the HRV2 capsid pentamer (Verdauger et al., 2000) overlaid onto the characterized LDLR cellular receptor contacts (Verdauger et al., 2004). Diversifying residues are shown in red, shaded according to corresponding dN/dS values as indicated by the scale bar below panel C; green, antigenic residues (A); ICAM1 receptor contacts (B), and LDLR contacts (C); yellow, diversifying residues that directly overlap functional residues. Inset histogram, distribution of minimal distances between α-carbons of diversifying residues and antigenic sites (A), ICAM1 contact residues (B), and LDLR contact residues (C); Y-axis is simple frequency count, with a range that varies for each panel; p values provide frequency at which an average minimum distance similar to that for the observed distribution was detected when the locations of the diversifying residues were randomized on each pentamer surface, and minimal distances to antigenic site residues (A), ICAM1R contact residues (B), and LDLR contact residues (C) were measured (n = 100,000 randomizations).

The diversifying capsid residues are distributed throughout the VP2, VP3, and VP1 capsid genes in generally overlapping positions within the HRVA and HRVB genomes (Figures 3C and 3D, respectively). Overlap can also be detected between these diversifying residues and the primary sequence location of a set of empirically determined NIm sites in HRVA (Figure 3B, [46-50]) and HRVB (Figure 3E, [51,52]). Mapping the HRVA diversifying residues onto the 3-dimensional structure of the viral pentamer subunit of the HRV particle revealed that virtually all of the diversifying capsid residues localize to protrusions or ridges on the external face of the viral particle (Figure 4). Direct comparison of the location of the diversifying capsid residues in HRVA and HRVB on the surface of the viral pentamer demonstrated significant overlap in their three-dimensional locations (p < 0.00001 Figure 5, inset histogram; Additional file 3, Figure S5, Materials and Methods). Mapping the diversifying capsid residues relative to the previously defined NIm sites (Figure 6A) and the characterized contacts for the major (ICAM1R, Figure 6B, [53]) and minor (LDLR, Figure 6C, [54]) cellular receptors for HRV also revealed detectable overlap with each of these functional domains of the HRV virion. However, quantitation of the minimum distances between the alpha carbons of the diversifying residues and the residues within each of these functional domains revealed that only the NIm sites lie within statistically significant proximity to the diversifying capsid residues (p < 0.00001; Figure 6A, inset histogram, Additional file 3, Figure S6). These results hold even if our analysis is restricted to the most diversifying capsid residues (Additional file 3, Figure S7). Thus, the distribution of the diversifying capsid residues in the structural genes are best explained by their proximity to the NIm sites, indicating that the diversification detected in the structural genes of the HRV genome may be driven in large part by pressure to evade the host humoral response.

In contrast, analysis of the selective pressure in the capsid residues within the pleconaril binding site revealed an overall paucity of diversifying selective pressure (Additional file 3, Table S2). However, one of the residues lining the pleconaril binding site in the VP1 gene (residue #191) has diversifying selective pressure detectable above background. Intriguingly, this residue corresponds to one of two residues in the binding pocket shared among naturally occurring pleconaril resistant HRVB serotypes. When mutated in a susceptible HRVB serotype, residue #191 has been shown to confer a 30-fold reduction in pleconaril susceptibility [55].

### Structure-function mapping of diversifying residues in non-structural genes

Given the essential nature of the functions performed by the products of the non-structural genes, it was quite surprising to detect a cluster of diversifying selective pressure within the 3C and 3D genes of the HRV genome. The wealth of structural and functional observations concerning these two factors allowed for analysis of the correlation in location of diversifying residues relative to the structural and functional domains previously characterized in each of these two non-structural genes.

**Figure 7:**
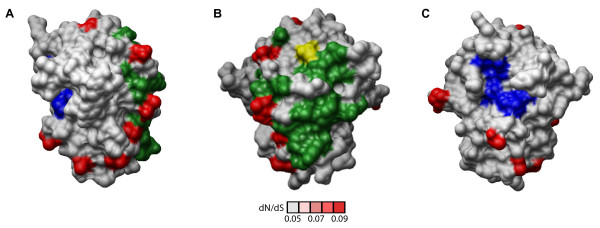
**Location of diversifying residues and functional residues in the 3C protease**. Three different views of diversifying residues in the HRV2 3C protease relative to protease active site residues (blue; (Matthews et al., 1999)) and residues implicated in RNA binding and VPg binding (green; (Matthews et al., 1999). A. Relative to both protease and RNA binding/VPg interacting domain. B. Relative to RNA binding/VPg interaction domain. C. Relative to the proteolytic active site (Matthews et al., 1999). Diversifying residues are shown in red, shaded according to their corresponding dN/dS values indicated by the scale bar; yellow, diversifying residues that directly overlap functional residues.

The diversifying residues of the 3C protein (Figure 7A) wrap around the circumference of the protein, along an axis between its RNA binding/VPg interaction domain and protease active site. None of the diversifying residues overlap with the protease active site (Figure 7C) or contacts with the characterized inhibitor, ruprintrivir ([22], Additional file 4, Table S3). However, approximately half of the diversifying residues map adjacent to the boundary of residues implicated in RNA binding/VPg interaction, with one residue directly overlapping a residue implicated in VPg binding (Figure 7B, overlapping residue in yellow). The remaining diversifying residues are present in regions of the 3C protein that are distant from both the protease active site and the RNA binding/VPg interaction domain. The close proximity of a large proportion of the diversifying residues in the 3C protein to the RNA binding/VPg primer interaction domain raises the possibility that diversification in the 3C protease may be driven in part by pressure to modulate the RNA binding or VPg binding activity during viral replication. However, given our current understanding of the 3C protein, the possible functions of the remaining diversifying sites are less clear.

**Figure 8:**
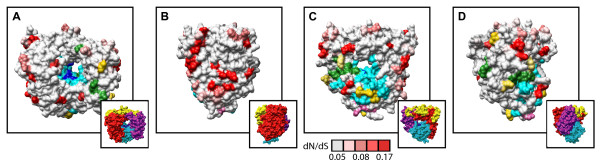
**Location of diversifying residues and functional residues in the 3D polymerase**. Front view (A), side view of fingers subdomain (B), back view (C), and side view of thumb subdomain (D) of the HRV14 3D polymerase structure (Love et al., 2004). Cyan, palm subdomain residues; blue, catalytic residues; green, residues implicated in VPg and CRE binding; pink, potential oligomerization interface I residues. Diversifying residues are shown in red, shaded according to their corresponding dN/dS values indicated by scale bar below panel C; yellow, diversifying residues that directly overlap functional residues. Insets A-D, provided for orientation to 3D polymerase subdomains: red, fingers subdomain; cyan, palm subdomain; purple, thumb subdomain; yellow, N-terminal residues.

In the 3D polymerase, a number of diversifying residues also overlap or lie in close proximity to previously described functional domains known to influence polymerization activity and catalysis. This is most obvious on the backside of the polymerase (Figure 8C). Here, a set of diversifying residues directly overlap with a domain previously implicated in coordinating movements in the polymerase that are required for catalytic activity or map nearby the binding domain for VPg, the protein primer for replication. Overlap was also detected in the thumb domain (Figures 8A and 8D), with a residue implicated in forming part of a domain analogous to the Interface I oligomerization domain of the poliovirus 3D polymerase [56].

A number of diversifying residues were also observed in regions of the 3D protein for which functional data is lacking. This is the case for a large set of diversifying residues found to localize to the outer surface of the fingers subdomain of the polymerase (Figures 8A and 8B). The role that this large domain plays in polymerase activity is not completely resolved. Recent work has demonstrated at least one residue in this domain (the highly conserved G64) can influence polymerase fidelity [57-60]. However, because this residue lies distant from the diversifying residues we detect on the surface of the fingers subdomain, their possible functional significance is unclear. Taken together, these data indicate, that like the 3C protease, proximity to characterized functional domains of the 3D polymerase does fully explain the diversifying pressure detected in this essential viral factor.

### Conservation of non-coding RNAs and essential structural elements

Like all members of *the Picornaviridae *family, HRVs possess a number of essential *cis*-acting RNA elements that are required for, or enhance viral replication [61]. An essential cloverleaf structure and internal ribosomal entry site (IRES) have been identified in the 5' non-coding region of the genome, while a small hairpin RNA element that enhances replication has been found in the 3' non-coding region. An additional essential RNA structure, a small stem-loop *cis*-acting replication element (CRE) resides within the coding sequences of the *Picornaviridae *genomes.

In our analysis of 34 HRV genome sequences, evidence for conservation of each of these elements was detected at both the primary sequence and secondary structure level (Additional file 4, Data S3 and S4). While these structures have been inferred previously from phylogenetic comparisons of available HRV genomes [61], our analysis provides a robust HRV consensus structure for each element in the 5' and 3' non-coding region (Additional file 4, Data S3 and S4).

**Figure 9:**
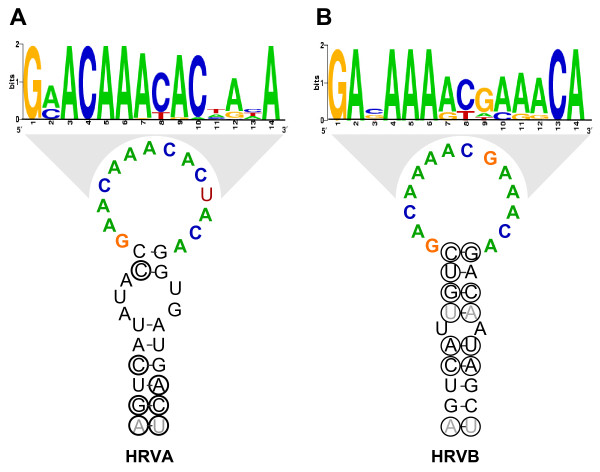
**Consensus structures and loop sequences for HRVA and HRVB minimal CREs**. A. Consensus secondary structure and sequence of HRVA minimal CRE derived from alignment of publicly available HRV prototype sequences in the region of the 2A gene (Laine et al., 2005) identified to be the minimal functional CRE in HRV2 (Gerber et al., 2001). B. Consensus secondary structure and sequence of HRVB minimal CRE derived from an alignment of sequence from all HRVB prototypes in the region of the 1D gene (Ledford et al., 2004; Laine et al., 2005) shown to function as the minimal CRE in HRV14 (McKnight and Lemon, 1998; Yang et al., 2002). Circled residues, positions where compensatory substitutions are detected in the alignment. Gray residues indicate positions where substitutions that disrupt basepairing potential are detected in the alignment. Weblogo (Schneider and Stephens, 1990; Crook et al., 2004) of consensus sequence of loop region is provided above to provide a quantitative view of the conservation of this element. The height of each letter is proportional to the fraction of the observed frequency relative to the expected frequency at each position.

Since sequence from all 102 HRV prototypes is available for regions in which the CREs have been mapped, we utilized the entire set of HRV prototypes to assess the conservation of the HRVA and HRVB CRE sequence and structure. Within the HRVA genomes, a highly conserved CRE-like sequence and structure containing a short stem with a 14 nucleotide loop conforming to the published CRE loop consensus, R^1^NNNAAR^2^NNNNNR^3 ^[62] was detected in the same location in the P2A gene as the experimentally verified CRE of the HRV2 genome ([63]; Figure 9A, Additional file 4, Figure S8A). This appears to be subgroup-specific, in that a similar sequence or structure is not detected among the HRVB genomes in this region (Additional file 4, Fig. S8B). Conversely, a subgroup B-specific CRE-like sequence and structure can be detected in the same location in the VP1 gene as the empirically defined CRE from the HRV14 genome, but not in the HRVA genomes ([64,65]; Figure 9B, Additional file 4, Figures S8C and S8D). Overall, these elements possess essentially identical structures, with loop sequences that vary according to HRV subgroup (Figure 9).

## Discussion

Here, we have addressed a gap in our understanding of the evolutionary forces driving diversification of HRV and deepened our understanding of HRV biology in a number of ways. First, we have augmented the set of 6 fully sequenced HRV serotypes to a more representative subset of 34 genomes from across the HRV phylogeny. Second, we have performed a comprehensive analysis of the genetic diversity and evolutionary pressures operating upon the HRV genus. We have found a uniform pattern of genetic variability across the genome that is unlikely to be driven by large-scale recombination events as has been observed among other genera of the picornavirus family. We have also obtained a molecular portrait of the HRV genomic evolutionary landscape, which has revealed clusters of diversifying residues in both structural and non-structural genes cast against a background of purifying selective pressure. Finally, we have provided insight into the possible functional relevance of the detected diversifying pressure in both the structural and non-structural genes of HRV through comparison of the overlap in these residues with structural and functional domains previously characterized in HRV.

### Correlation in genetic and phenotypic subgroupings of HRV

Our results indicate that the 2 major genetic subgroups of HRV correlate directly with phenotypic groupings based on *in vitro *studies of HRV susceptibility to a set of early generation "pocket factor" binding drugs that interact with the capsid gene products of the virus [14]. This puzzling correlation between pocket factor susceptibility and the genetic relationships of non-structural genes in HRV was first noted almost 20 years ago in the original drug susceptibility study when only a limited set of non-structural gene sequences were available [14]. More recent subgenomic sequence analyses have largely corroborated these findings [25,26,28]. Here, we extend these results to every locus of the HRV genome.

In general, this observation has been somewhat difficult to understand since these drugs could not have shaped HRV evolution, given that they have not been commonly used to treat viral infections in general, or HRV infections in particular. Our results provide a possible explanation. Because there is a consistent level of sequence diversity across the HRV genome, each locus in the genome possesses a genetic relationship identical to that of the structural genes targeted by the drug. Thus, the correlation between genotype and drug susceptibility phenotype is easily detectable at each loci in the genome, regardless of its potential to interact directly with the drug.

### Recombination and diversification in the HRV genome

Our analysis has also revealed a lack of significant recombination within the HRV genome that is surprising in light of the fact that multiple serotypes that utilize the same cellular receptor are known to co-circulate during each HRV season [66]. Moreover, this is also quite distinct from what has been observed for other genera in the *Picornaviridae *family, where recombination has been proposed to play a significant role in genetic diversification (reviewed in [39]). Taken together, our results favor the possibility that genetic drift is likely to be the major driving force for diversification in the HRV genus. These conclusions extend and agree with the recent work of Simmonds [43]. It would appear that the known HRV isolates act as independently segregating genomes, with little potential for inter-genome recombination, in contrast to the non-segregating, highly recombinant genomes such as HEV, FMDV, the teschoviruses, and bovine enteroviruses.

Furthermore, it has been hypothesized that there is a biological compatibility barrier for recombination among HRV serotypes, since experimental evidence has demonstrated recombinants from similarly diverged picornaviruses tend to be inviable (reviewed in [39]). It is also possible that there may be additional barriers related to the characteristics of HRV infection (intracellular partitioning, persistence time in the cell, viral titer, blocks to co-infection, etc) that might preclude the opportunity for recombination to occur. With a diverse array of HRV genome sequences in hand, such hypotheses can now be directly tested.

### Purifying selective pressure dominates in the HRV genome

Despite a notoriously error-prone polymerase and a significant amount of genetic diversity across the HRV genome, our selective pressure analysis indicates that overall, the HRV genome is under strong pressure to preserve the amino acid sequences encoded within genome. This sort of profile is not unique to HRV, since a similar bias towards purifying selection has been detected in selective pressure analysis of the capsid region of FMDV field isolates [67]. A preponderance of purifying selective pressure is particularly obvious for the central region of the genome encoding the non-structural P2 gene products (P2A protease, P2B 'viroporin', and P2C ATPase and membrane association factor) and the 3A and 3B gene products. Each of these viral gene products is known to proteolyze or to interact with essential cellular factors, which are highly conserved. Thus, it may be that the lifecycle of HRV and its requirement to interact with and inactivate a variety of host factors results in significant sequence constraints within this portion of the genome.

Although these results may appear to contradict recent studies demonstrating that at least one *Picornaviridae *family member, poliovirus, evolves through quasispeciation [68], they actually do not rule out a similar process occurring in HRV. Rather, our results reflect the overall selective pressure acting on the HRV genome derived from the consensus sequences generated from our shotgun assemblies, and we have not focused on the potential minority polymorphisms that may exist within the population of each of the HRV prototypes. Inspection of each of our shotgun assemblies does reveal high quality sequence polymorphisms in a minority of the shotgun reads throughout the assembled genomes (data not shown). However, a greater depth of sequencing for each isolate would be required to unambiguously address the extent of HRV quasispeciation.

### Implications of diversifying selective pressure in the structural genes

Although we detected overlap with each of the functional domains found on the viral particle, the diversifying capsid residues overlap significantly only with previously identified antigenic sites from both the HRVA and HRVB genomes. This result is intriguing in light of the variability in genetic diversity and serotype diversity known to exist in some of the *Picornaviridae *family members, such as the FMDVs and HEVs. The FMDVs are similar to HRVs, in that over 100 distinct serotypes have also been identified [38]. These observations suggest that the icosahedral viral particle of these picornaviruses is relatively flexible, and is able to accommodate a wide array of non-synonymous changes. However, this immunogenic diversity is not generally shared among the capsids of all *Picornaviridae *family members. In particular, poliovirus has only 3 characterized serotypes. Moreover, recent analysis of vaccine-derived poliovirus isolates indicates that many of the most frequent non-synonymous changes which develop in the capsid genes do not alter the immunogenicity of the virus, despite being present in antigenic determinants [69]. It is unclear if these results are unique to poliovirus or extend to other picornaviruses.

This is particularly relevant for our analysis, since we were unable to explain all of the diversifying selective pressure by direct overlap with antigenic sites on the surface of the viral pentamer. While many of our diversifying residues map within close proximity to these NIms, it is unclear if diversification of sites proximal to NIms actually alters their antigenicity. Such questions are difficult to resolve at this time, since the known antigenic determinants of HRV have been identified through sequence analysis of HRVs able to escape neutralization of a limited set of monoclonal antibodies raised against only 2 of the 102 HRV serotypes [46-52]. Thus, a more complete understanding of the statistically significant proximity detected here between diversifying capsid residues and the NIms awaits more comprehensive characterization of additional distinct antigenic sites on the HRV capsid.

Although not statistically significant, a surprising amount of overlap was also detected between the diversifying capsid residues and the characterized HRV cellular receptor contacts. Whether diversification of in these residues actually alters the functionality of these domains in the capsid, or merely reflects as-yet undiscovered functions, or regions of the HRV capsid that are under immune surveillance is unclear from these observations. However, it has been established that important functional domains in viruses are not excluded from immune surveillance, and that mutations within antigenic targets that overlap functional domains can abolish antibody interaction with little or no impact on interactions required in the functional domain (reviewed in [70]). Whether such observations also apply to this set of diversifying residues requires a more comprehensive understanding of both the antigenic determinants of the HRV capsid as well as the binding affinities to the HRV cellular receptors across different HRV serotypes.

### Implications of diversifying selective pressure in the non-structural genes

Perhaps one of the most surprising results from this analysis was the detection of clusters of diversifying residues within two non-structural genes that perform essential functions during viral replication. Why did we detect any diversifying residues in these genes? We attempted to investigate this question through similar mapping of the location of the diversifying residues onto available crystal structures of the 3C protease and 3D polymerase. As was observed for the diversifying capsid residues, the diversifying residues in both the 3C protease and 3D polymerase map to surface-exposed residues; however, here we observed less of a bias towards a particular location or functional domain on the surface of each of these factors. We did detect a large proportion of the diversifying residues in the 3C protease and 3D polymerase positioned in the vicinity of characterized domains that are likely to influence RNA/VPg primer binding (for 3C protease) or hypothesized oligomerization domain interactions, protein binding and/or the coordination of subdomain movements that have been hypothesized to influence catalytic activity (for 3D polymerase).

However, the remaining fraction of the diversifying residues within these non-structural genes map to regions in each of these factors for which functions have not yet been assigned. We have not detected a correlation between the 3C protease and 3D polymerase diversifying residues with MHC class I presenting peptides detectable in 3C and 3D. Likewise, we were also unable to detect any correlation between variation in electrostatic potential on the surface of the 3C protease and 3D polymerase, or significant covariation with any other diversifying residues in the genome. Thus, the role these diversifying residues may play in specific functions of the 3C protease and 3D polymerase, or in overall viral fitness, requires further exploration.

Such studies are particularly relevant given recent discoveries highlighting our incomplete knowledge of the functional domains within these two factors. Recently, a previously uncharacterized region of the poliovirus 3D polymerase lying outside the catalytic domain was shown to influence polymerase activity and thus fidelity [58,59,68]. Similarly, mutational analysis of the poliovirus 3C protein has recently uncovered a number of residues required for viral replication and VPg binding that happen localize outside the defined protease and RNA binding/VPg primer binding domains but in proximity with these unassigned diversifying residues, (C.E. Cameron, personal communication). Additional progress in structural analysis of the poliovirus 3CD precursor also indicates potential intersubunit (3C–3D) and intrasubunit (3D–3D) interactions in domains of the 3C and 3D subunits within close proximity to a number of the diversifying residues we have identified within regions of currently unassigned function [71]. A complete understanding of the possible functional role that these diversifying residues may play in either of these individual factors or the active 3CD precursor awaits additional functional studies. The convergence of our results with these independent studies suggesting novel functional domains and interactions within the non-structural genes points to the utility of selective pressure analysis to uncover potentially important functional domains within a genome that may influence viability and overall fitness.

### Conservation of essential non-coding RNA elements in the HRV genome

Analysis of RNA elements present in both the non-coding (5' cloverleaf and IRES, and 3' stem-loop element) and coding regions (CRE) of the HRV genome indicates conservation of both sequence and secondary structures in these regulatory elements in both HRVA and HRVB genomes. Although the consensus secondary structures among these elements appear similar to those generated based on a much smaller set of HRV genome sequences [61], subtle sequence variations can be detected between the HRVA and HRVB subgroup members, as well as within each of the subgroup members (Additional file 4, Data S3 and S4). Such differences are of particular interest as these elements have been shown to be essential for viral replication, translation, overall viability, and in the case of poliovirus, for pathogenicity and tissue tropism [72-75]. Comprehensive analyses of the functional implications and associated clinical implications of diversity in sequence and secondary structure of these regions of the HRV genome have not been performed. Correlations in variation of the known functions of these RNAs with the sequence variation and structural diversity found within this subset of HRVs will shed light on the role they play in viral growth and replication, and may further clarify the role non-coding regions in HRV pathogenesis.

### Potential role for selective pressure analysis in drug development

To date, two drugs targeting conserved regions of the HRV genome have advanced to Phase III clinical trials. Pleconaril, a potent capsid inhibitor of HRVs and HEVs, binds to a surface-accessible hydrophobic pocket in the VP1 protein on the external face of the viral particle [45]. Ruprintrivir targets the proteolytic active site of the 3C protein and exhibits broad inhibition of HRV growth in vitro [45].

Unfortunately, neither of these drugs has demonstrated sufficient symptom relief, or in the case of pleconaril, exhibited untoward interactions with other drugs. Thus, FDA approval was not granted for either of these potential therapies. Moreover, pleconaril treatment has been shown to give rise to drug resistant viruses at a low frequency [76]. This has not been observed with rupritrivir. Such observations can be explained in the context of our selective pressure analysis. Inspection of our data for the residues targeted by these two drugs reveals only a single residue to possess diversifying selective pressure above background (Additional File 3, Tables S2 and Additional file 4, Table S3). This residue lies within the pleconaril binding site and corresponds to VP1 residue 191. Prior work identified this residue to be one of two residues that varied from the consensus valine in pleconaril susceptible HRV serotypes to leucine in resistant HRV serotypes [55]. In fact, a V191L mutation engineered in a susceptible HRVB serotype was found to be sufficient to confer a 30-fold reduction in susceptibility to pleconaril [55].

Having identified the only residue known to yield pleconaril resistance, these results illustrate the potential utility of selective pressure analysis with respect to drug development. In early stages of drug development, selective pressure analysis combined with assays for drug efficacy and viral pathogenicity could prove valuable in *de novo *choice of drug targets. The diversifying potential of residues within or flanking drug binding sites could be evaluated *in silico*, and mutations in such residues could be engineered and assayed for drug binding, normal substrate binding, and viral growth. Ultimately, incorporating such analysis in the drug development pipeline may allow the avoidance of targets with high potential for drug resistance or increased virulence.

## Conclusion

This analysis has closed a gap in our understanding of the genetic diversity and evolutionary pressures across the HRV genome. It has provided a deeper understanding of the similarities and differences between the genetic diversity present in HRV compared to other genera of the picornavirus family. These results have also raised several testable questions related to several domains of unknown function and HRV evolution itself. Ultimately, such knowledge may serve to elucidate the determinants of pathogenicity within the HRV genome and aid in the development of therapeutics to reduce or eliminate the clinical symptoms associated with this ubiquitous respiratory pathogen.

## Methods

### Isolation of RNA from low passage HRV prototype stocks

Low passage tissue culture supernatants from tissue culture cells infected with the HRV serotypes (indicated in Additional File 1, Figure S1 and Additional File 5, Table S4) were obtained from the California Department of Health Services (CaDHS). Supernatants were centrifuged briefly to pellet cellular debris, then passed through 0.2 μm filters, brought to 10 mM CaCl_2_, and incubated with 600 units of micrococcal nuclease (Fermentas) for 3 hours at 37°C. RNA was then isolated from the culture supernatants via Trizol:chloroform extraction, followed by isopropanol precipitation.

### Amplification and shotgun sequencing of HRV prototype stock RNA

RNA isolated from HRV prototype culture supernatants was reverse transcribed, randomly amplified as previously described [77], and cloned into the pCR2.1 TOPO TA vector (Invitrogen) to generate plasmid libraries for each HRV serotype. The resulting libraries were transformed into bacteria. Plasmid DNA prepared from each library of transformants was sequenced using the Big Dye terminator v. 3.1 (Applied Biosystems) containing either -21 universal or -28 reverse primer and analyzed on an ABI 3730xl sequencer (Applied Biosystems).

### Shotgun sequence analysis and assembly of HRV genomes

Approximately 7 Mb of DNA derived from 14,208 reads, with an average length of 500 bp, were shotgun sequenced to generate the initial HRV genome assemblies. Contaminating human and bacterial reads (60% of all reads) were identified and removed through BLAST analysis [78]. A total of 8,278 viral reads were processed and assembled with the CONSED software suite [79]. Overall, each genome assembly contained an average of 304 input viral reads, with an average read depth of 22, and average quality score of 86.4 (Additional file 5, Table S5). Specific PCR was performed to obtain sequences at the extreme 5'end and 3'end of each genome sequenced and to close any internal gaps. For the ends, a single high quality (minimum phred score of 20) sequencing read with at least 100 nucleotides of overlap with the shotgun assembly reads was required to consider each genome finished. For the internal gaps, a minimum of 2 high quality forward and reverse reads with overlap of at least 100 nucleotides with shotgun contigs were required to consider internal gaps closed. A shotgun sequence assembly derived from the previously sequenced HRV001b [32] was used to validate the quality of sequences obtained by these methods. The resulting shotgun assembly of HRV001b was 99.6% identical (6198 identities of 6223 nucleotides assembled) to the fully sequenced HRV001b present in NCBI (genbank identifier 221708).

### Sequence alignment and phylogenetic analysis

Inferred amino acid sequence of the coding regions of the 34 complete HRV genomes were aligned using the CLUSTALW program [80]. This alignment was then back-translated into nucleotide sequence and combined with alignments of the 5' and 3' non-coding regions, generated using CLUSTALW, to form the whole-genome nucleotide alignment used for analysis. Neighbor-joining phylogenetic trees were generated from the alignment using CLUSTALW with Kimura's correction for multiple base substitutions. Maximum likelihood trees were generated using baseml from the PAML [81] package and DNAML from the Phylip [82] package. Trees generated using neighbor-joining and maximum likelihood methods contained similar topologies, and differed only in computed branch lengths. The HKY85 model of nucleotide substitution was used, and the values of the transition/transversion rate and the alpha parameter in baseml were estimated through maximum likelihood calculation. Alignment positions with gaps were ignored in all cases.

Scanning average pairwise sequence identity plots were generated using a moving window of 100 nucleotides or 50 amino acids across the whole-genome nucleotide alignment and the corresponding amino acid translation in the coding region of the genome.

### Recombination analysis

The genomic nucleotide alignment of the 34 complete HRV genomes was analyzed using RDP version 2 [83]. Six automated recombination analysis algorithms were run: RDP, GENECONV [84], BOOTSCAN [85], MaxChi [86], Chimaera [87], and Sister Scanning [88]. These algorithms were selected from the set of published recombination detection methods based on their ability to identify recombinant sequences, the associated breakpoints, and parental sequences. In computational and empirical comparative tests, no single method performed best under all conditions, and consistent results from more than one method was the best indicator of recombination [87,89]. Resulting predictions of recombination events with p-values less than 0.05 were analyzed manually using all six methods. Events supported by evidence from more than one method were further characterized by manual analysis of bootstrapped phylogenetic trees of the relevant genomic locus to determine the genotypes involved in the recombination event.

### Selective pressure analysis

Codon-based models of evolution of coding sequence allowing for variable selection pressure among sites in a maximum-likelihood framework were used to evaluate the selective pressure operating on each gene individually. Codon-substitution models [90,91] were compared using likelihood ratio tests (LRT) to test for significant diversifying selection within each gene.

These codon-substitution models, allowing for variable ω (dN/dS) parameters among sites, were fit to the nucleotide alignment of the coding sequence of the genome. Model M1a, or the neutral model, incorporates a class of sites under purifying selection with ω_0 _< 1, and a second class of sites with ω_1 _= 1. Model M2a adds a third class of sites ω_2 _> 1, to allow for diversifying selection. Similarily, Model 7 incorporates a discrete beta distribution (10 classes) to model values of ω between 0 and 1, while Model 8 adds an additional parameter ω > 1. Likelihood ratio tests were performed between nested models (M1a versus M2a, or M7 versus M8) to calculate the significance of diversifying selection within a gene (Additional file 5, Table S6). An empirical Bayesian approach was then used to calculate the posterior probability that a site belongs to each of the ω site classes. This probability value was then used to compute an estimate of dN/dS for each site in the sequence. Maximum likelihood calculations on the substitution models were implemented using the codeml program from version 3.14 of the PAML package [81].

To ascertain how well the resulting dN/dS values computed from the subset of 34 reference genomes reflected the selective pressure present in the full set of 102 known HRV serotypes, we compared the dN/dS values computed for each residue in the VP1 gene of this set of HRVA and HRVB serotypes to the same dN/dS values obtained independently from the available VP1 sequences of all 102 HRV serotypes [25,26]. Although the absolute value of the dN/dS ratios differed between the two sets, their relative rankings were well correlated (0.91 and 0.80, for HRVA and HRVB genomes, respectively; Additional file 1, Figure S2), with few potential false positives and false negatives detected. Thus, it appears that the relative rank, rather than absolute magnitude of the dN/dS values we have computed from this subset of HRV genomes accurately approximates the selective pressures detectable among the full set of 102 HRV reference serotypes.

Tests of heterogeneous synonymous substitution rates among sites were performed using the REL analysis implemented in the HYPHY [92] phylogenetic package. This method of analysis is very similar to that described above, but differs in codon models available, and in the modeling of site classes (REL site classes are modeled as N discrete classes, similar to model M3 in codonml). Analysis using the GY [93] model of codon evolution with six discrete classes of non-synonymous and synonymous mutation rates was used to determine the effects of variable dS across sites on the data. Although varying dS resulted in a lowered magnitude of a number of capsid residues in the smaller dataset of HRVB genomes, it did not significantly impact the per-residue dN/dS values for the HRVA genomes or confer any significant changes in the overall identity or localization of the 5% highest scoring dN/dS residues of the capsid genes (Additional file 5, Figure S9). Thus, for the sake of simplicity, dN/dS values discussed in the results section were those derived from the calculations described above assuming a homogeneous synonymous substitution rate.

### Mapping dN/dS values onto 3-dimensional crystal structures

Viral pentamer structures were generated from the NCBI Protein Database (pdb) files of HRV2 (pdb id 1FPN), HRV14 (pdb id 4RHV), and HRV16 (pdb id 1AYM) using the Oligomer Generator utility from the VIPERdb website [94]. Analysis of the 3C protease and 3D polymerase was performed using the HRV2 3C protease (pdb id 1CQQ), and HRV14 3D polymerase (pdb id 1XR5), respectively. The molecular structure visualization program, Chimera [95], was used to generate images of the viral proteins.

### Distance calculations

Calculations of the significance of the overlap in structure space between sets of dN/dS data were calculated using an average minimum distance between residues metric. Observed average minimum distance between two sets (A and B) of residues was calculated by taking the average of the minimum three-dimensional Cartesian distance from each residue of set A to the nearest residue from set B. In effect this is a measurement of how closely correlated the positions of set A are to any subset of the positions in set B. To calculate the significance of this observed distance, 100,000 iterations of this calculation were computed, randomizing the locations of the residues in set A for each calculation. The distribution of the resulting average minimum distance values was used to calculate a p-value for the significance of the observed value.

### Accession numbers

The GenBank accession numbers for the sequenced HRV genomes range from DQ473485–DQ473512.

## Competing interests

The author(s) declare that they have no competing interests.

## Authors' contributions

ALK, DRW, HAB, HL, and JLD conceived and designed the experiments. DPS and SY provided reagents/materials and advice to perform experiments. ALK, SR, JJC, VM, and ERM generated whole genome shotgun sequence data. DRW contributed analysis tools. DRW and ALK analyzed the data. ALK, DRW, and JLD wrote the paper.

## Supplementary Material

Additional File 1 Supplemental Figures 1–3, Supplemental Data 1 and 2. Click here for file

Additional File 2 Supplemental Figure 4 and Supplemental Table 1. Click here for file

Additional File 3 Supplemental Figures 5–7 and Supplemental Table 2. Click here for file

Additional File 4 Supplemental Table 3, Supplemental Data S3 and S4, and Supplemental Figure 8. Click here for file

Additional File 5 Supplemental Tables 4-6 and Supplemental Figure 9. Click here for file

Additional File 6 Supplemental Methods. Click here for file
