# Supplementary material for: Genome-wide diversity and selective pressure in the human rhinovirus
Source: Virol J. 2007 May 3;4:40. doi: 10.1186/1743-422X-4-40 (PMC1892812; doi:10.1186/1743-422X-4-40)
Supplement: Additional File 1 — Supplemental Figures 1–3, Supplemental Data 1 and 2. [file 1743-422X-4-40-S1.pdf]

Figure S1 - HRV phylogenetic tree based on aligned VP1 sequences available in the NCBI database.

Neighbor-joining phylogenetic trees based on nucleotide sequences of HRVA (red) and HRVB (blue). Bold text with * adjacent indicates the 6 HRV serotypes with full genome sequence publicly available at the outset of this study. Bold text without star indicates the 27 additional characterized HRV serotypes for which complete genome sequence was obtained in this study. (PDF)

Figure S2 – VP1 dN/dS values computed for all 102 HRV serotypes versus the 34 fully sequenced serotypes.

Scatter plot comparing dN/dS values for each residue in the VP1 gene calculated from all 102 serotypes (x axis) and values calculated from the 34 complete genome sequences (y axis) analyzed. Values calculated from subgroup A sequences in red, values from subgroup B sequences in blue. Spearman rank correlation coefficient values for each dataset are shown. (PDF)

Figure S3 - HRV genomic phylogenetic tree based on deduced amino acid sequences of available HRV genomes.

Neighbor-joining phylogenetic trees based on nucleotide sequences of HRVA (red) and HRVB (blue). Bootstrap values for each node are shown according to color scale indicated in figure. (PDF)

Data S1 - HRV sub-genomic phylogenetic trees based on nucleotide sequence of each locus for available HRV genomes.

Neighbor-joining phylogenetic trees based on nucleotide sequences of the 5'NCR and each gene within the HRV genome. Bootstrap values are indicated at each node. (PDF)

Data S2 –HRV sub-genomic phylogenetic trees based on deduced amino acid sequence of available HRV genomes.

Neighbor-joining phylogenetic trees based on deduced amino acid sequences of each gene within the HRV genome. Bootstrap values are indicated at each node. (PDF)

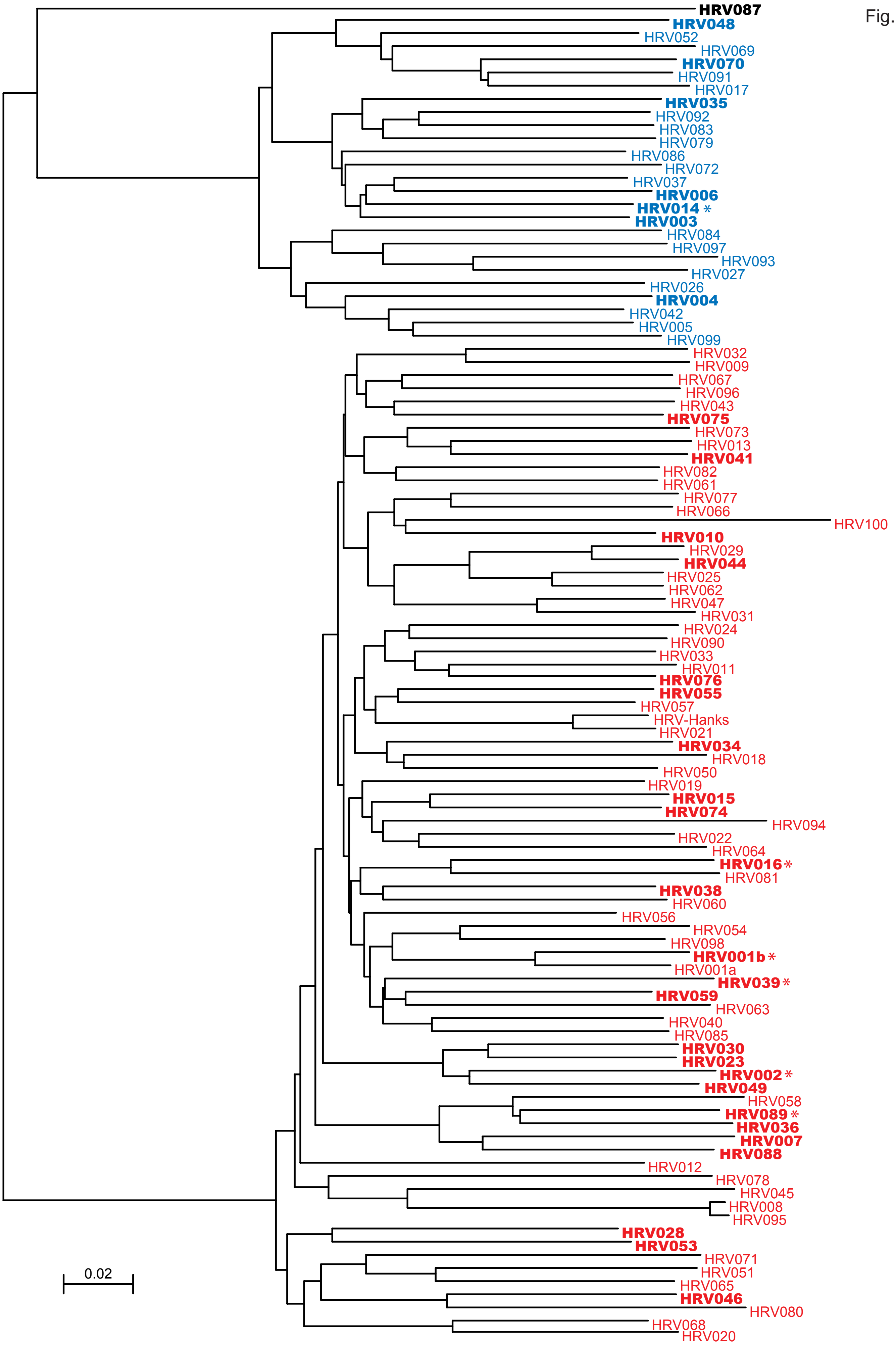
HRV087
HRV048
HRV052
HRV069
HRV070
HRV091
HRV017
HRV035
HRV092
HRV083
HRV079
HRV086
HRV072
HRV037
HRV006
HRV014 *
HRV003
HRV084
HRV097
HRV093
HRV027
HRV026
HRV004
HRV042
HRV005
HRV099
HRV032
HRV009
HRV067
HRV096
HRV043
HRV075
HRV073
HRV013
HRV041
HRV082
HRV061
HRV077
HRV066
HRV100
HRV010
HRV029
HRV044
HRV025
HRV062
HRV047
HRV031
HRV024
HRV090
HRV033
HRV011
HRV076
HRV055
HRV057
HRV-Hanks
HRV021
HRV034
HRV018
HRV050
HRV019
HRV015
HRV074
HRV094
HRV022
HRV064
HRV016 *
HRV081
HRV038
HRV060
HRV056
HRV054
HRV098
HRV001b *
HRV001a
HRV039 *
HRV059
HRV063
HRV040
HRV085
HRV030
HRV023
HRV002 *
HRV049
HRV058
HRV089 *
HRV036
HRV007
HRV088
HRV012
HRV078
HRV045
HRV008
HRV095
HRV028
HRV053
HRV071
HRV051
HRV065
HRV046
HRV080
HRV068
HRV020
0.02

Fig. S1

Fig. S2

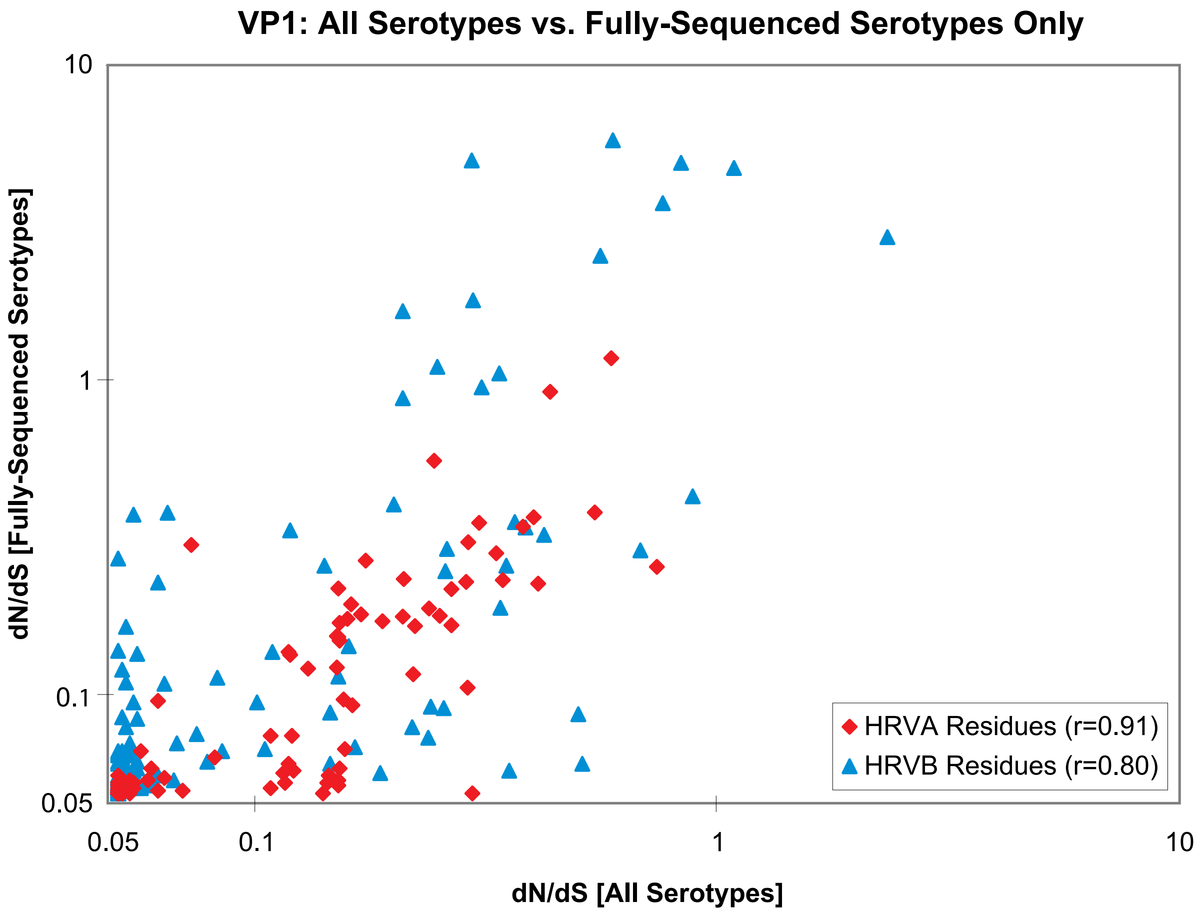
VP1: All Serotypes vs. Fully-Sequenced Serotypes Only
10
1
0.1
0.05
0.05
0.1
1
10
dN/dS [Fully-Sequenced Serotypes]
dN/dS [All Serotypes]
HRVA Residues (r=0.91)
HRVB Residues (r=0.80)

Fig. S3

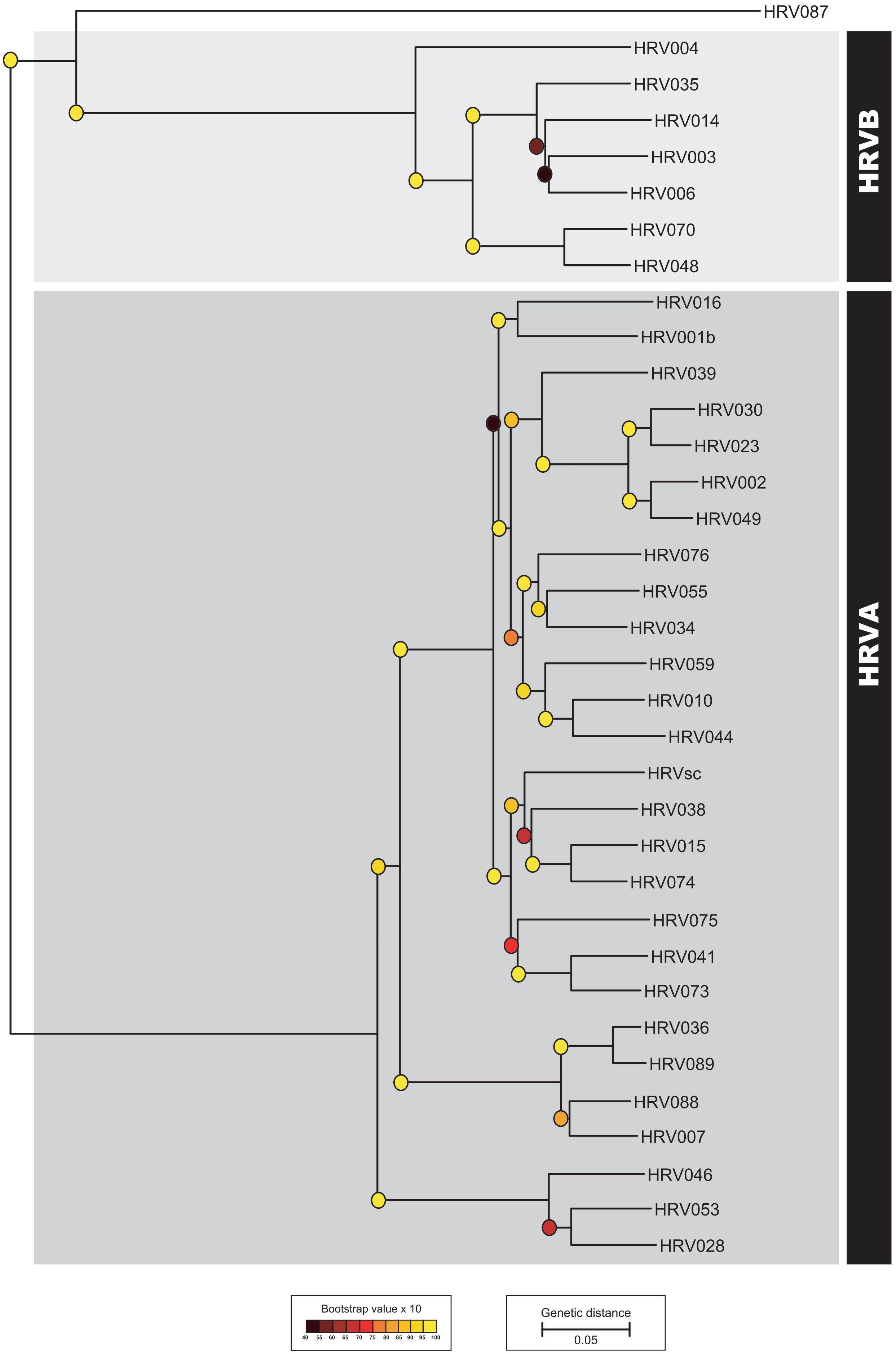

HRV087
HRV004
HRV035
HRV014
HRV003
HRV006
HRV070
HRV048
HRVB
HRV016
HRV001b
HRV039
HRV030
HRV023
HRV002
HRV049
HRV076
HRV055
HRV034
HRV059
HRV010
HRV044
HRVsc
HRV038
HRV015
HRV074
HRV075
HRV041
HRV073
HRV036
HRV089
HRV088
HRV007
HRV046
HRV053
HRV028
HRVA
Bootstrap value x 10
40 55 60 65 70 75 80 85 90 95 100
Genetic distance
0.05

Data S1

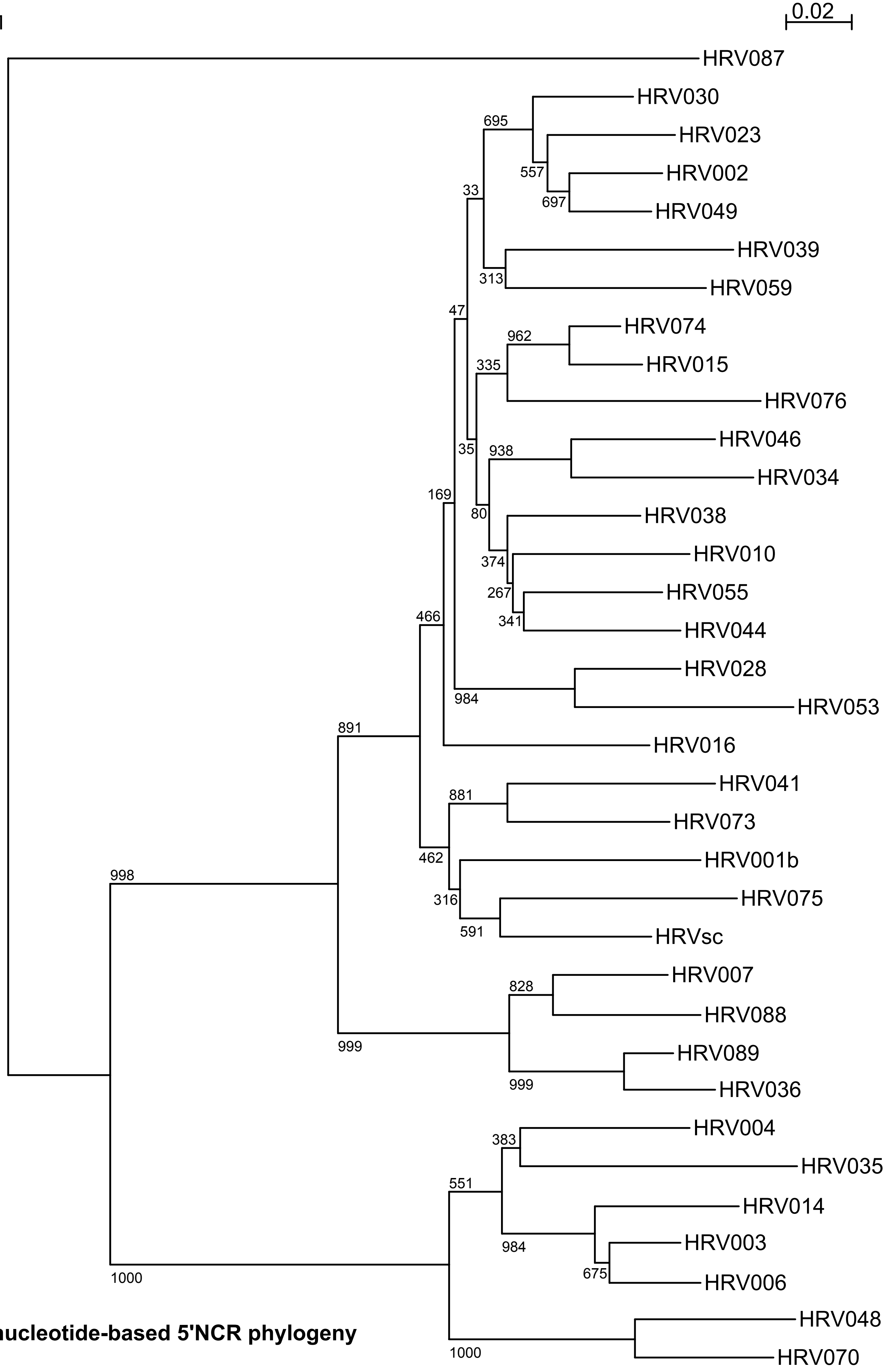

nucleotide-based 5'NCR phylogeny

Data S1

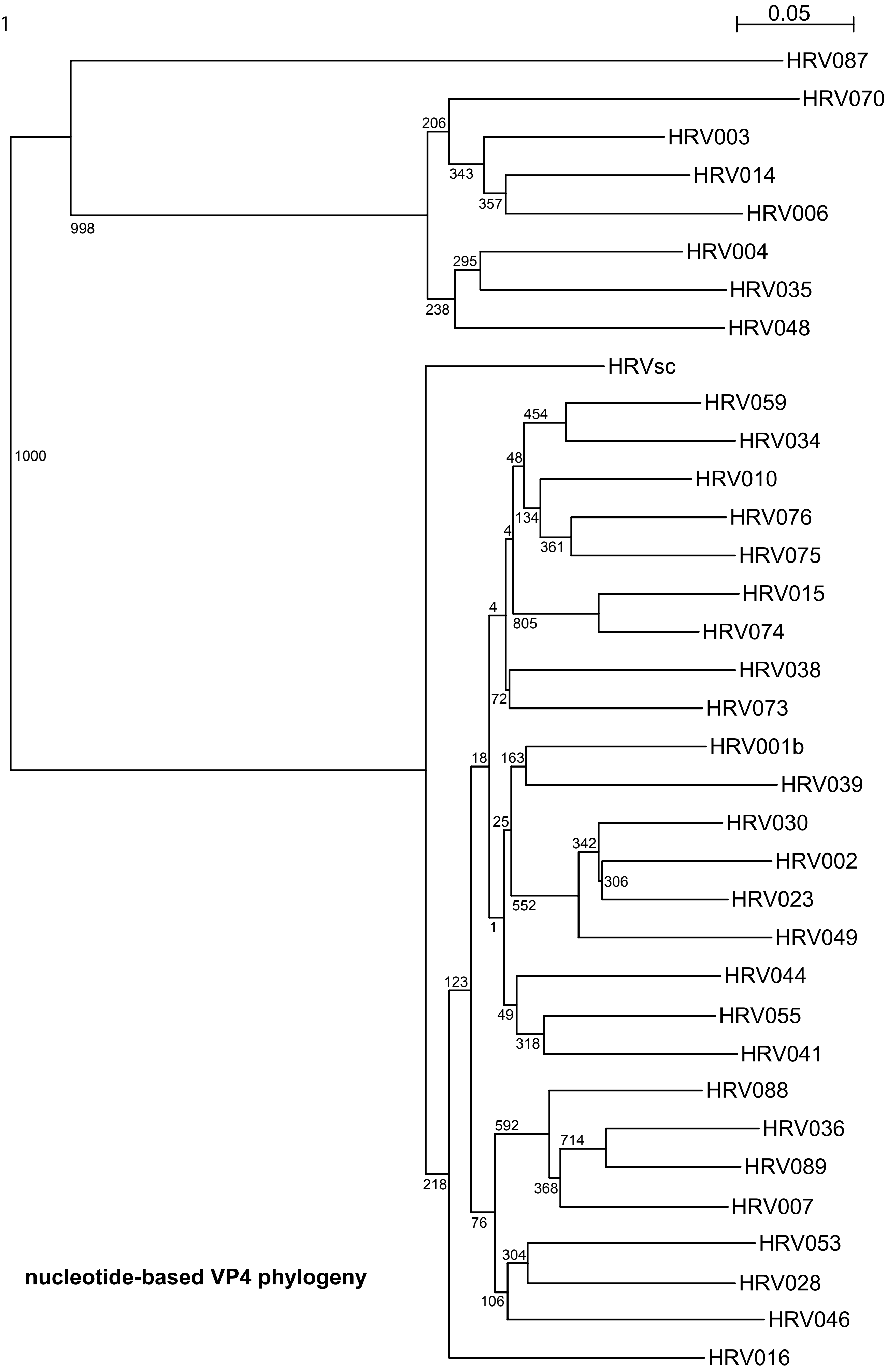

nucleotide-based VP4 phylogeny

Data S1

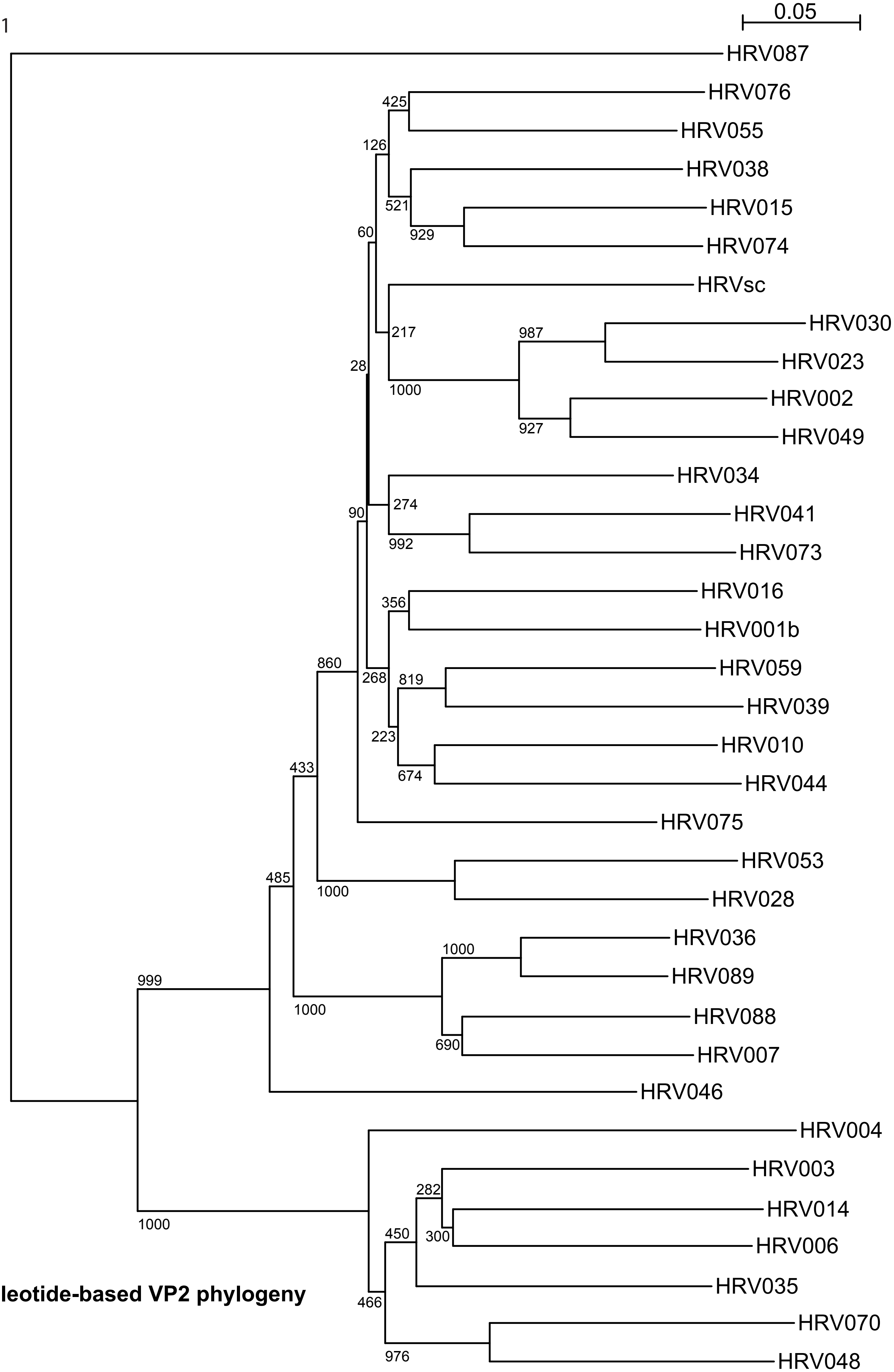

nucleotide-based VP2 phylogeny

Data S1

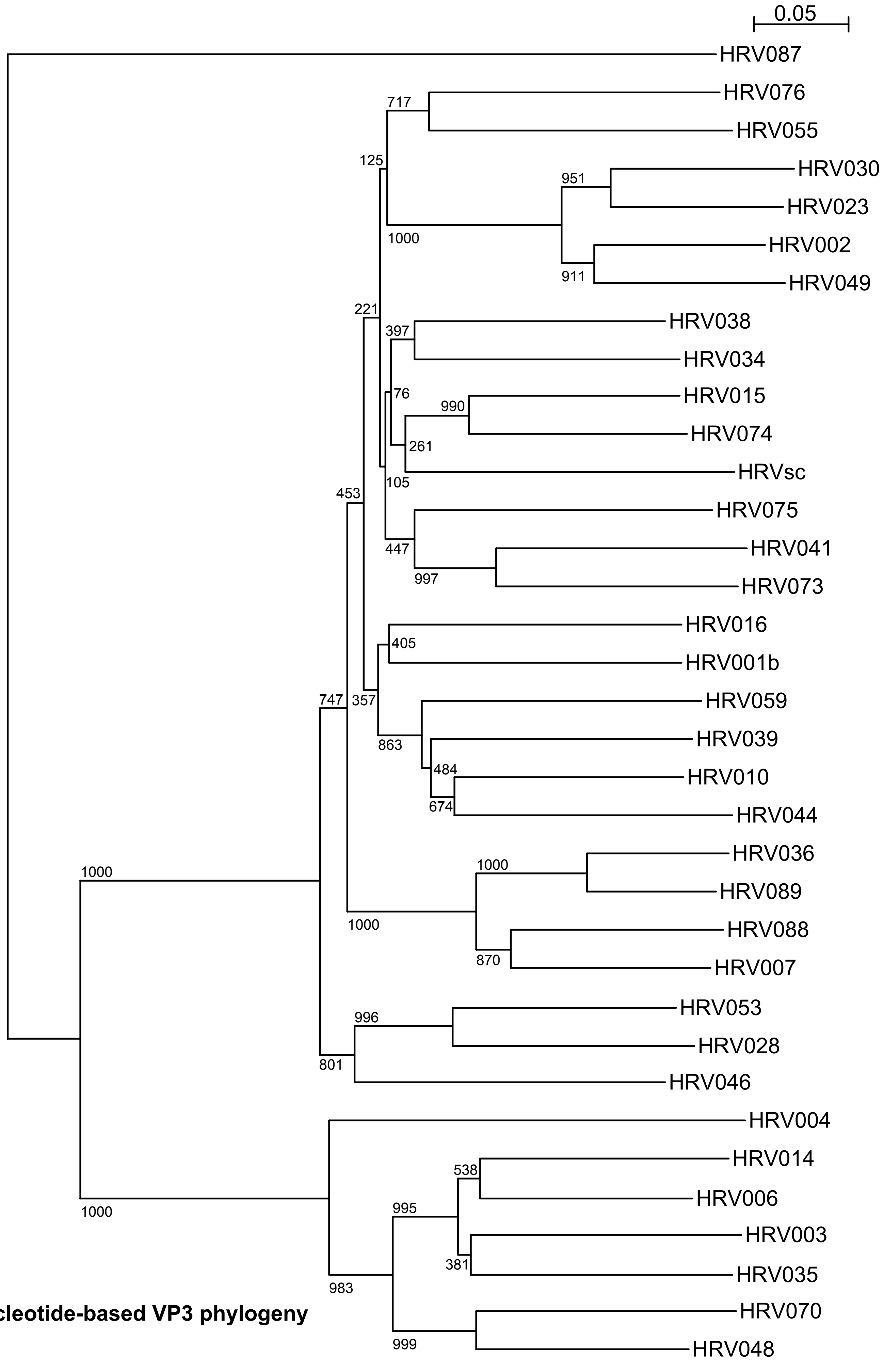

nucleotide-based VP3 phylogeny

Data S1

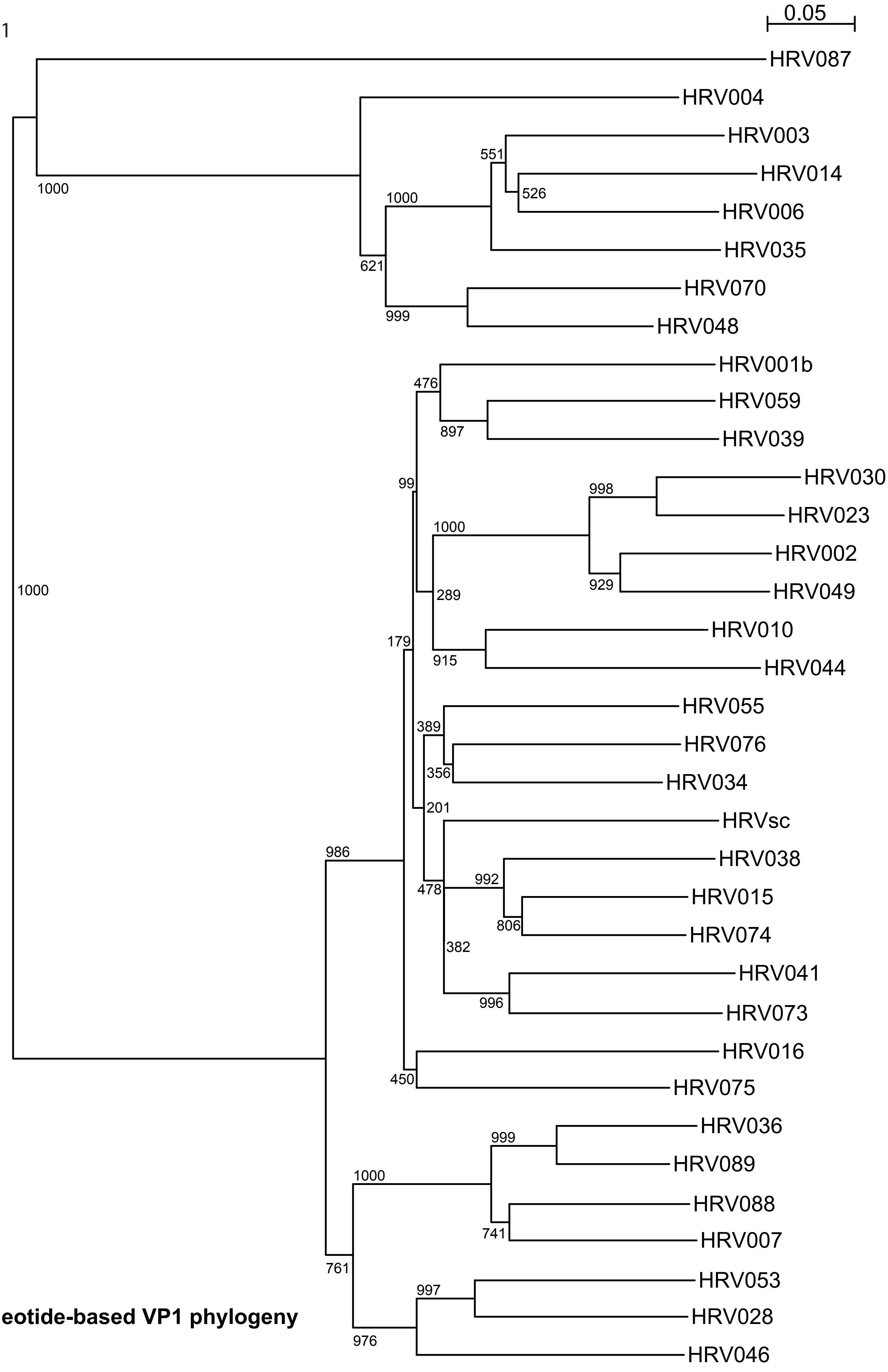

nucleotide-based VP1 phylogeny

Data S1

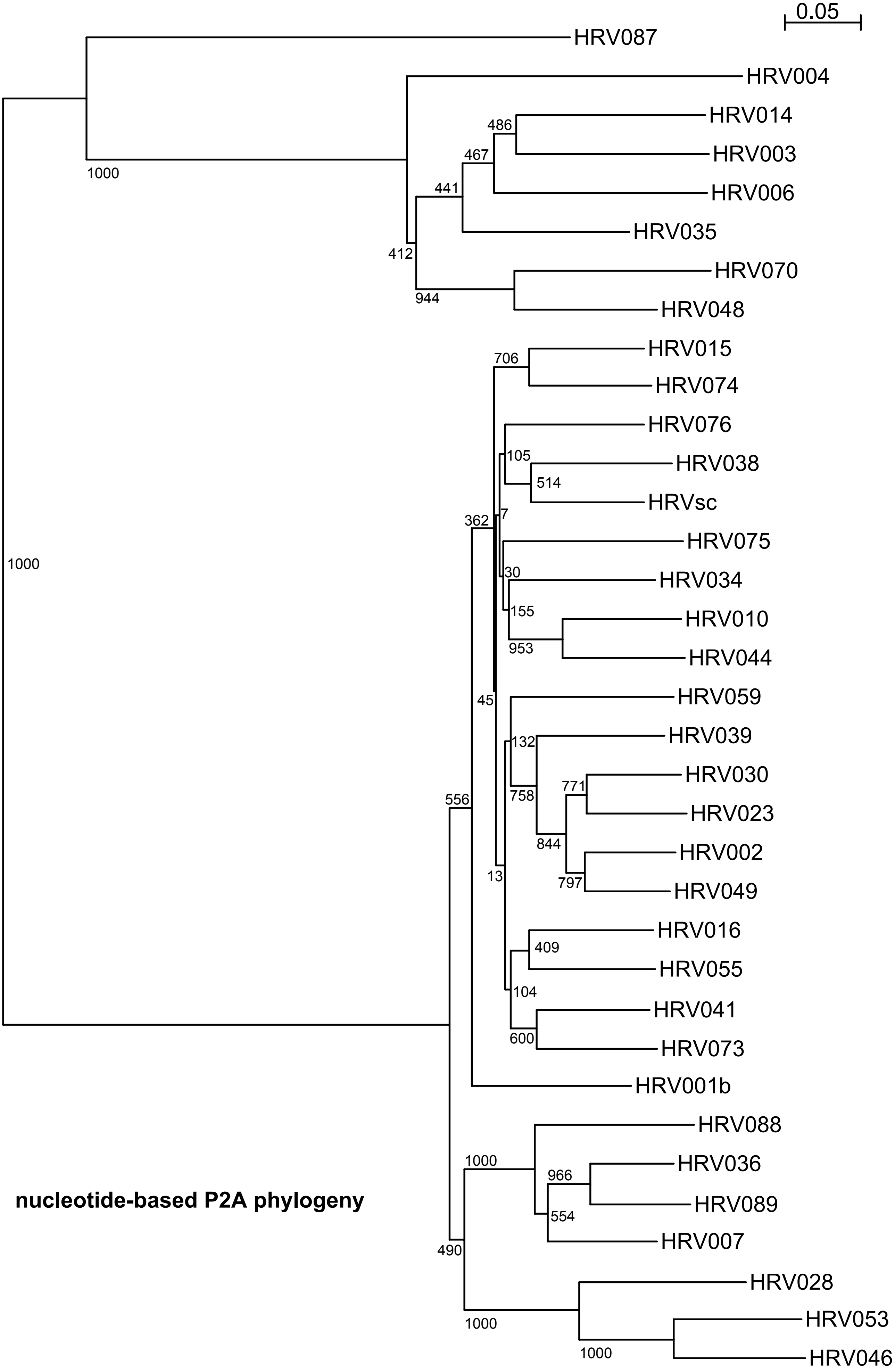
0.05
HRV087
HRV004
HRV014
HRV003
HRV006
HRV035
HRV070
HRV048
HRV015
HRV074
HRV076
HRV038
HRVsc
HRV075
HRV034
HRV010
HRV044
HRV059
HRV039
HRV030
HRV023
HRV002
HRV049
HRV016
HRV055
HRV041
HRV073
HRV001b
HRV088
HRV036
HRV089
HRV007
HRV028
HRV053
HRV046
1000
1000
486
467
441
412
944
706
105
514
362
7
30
155
953
45
132
771
758
844
797
13
556
409
104
600
1000
966
554
490
1000
1000
nucleotide-based P2A phylogeny

Data S1

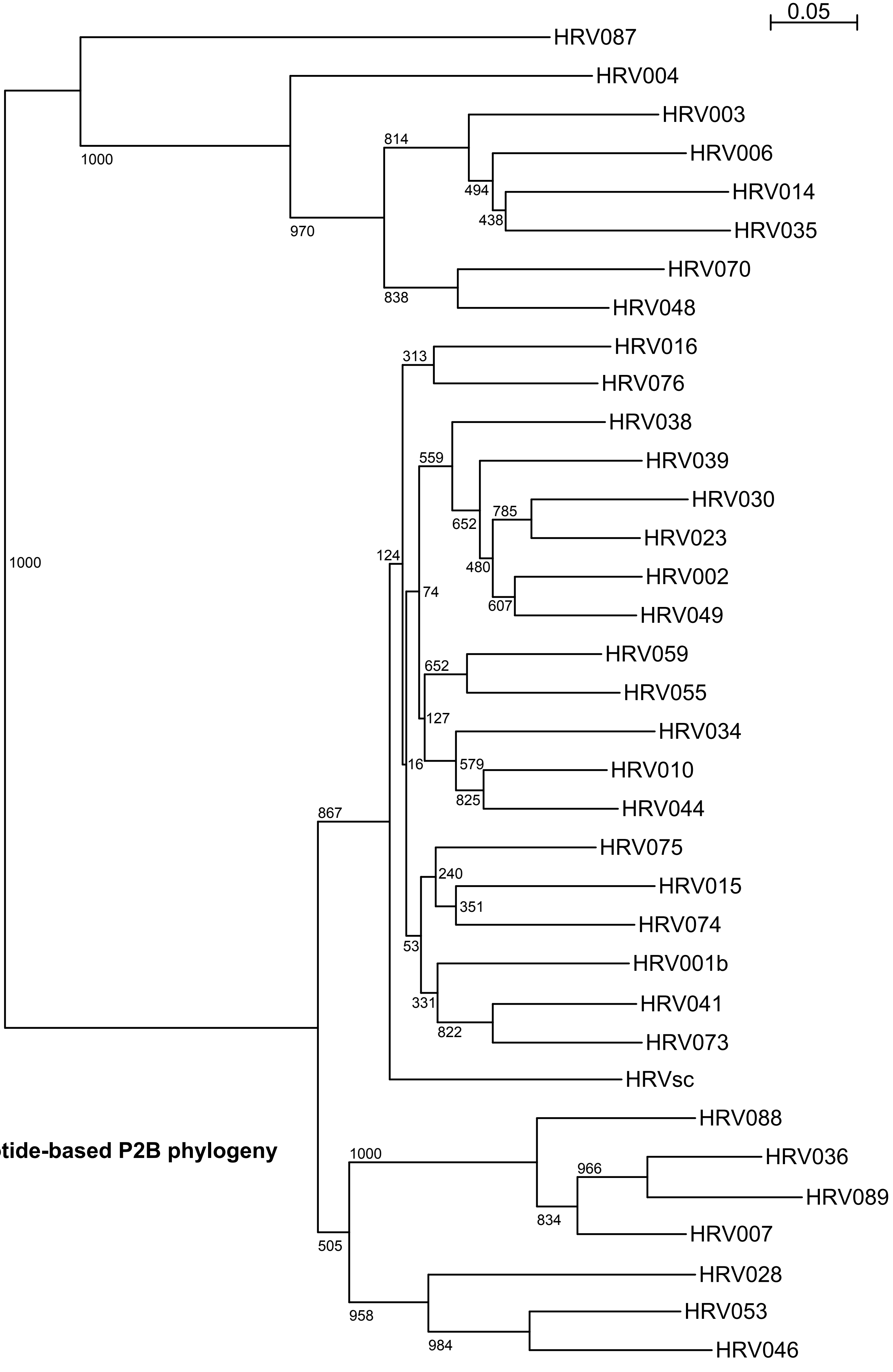
0.05
HRV087
HRV004
HRV003
HRV006
HRV014
HRV035
HRV070
HRV048
HRV016
HRV076
HRV038
HRV039
HRV030
HRV023
HRV002
HRV049
HRV059
HRV055
HRV034
HRV010
HRV044
HRV075
HRV015
HRV074
HRV001b
HRV041
HRV073
HRVsc
HRV088
HRV036
HRV089
HRV007
HRV028
HRV053
HRV046
1000
814
494
438
970
838
313
559
785
652
480
607
124
74
1000
652
127
16
579
825
867
240
351
53
331
822
1000
966
834
505
958
984
nucleotide-based P2B phylogeny

Data S1

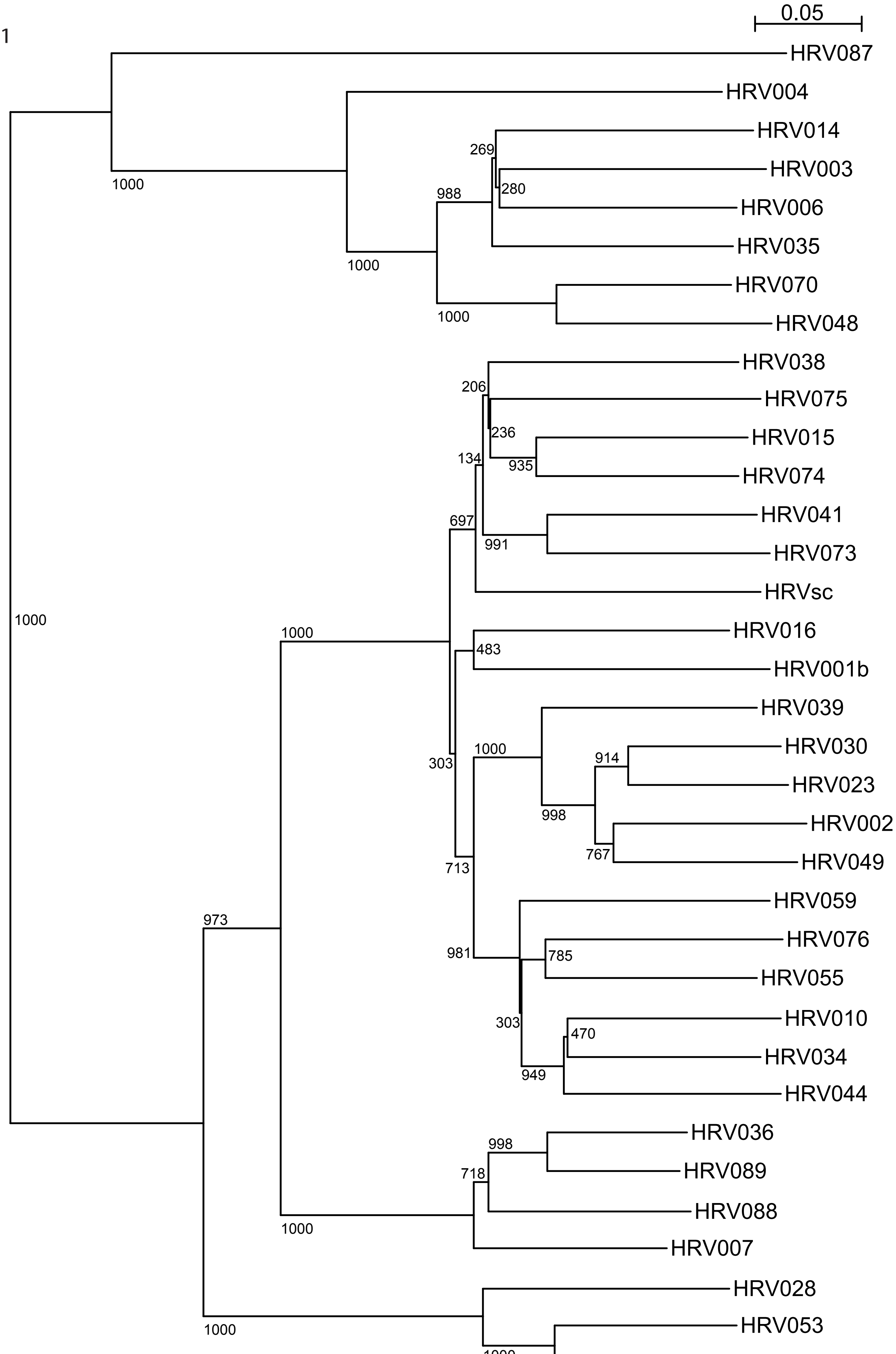

nucleotide-based P2C phylogeny

Data S1

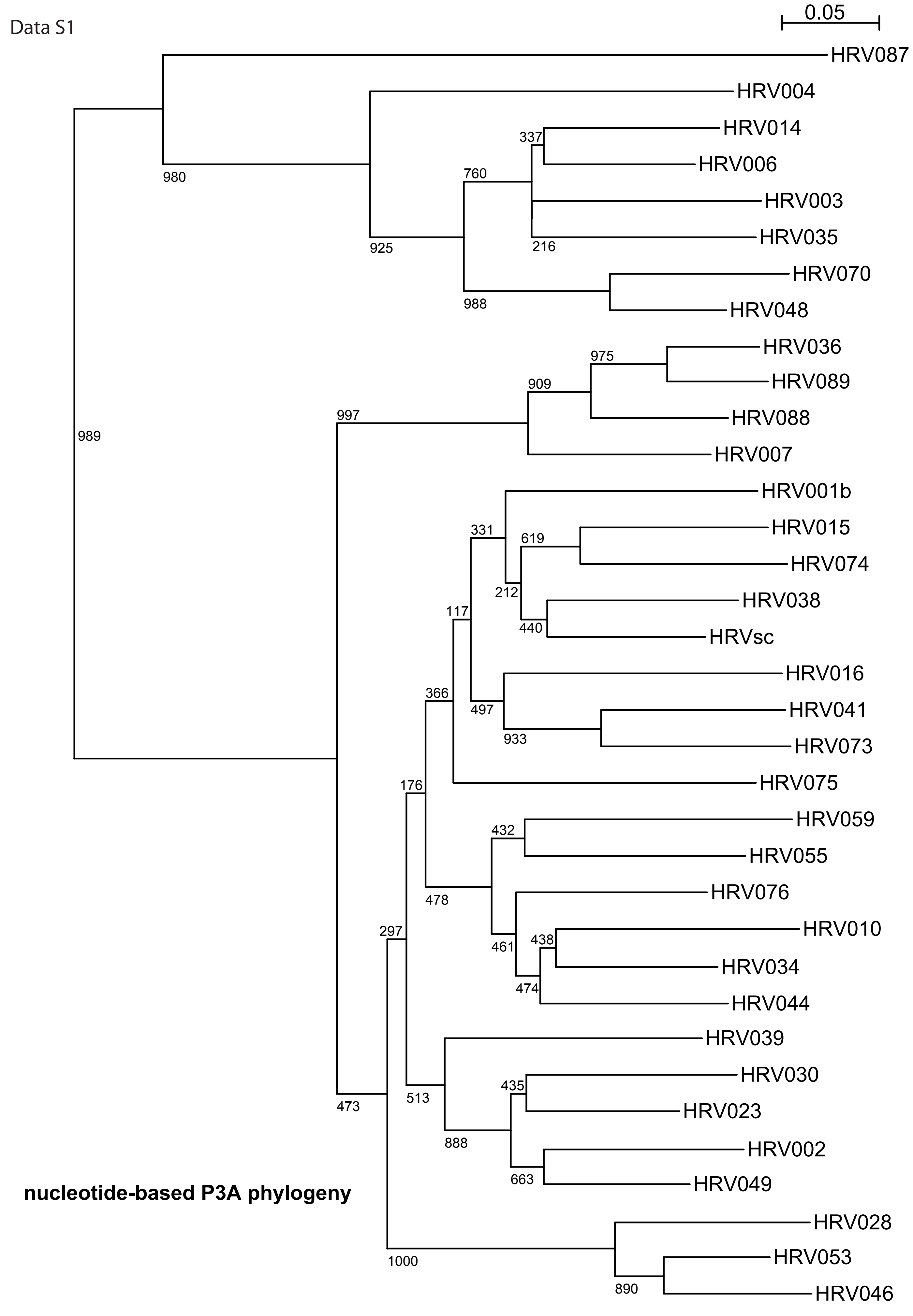
0.05
HRV087
HRV004
HRV014
HRV006
HRV003
HRV035
HRV070
HRV048
HRV036
HRV089
HRV088
HRV007
HRV001b
HRV015
HRV074
HRV038
HRVsc
HRV016
HRV041
HRV073
HRV075
HRV059
HRV055
HRV076
HRV010
HRV034
HRV044
HRV039
HRV030
HRV023
HRV002
HRV049
HRV028
HRV053
HRV046
980
337
760
925
216
988
975
909
997
989
331
619
212
117
440
366
497
933
176
432
478
438
461
474
297
435
513
888
663
473
1000
890
nucleotide-based P3A phylogeny

Data S1

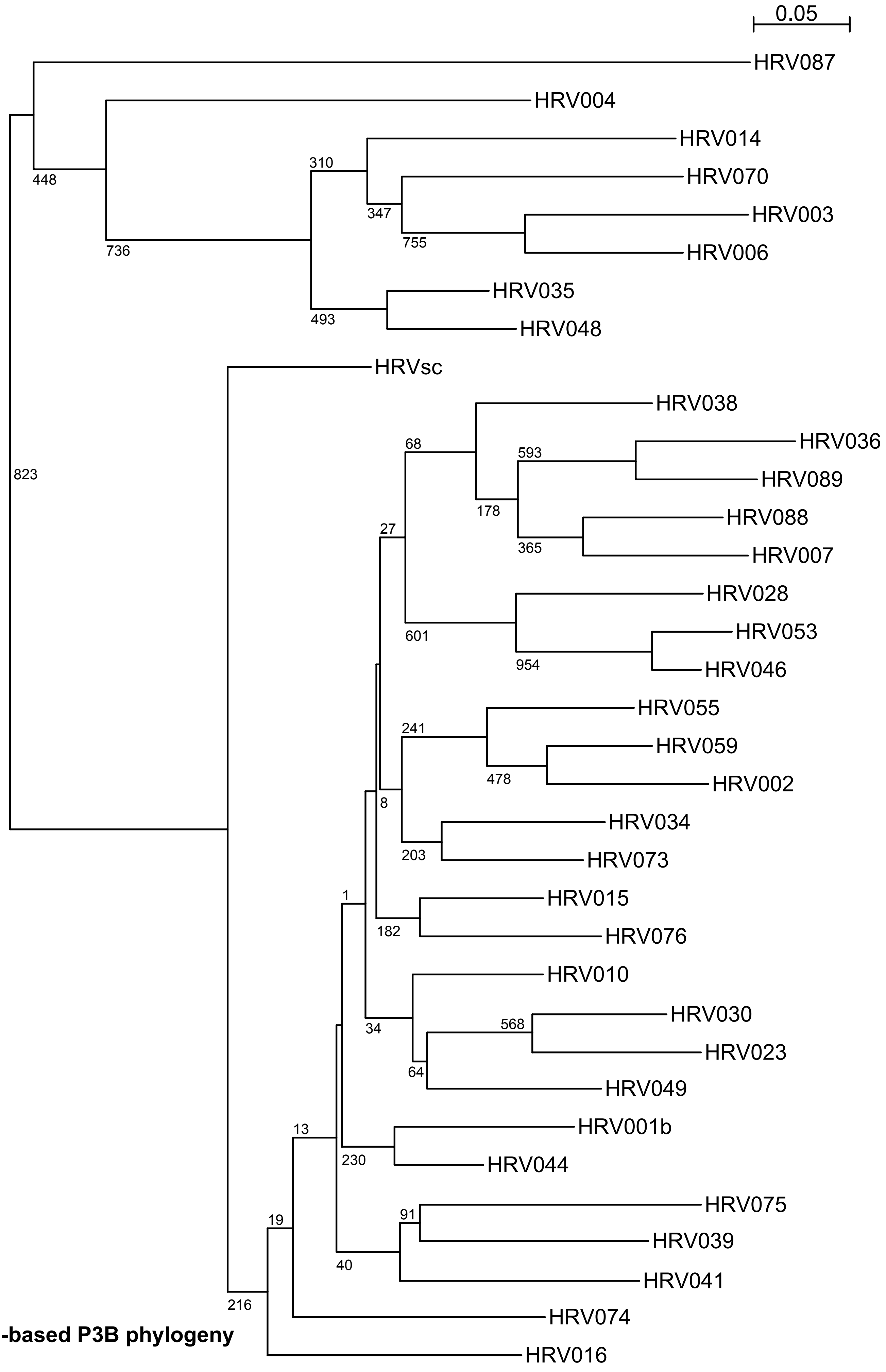

nucleotide-based P3B phylogeny

Data S1

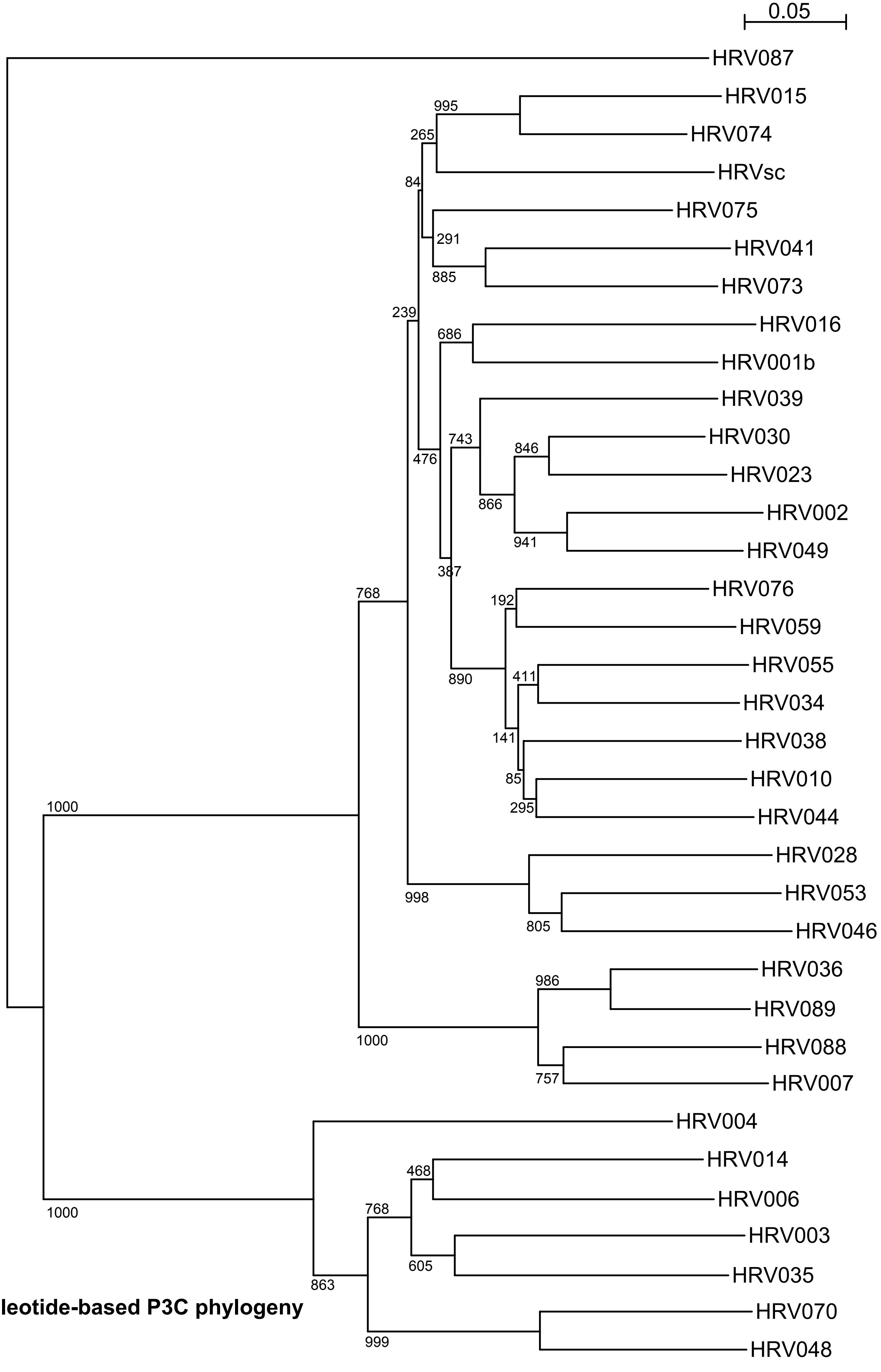

nucleotide-based P3C phylogeny

Data S1

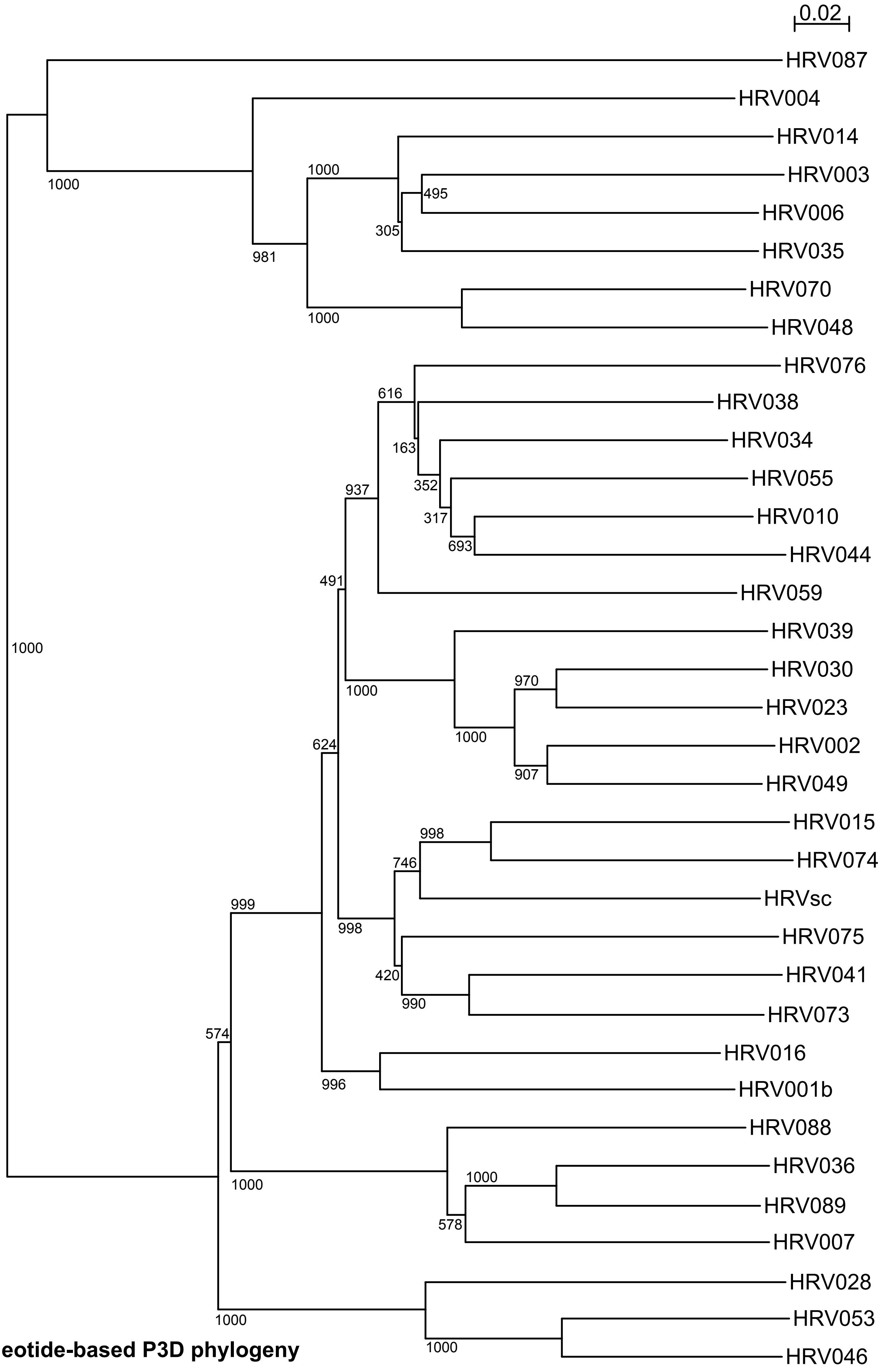

nucleotide-based P3D phylogeny

Data S2

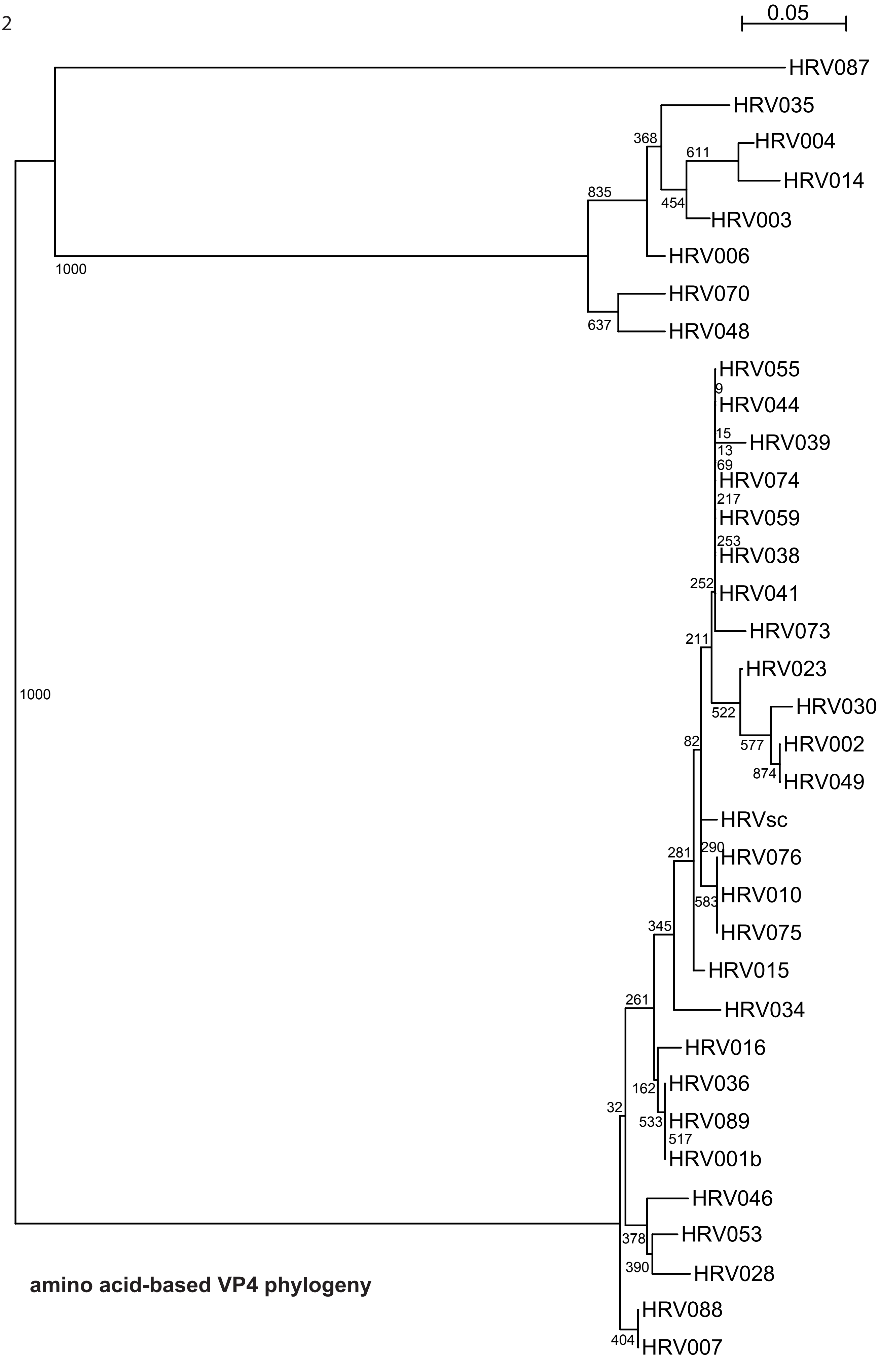

amino acid-based VP4 phylogeny

Data S2

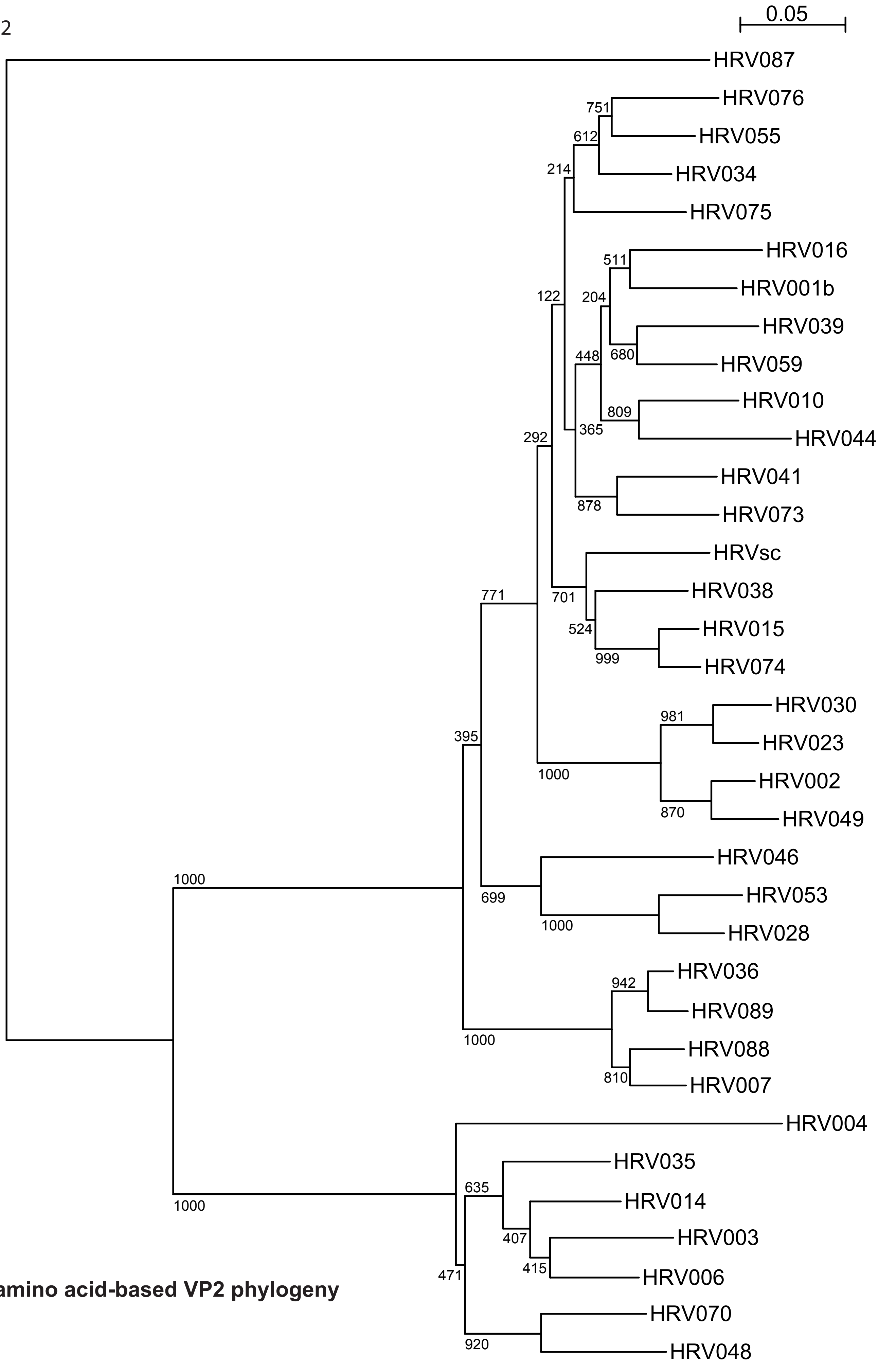

amino acid-based VP2 phylogeny

Data S2

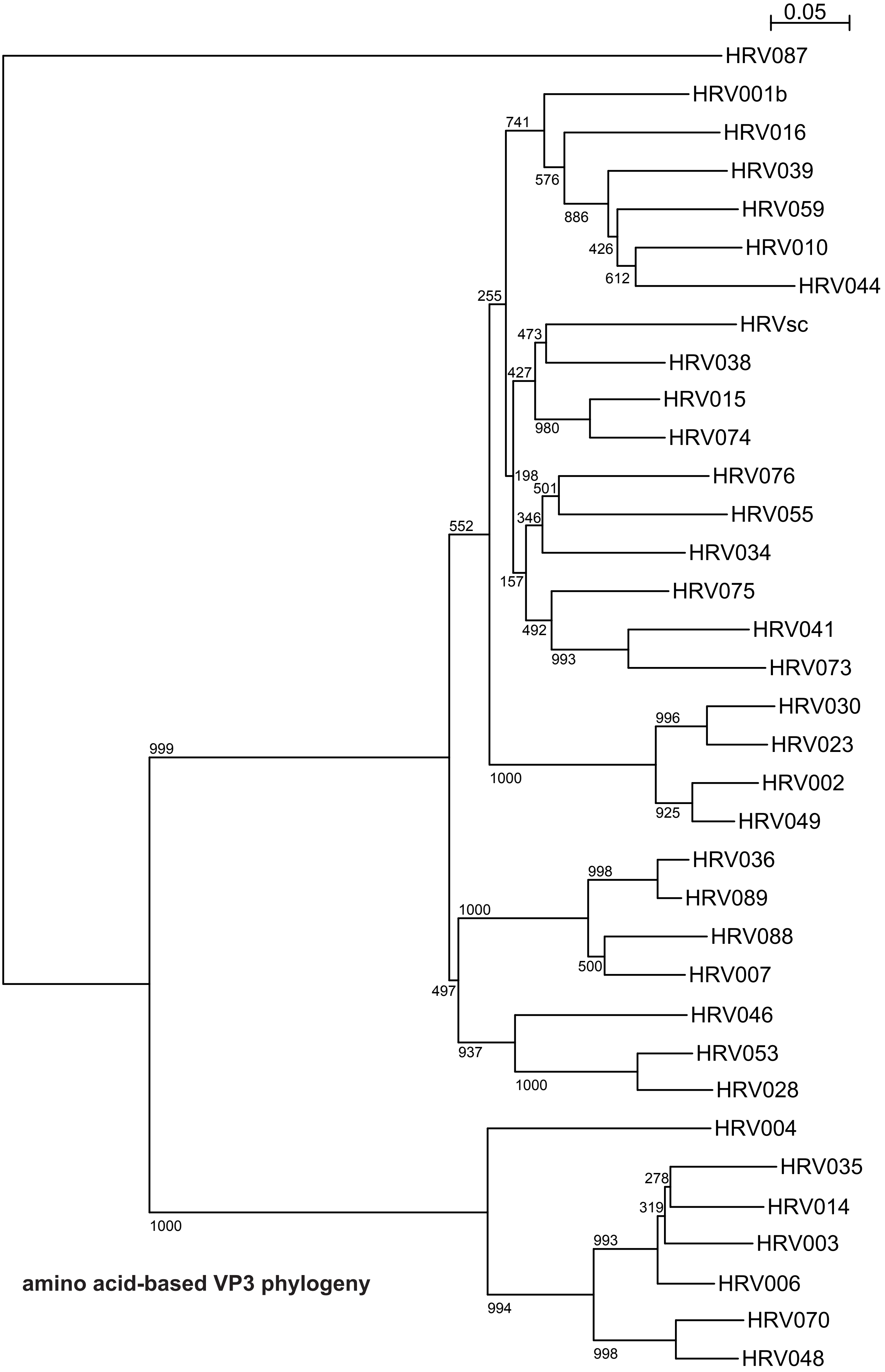

amino acid-based VP3 phylogeny

Data S2

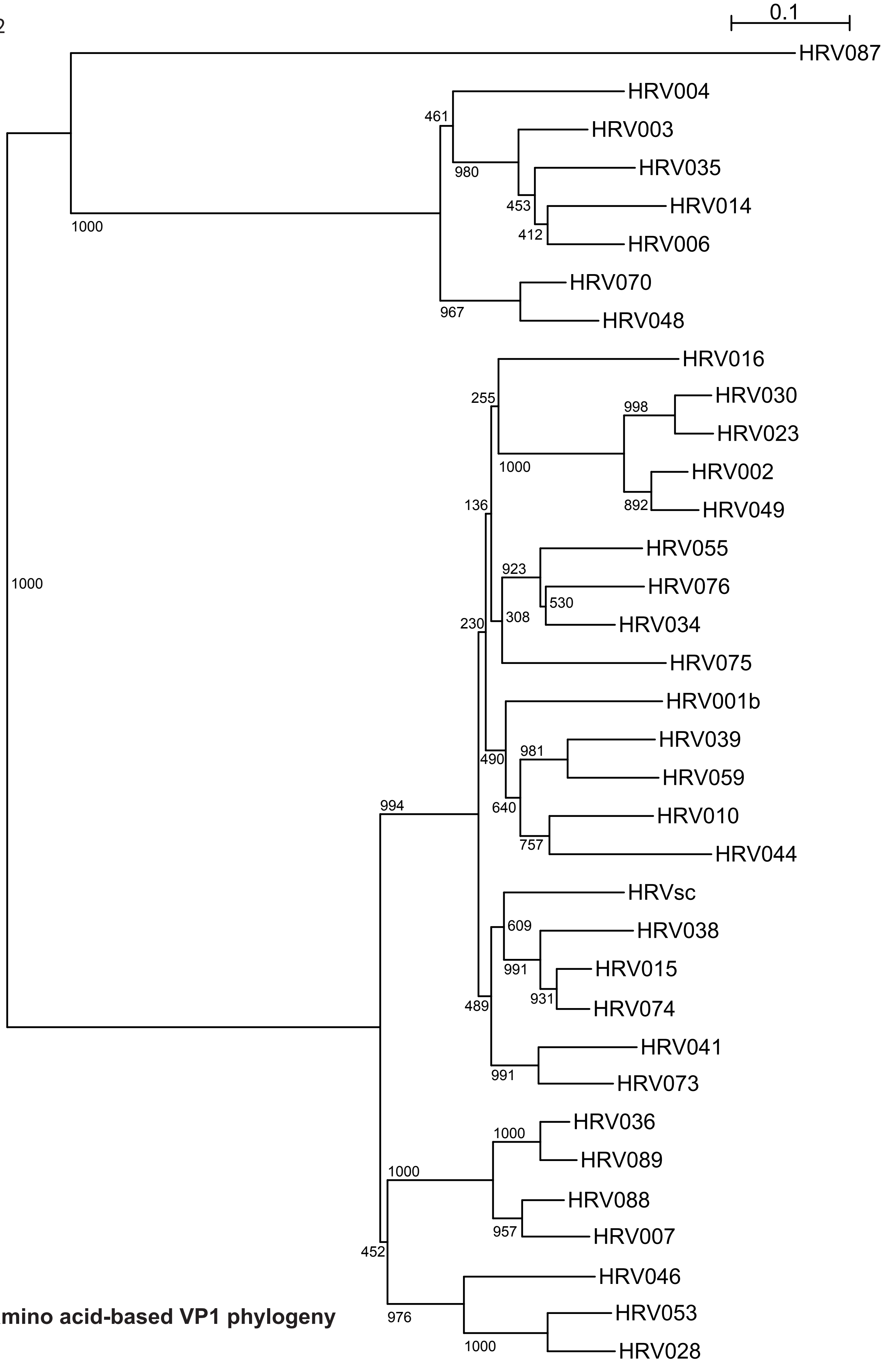

amino acid-based VP1 phylogeny

Data S2

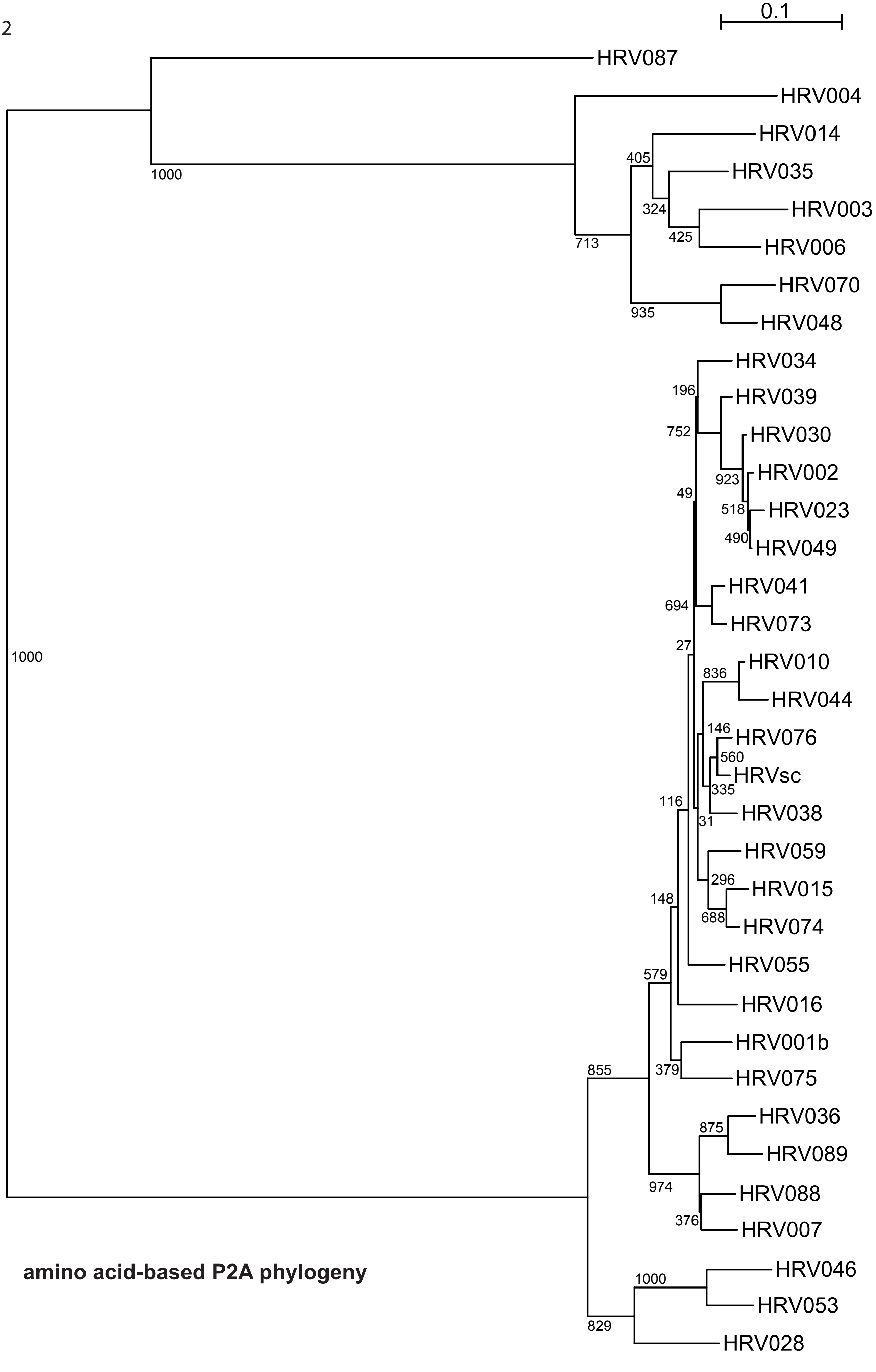

amino acid-based P2A phylogeny

Data S2

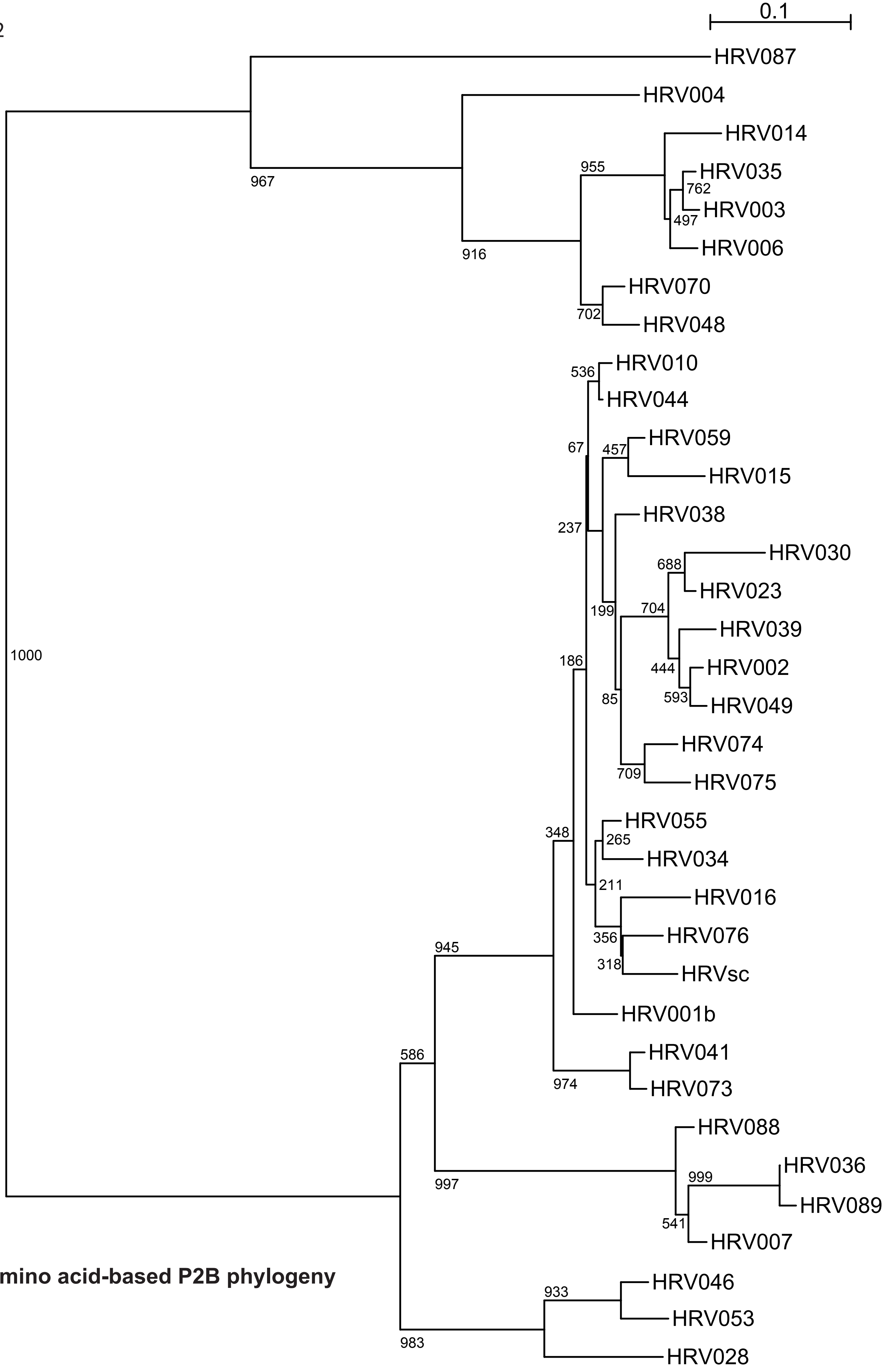

amino acid-based P2B phylogeny

Data S2

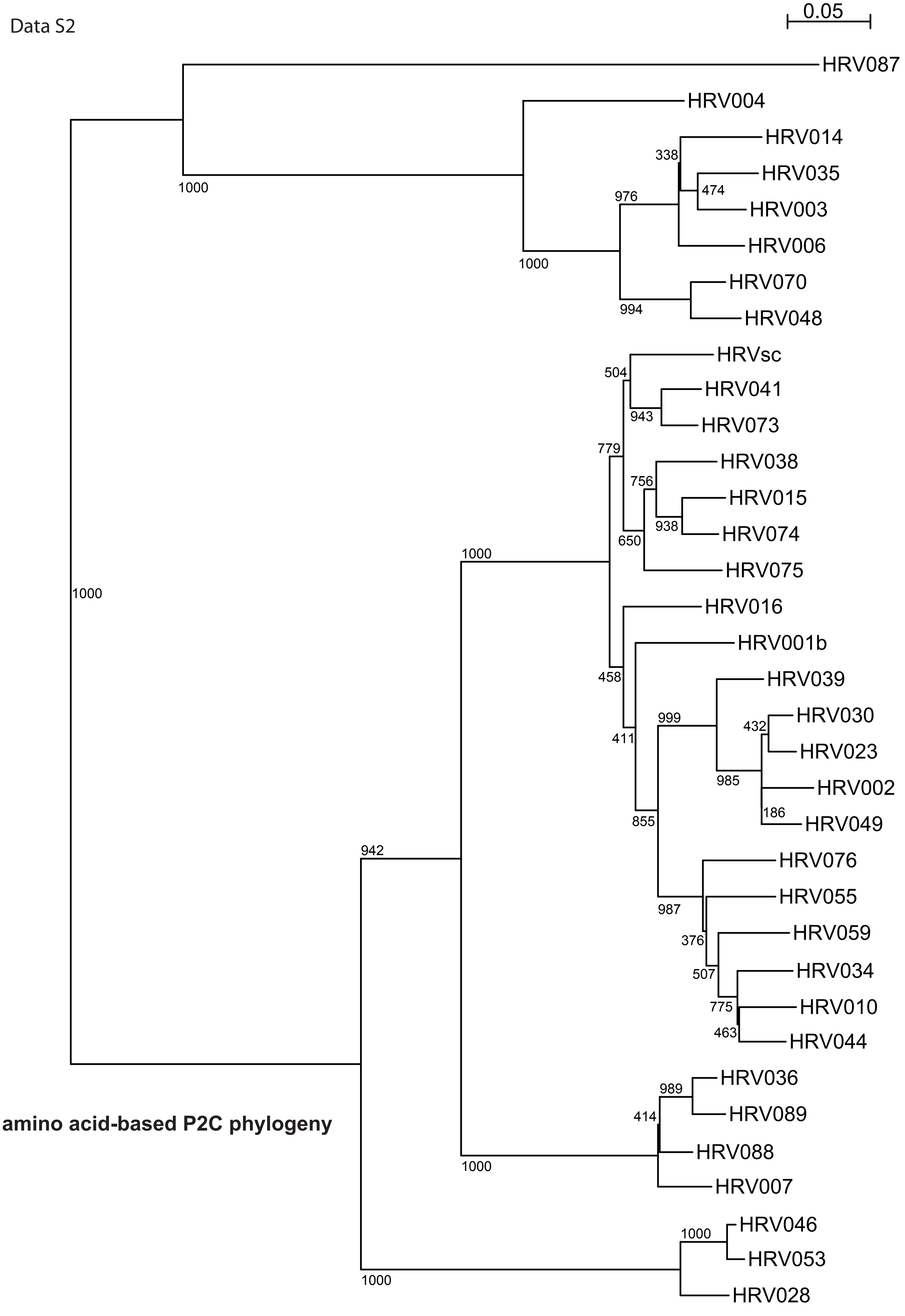

amino acid-based P2C phylogeny

Data S2

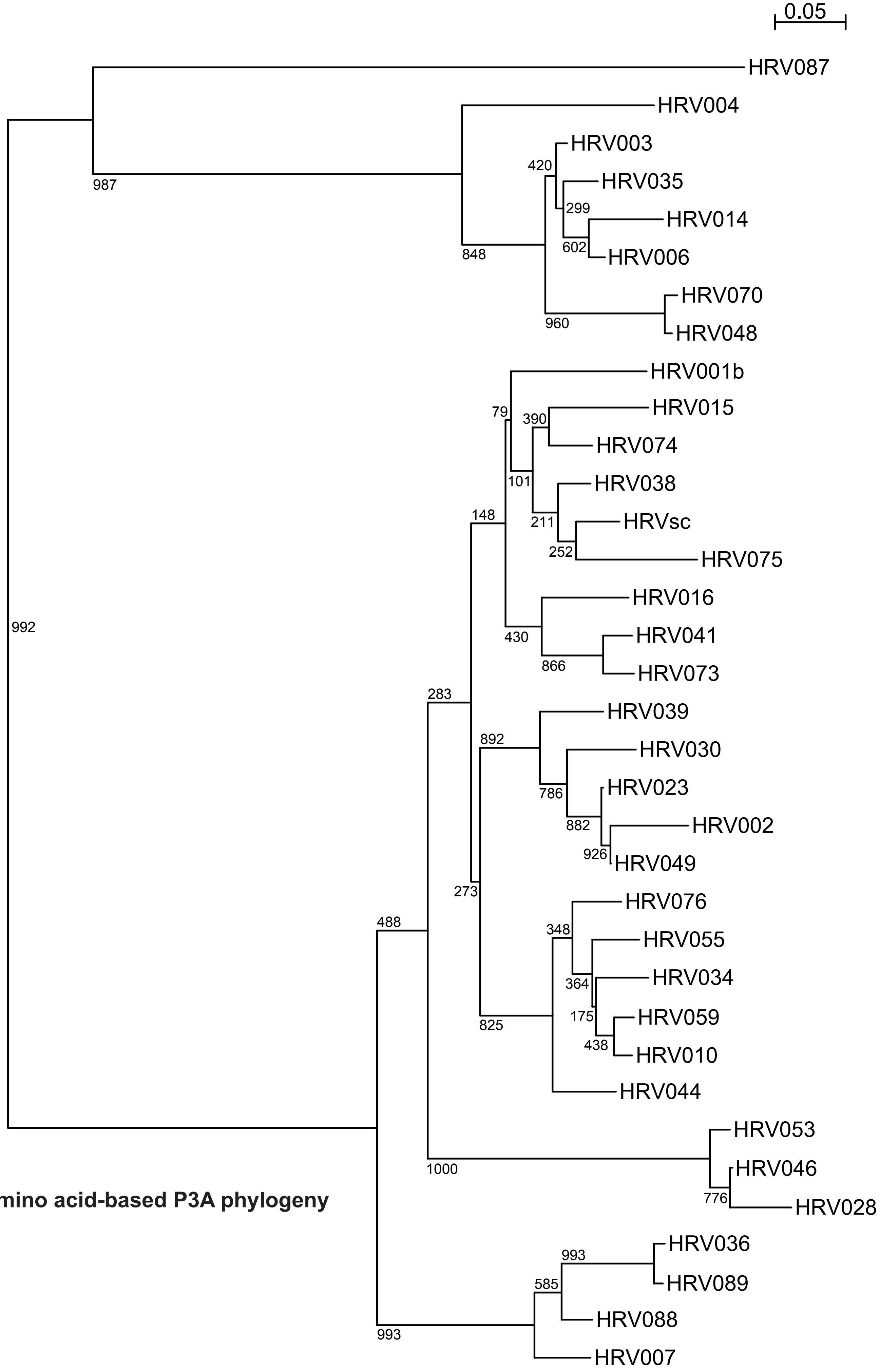

amino acid-based P3A phylogeny

Data S2

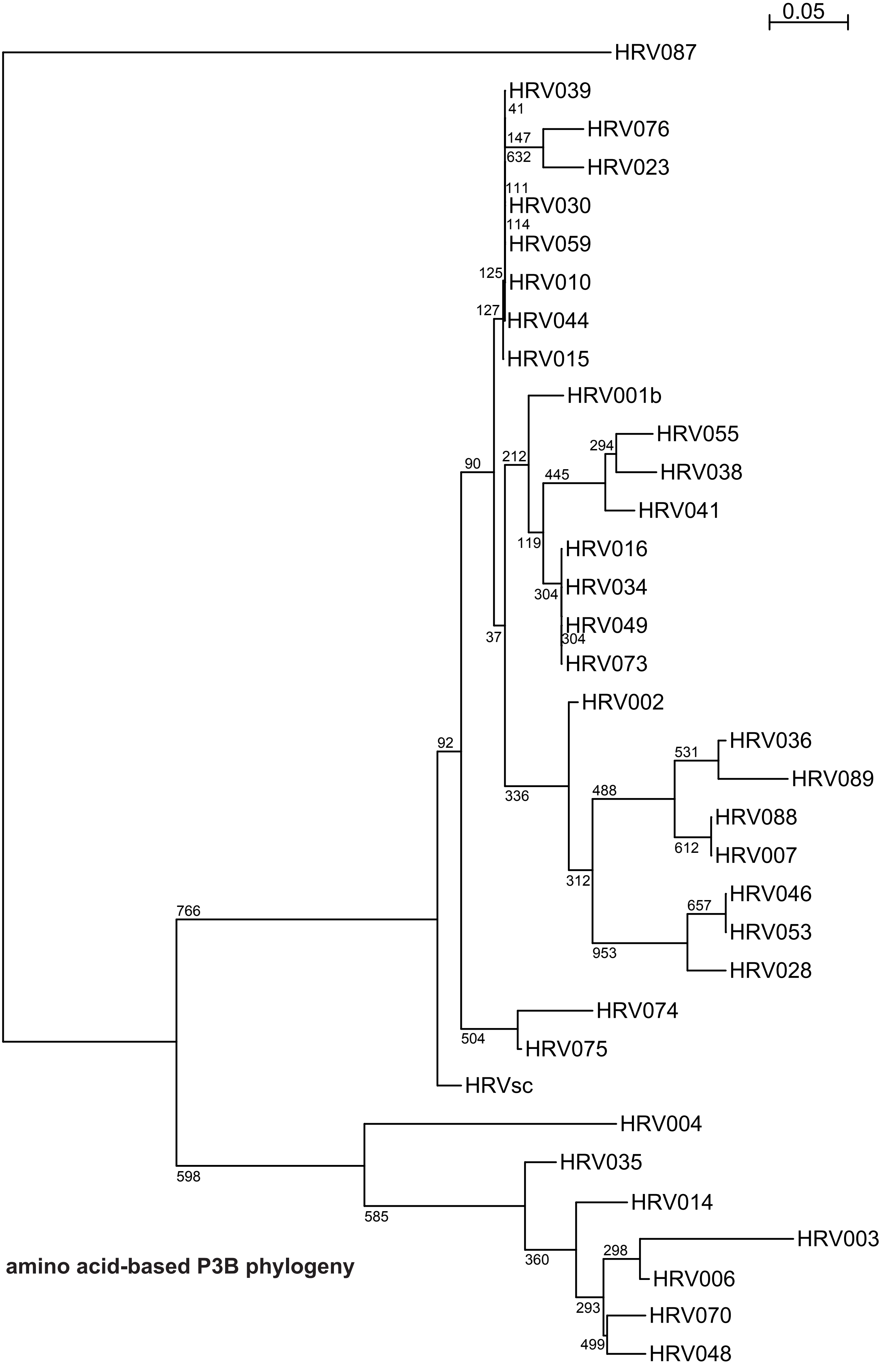

amino acid-based P3B phylogeny

Data S2

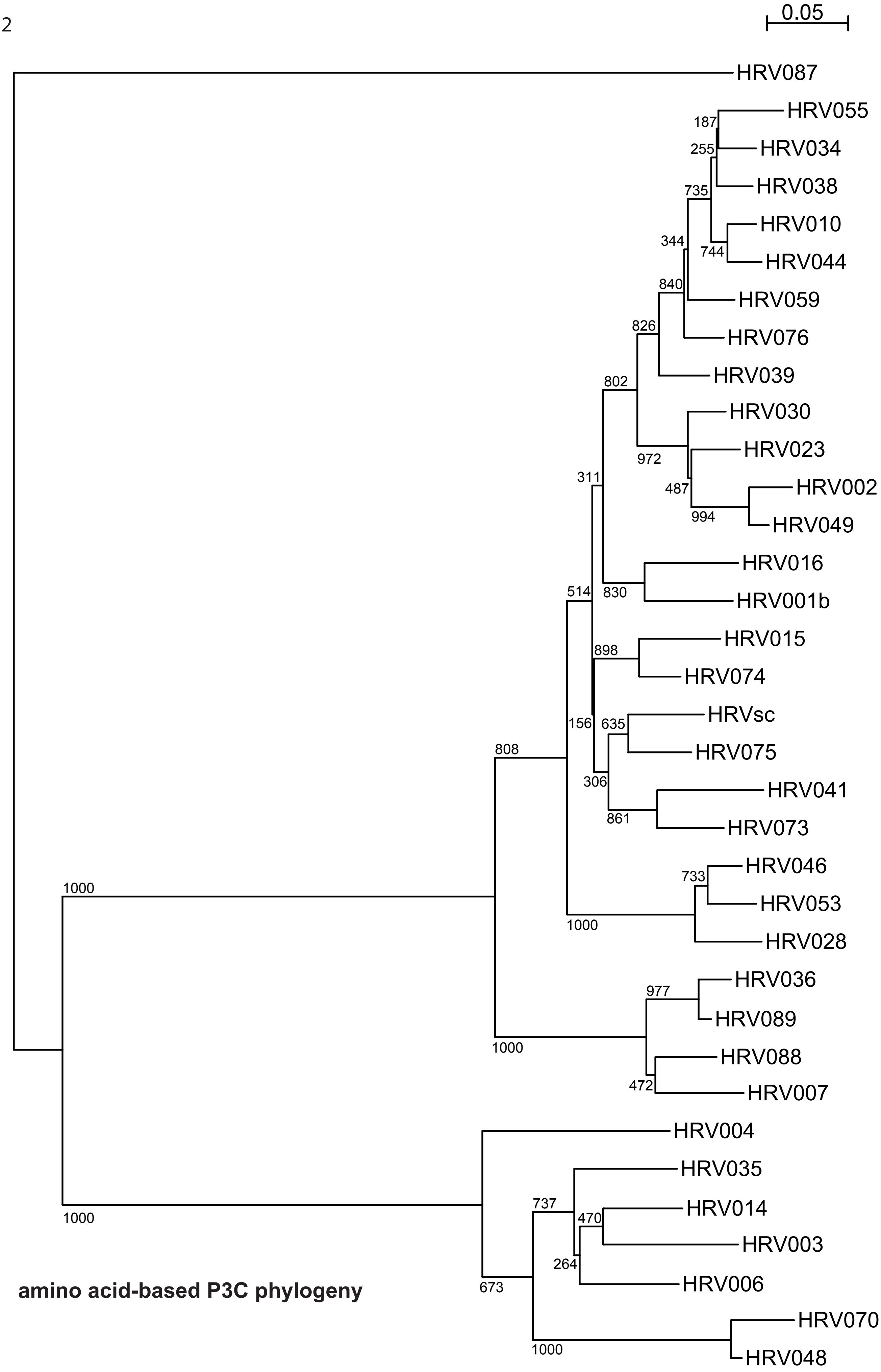

amino acid-based P3C phylogeny

Data S2

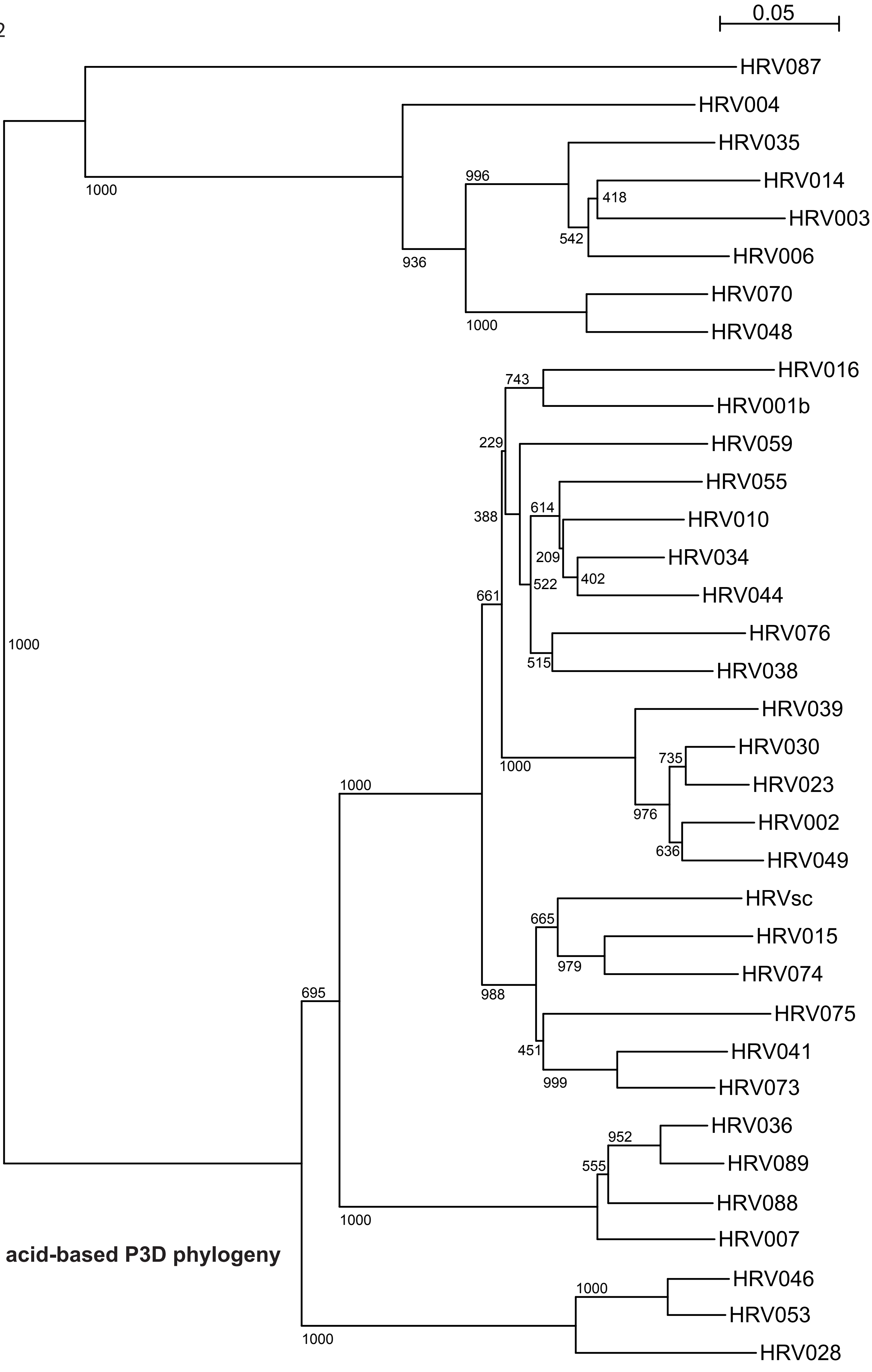

amino acid-based P3D phylogeny
