# Supplementary material for: Genome-wide diversity and selective pressure in the human rhinovirus
Source: Virol J. 2007 May 3;4:40. doi: 10.1186/1743-422X-4-40 (PMC1892812; doi:10.1186/1743-422X-4-40)
Supplement: Additional File 2 — Supplemental Figure 4 and Supplemental Table 1. [file 1743-422X-4-40-S2.pdf]

Figure S4 – Comparison of pairwise nucleotide sequence identity profiles of HRV and HEVs.

Top, Picornavirus genome organization. Genome schematic depicting genes in coding regions (boxes) and the non-coding regions (lines). Black bars above genome schematic indicate classes of gene products and gene product identities, where known VP=viral protein; PRO=viral protease; ATPase=DEXH-box ATPase protein; VPg=viral protein genomic (highlighted by dotted box); POL=RNA dependent RNA polymerase; NCR=non-coding region; coordinates of gene boundaries derived from alignment of available HRV genome sequences; gray shading of every other gene is provided for orientation in lower panels. Boxes below, Average pairwise nucleotide identity scans within HRVA, HRVB, HEVA, HEVB, HEVC, and HEVD genomes in a window of 100 nucleotides, advanced in single nucleotide steps across the genome. (PDF)

Table S1 - Potential recombination events detected among HRV serotypes.

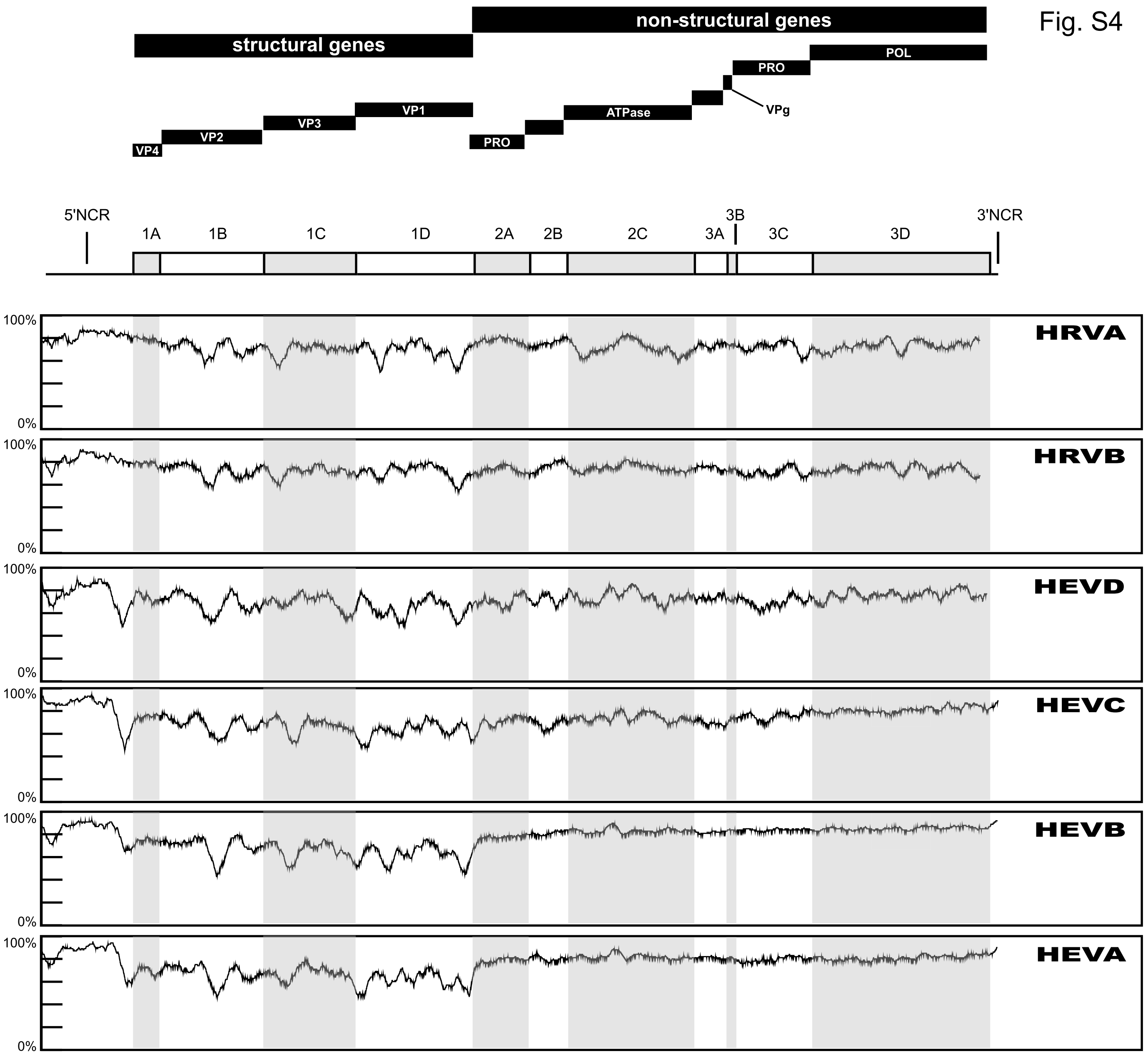
Fig. S4
non-structural genes
structural genes
PRO
POL
VPg
VP1
ATPase
VP3
VP2
PRO
VP4
5'NCR
3B
3'NCR
1A
1B
1C
1D
2A
2B
2C
3A
3C
3D
100%
0%
HRVA
HRVB
HEVD
HEVC
HEVB
HEVA

TABLE S1. Potential recombination events between HRV serotypes identified using six automated recombination detection programs*

Genomic Locus	Major [A]	Potential Minors [B]	Predicted By
212-686	HRV4	HRV3,6,14	RDP, GENECONV, Bootscan
212-686	HRV46	HRV34	RDP, GENECONV, Bootscan, SIScan
212-686	HRV55	HRV44,74,28	RDP, GENECONV
212-686	HRV74/15[C]	HRV30,23,2,49	RDP, GENECONV, Bootscan
212-686	HRV3	HRV6	RDP, GENECONV
1019-1169	HRV3	HRV4	RDP, Bootscan
2127-2387	HRV46	Unknown [D]	Bootscan, SIScan
2127-2387	HRV34	Unknown [D]	Bootscan, SIScan
2292-2525	HRV46	Unknown [D]	GENECONV, SIScan
3185-3269	HRV44	HRVB [E]	RDP, SIScan, MaxChi

*RDP, GENECONV, Bootscan, SiScan, MaxChi, and Chimaera implemented in RDP2 (Materials and Methods).

[A] The serotype in which the crossover event was detected.

[B] In no case was it clear which serotype acted as the donor of the crossover sequence. Listed serotypes had the highest similarity to the crossover sequence.

[C] This recombination event is detected in both HRV74 and HRV15, suggesting that it occurred to their most recent common ancestor.

[D] In some cases, the second sequence donor is obviously missing from the dataset.

[E] Although it is not clear which serotype serves as the donor of the crossover sequence in this event, it is clear that the donor is from the HRVB subgroup.
