# Supplementary material for: Genome-wide diversity and selective pressure in the human rhinovirus
Source: Virol J. 2007 May 3;4:40. doi: 10.1186/1743-422X-4-40 (PMC1892812; doi:10.1186/1743-422X-4-40)
Supplement: Additional File 3 — Supplemental Figures 5–7 and Supplemental Table 2. [file 1743-422X-4-40-S3.pdf]

Figure S5 – Analysis of average minimum distances between diversifying residues in HRVA and HRVB capsids.

(A) Histogram showing the distribution of average minimum distances between two randomly chosen sets of 20% of residues in the HRVA (set 1) and HRVB (set 2) capsids. (B) The observed value of the average minimum distance between the top 5% dN/dS residues in HRVA and HRVB is labeled with a red arrow, with corresponding p-value. Histogram showing the count of top 5% most diversifying dN/dS residues in HRVA (red) and HRVB (blue) from the center of the viral pentamer. For both (A) and (B), distances are three-dimensional Cartesian distances, in angstroms, between α–carbons. (PDF)

Figure S6 – Analysis of average minimum distances between diversifying residues in HRV2 and HRV16 capsids.

Histogram showing the distribution of average minimum distances between a randomly chosen set of 20% of residues in the HRV2 (A, C) and HRV16 (B) capsids. Distances are three-dimensional Cartesian distances, in angstroms, between α –carbons. Observed values are labeled with red arrows, with corresponding p-values noted.(A) Average distances to the nearest residue in the set of naturally occurring neutralizing antibody escape mutants (nIM) in HRV2. (B) Average distances to the nearest residue in the set of residues known to interact with the ICAM cellular receptor in HRV16. (C) Average distances to the nearest residue in the set of residues known to interact with the LDLR cellular receptor in HRV2. (PDF)

Figure S7 – Analysis of overlap between most diversifying capsid residues and viral capsid functional sites.

(A) Top 5% most diversifying residues in the HRV2 capsid pentamer (Verdauger et al., 2000) overlaid onto the characterized HRV antigenic sites (Appleyard et al., 1990; Hastings et al., 1990; Speller et al., 1993; Hewat and Blaas, 1996; Hewat et al., 1998). (B) Top 5% most diversifying residues in the HRV16 capsid pentamer (Hadfield, et al., 1997) overlaid onto the characterized ICAM1 cellular receptor contacts (Bella et al., 1999). (C) Top 5% most diversifying residues in the HRV2 capsid pentamer (Verdauger et al., 2000) overlaid onto the characterized LDLR cellular receptor contacts (Verdauger et al., 2004). Diversifying residues are shown in red, shaded according to corresponding dN/dS values as indicated by the scale bar below panel (C); green, antigenic residues (A); ICAM1 receptor contacts (B), and LDLR contacts (C); yellow, diversifying residues that directly overlap functional residues. Inset histogram, distribution of minimal distances between α-carbons of diversifying residues and antigenic sites (A), ICAM1 contact residues (B), and LDLR contact residues (C); Y-axis is simple frequency count, with a range that varies for each panel; p values provide frequency at which an average minimum distance similar to that for the observed distribution was detected when the locations of the diversifying residues were randomized on each pentamer surface, and minimal distances to antigenic site residues (A), ICAM1R contact residues (B), and LDLR contact residues (C) were measured (n=100,000 randomizations). Histograms at right, (A) average distances to the nearest residue in the set of naturally occurring neutralizing antibody escape mutants (nIM) in HRV2; (B) Average distances to the nearest residue in the set of residues known to interact with the ICAM cellular receptor in

HRV16; (C) Average distances to the nearest residue in the set of residues known to interact with the LDLR cellular receptor in HRV2. Observed values are labeled with red arrows, with corresponding p-values noted. (PDF)

Table S2 - Selective pressure in pleconaril contacts.

A

Distribution of Average Minimum Distance between two randomly chosen sets of 20% of residues on the HRVA (set1) and HRVB (set2) capsids.

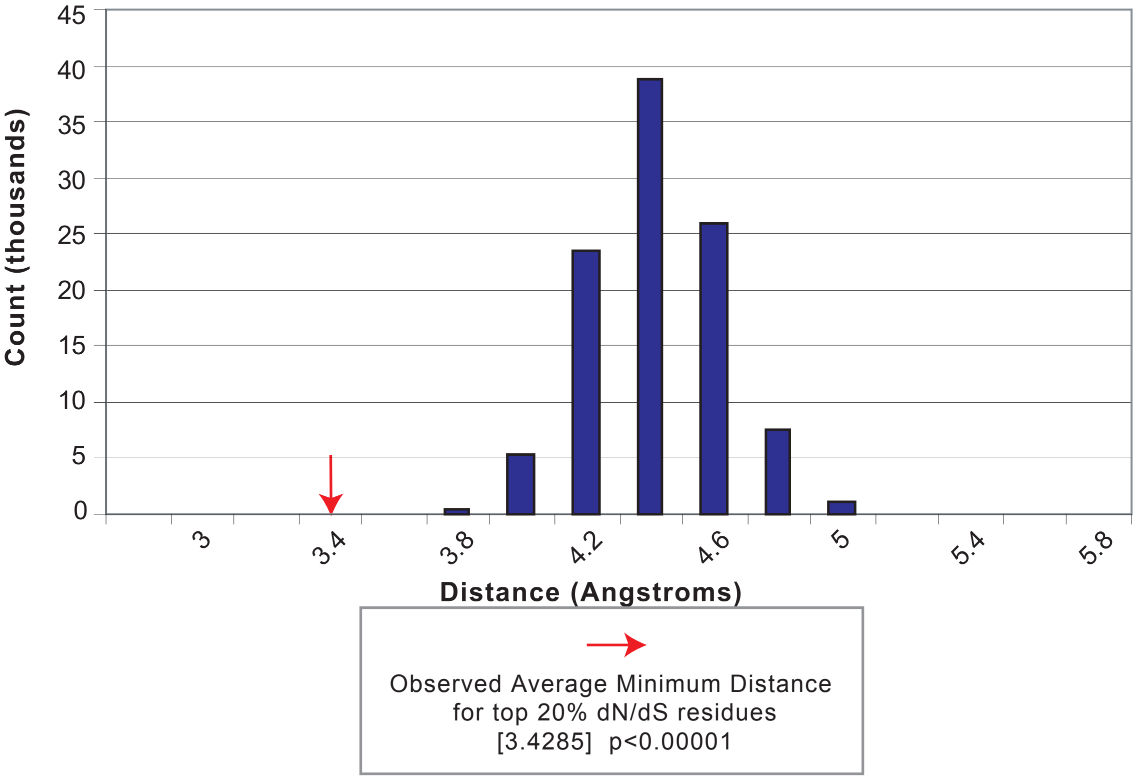

B

Distribution of Average Minimum Distance between two randomly chosen sets of 5% of residues on the HRVA (set1) and HRVB (set2) capsids.

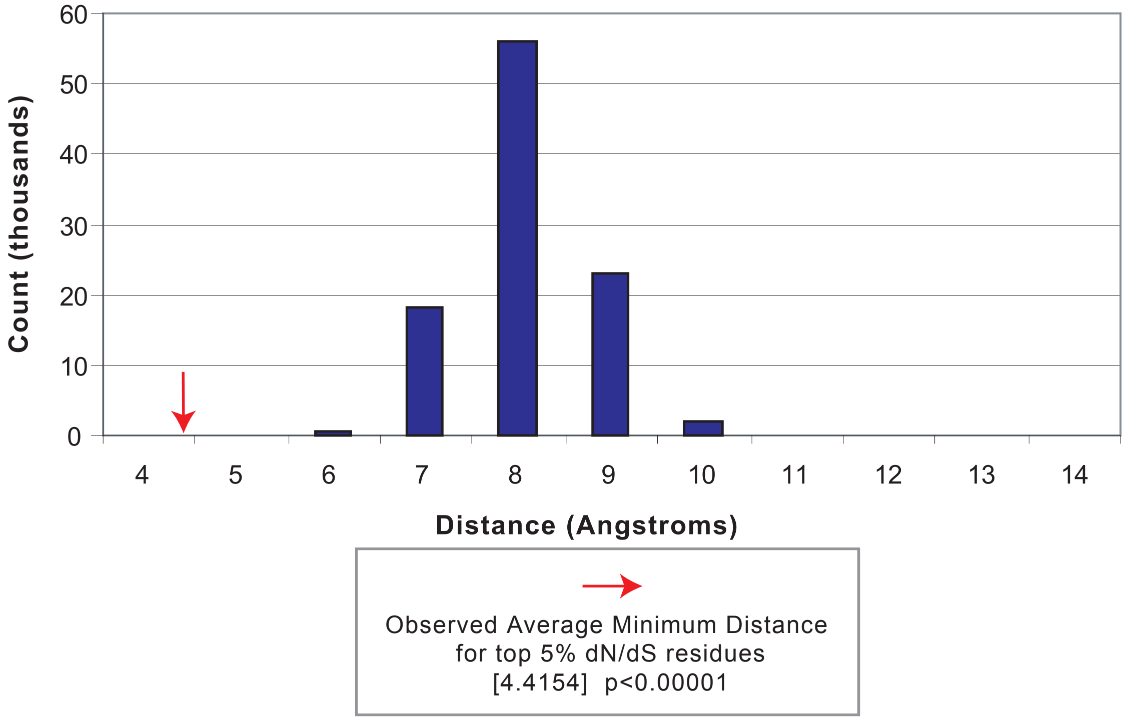

Fig. S6

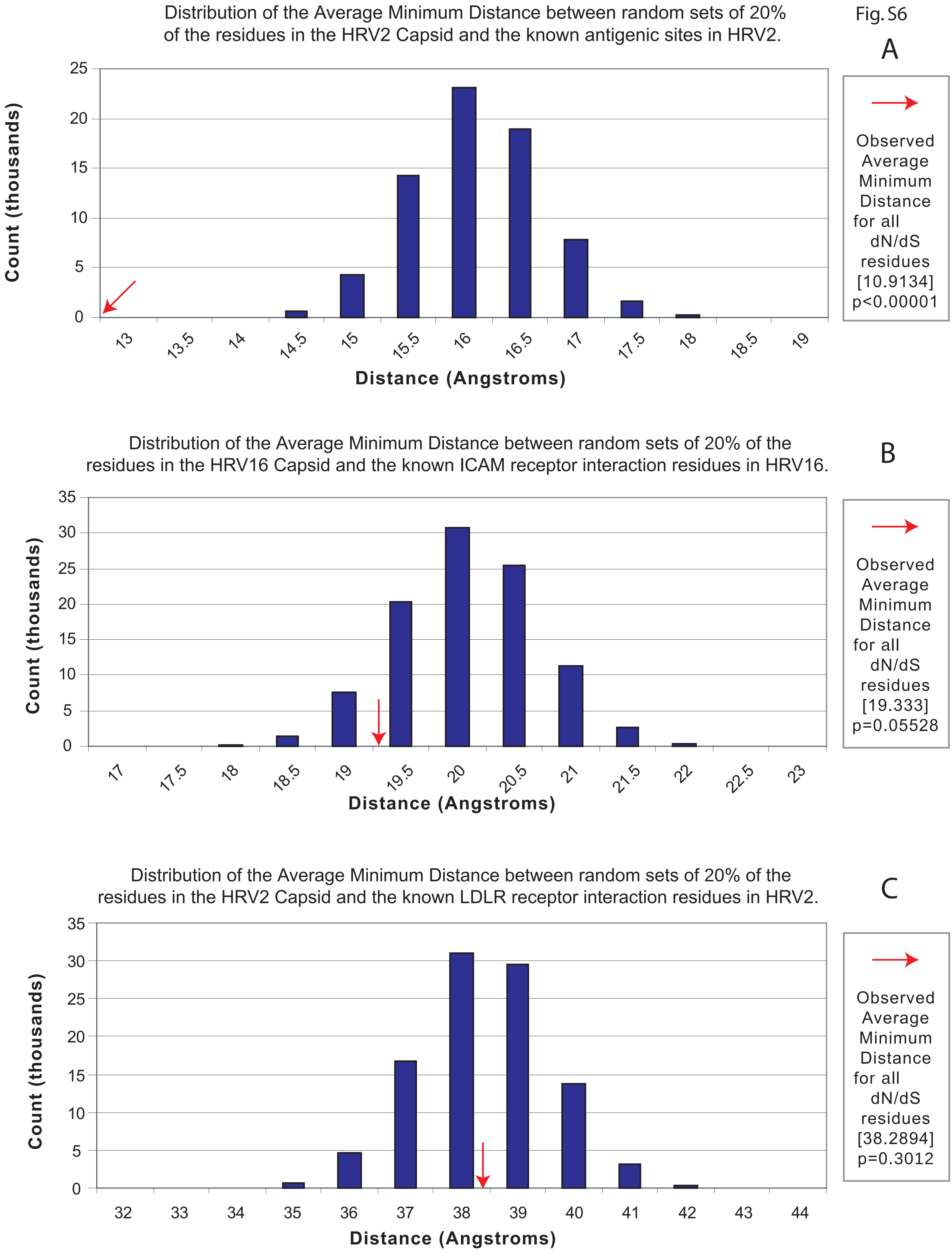
A
Distribution of the Average Minimum Distance between random sets of 20% of the residues in the HRV2 Capsid and the known antigenic sites in HRV2.
Count (thousands)
Distance (Angstroms)
Observed Average Minimum Distance for all dN/dS residues [10.9134] p<0.00001
B
Distribution of the Average Minimum Distance between random sets of 20% of the residues in the HRV16 Capsid and the known ICAM receptor interaction residues in HRV16.
Count (thousands)
Distance (Angstroms)
Observed Average Minimum Distance for all dN/dS residues [19.333] p=0.05528
C
Distribution of the Average Minimum Distance between random sets of 20% of the residues in the HRV2 Capsid and the known LDLR receptor interaction residues in HRV2.
Count (thousands)
Distance (Angstroms)
Observed Average Minimum Distance for all dN/dS residues [38.2894] p=0.3012

Fig. S7

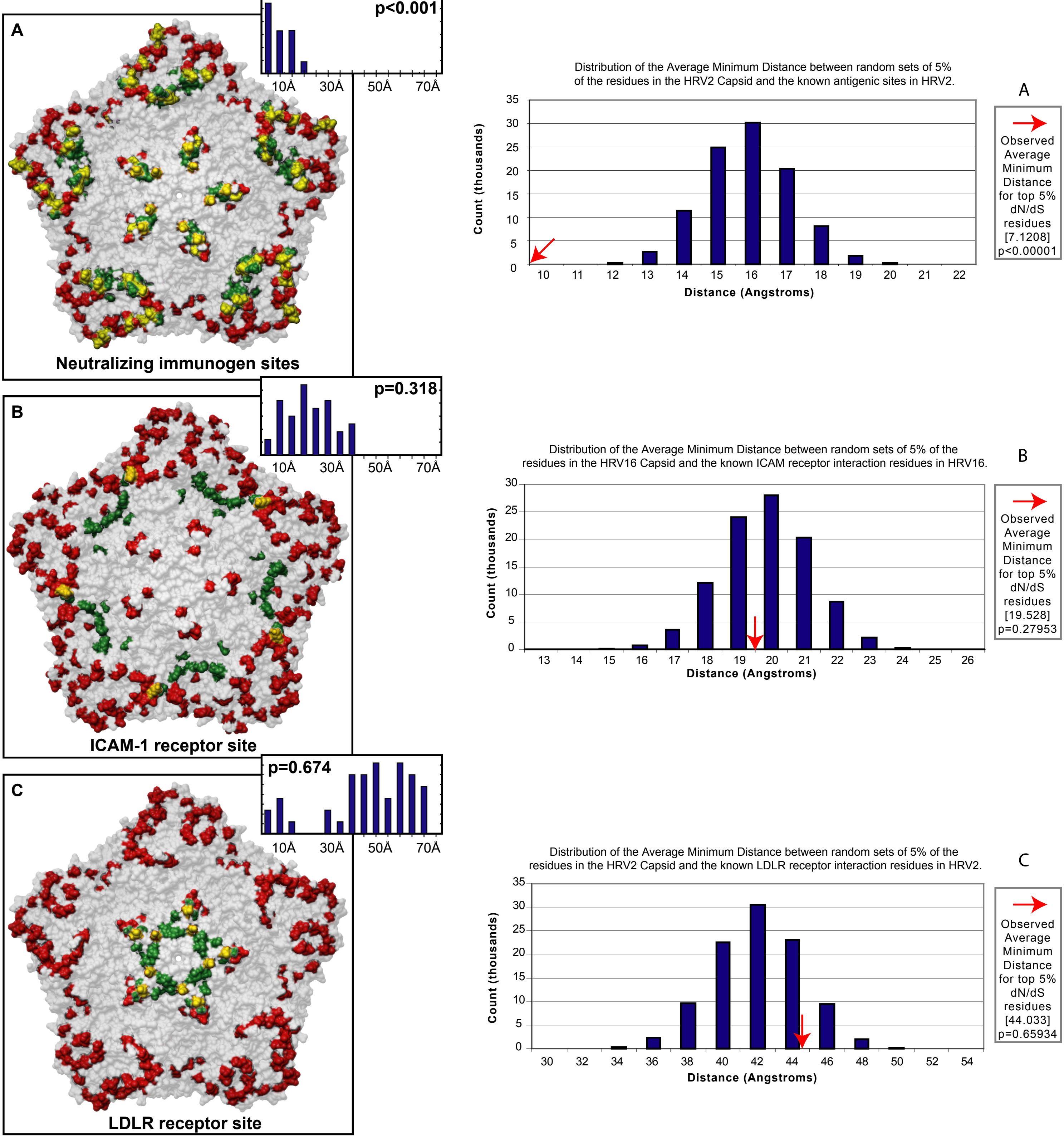

α-carbon minimum distances
A
p<0.001
10Å
30Å
50Å
70Å
Neutralizing immunogen sites
B
p=0.318
ICAM-1 receptor site
C
p=0.674
LDLR receptor site
Distribution of the Average Minimum Distance between random sets of 5% of the residues in the HRV2 Capsid and the known antigenic sites in HRV2.
Count (thousands)
Distance (Angstroms)
Observed Average Minimum Distance for top 5% dN/dS residues [7.1208] p<0.00001
Distribution of the Average Minimum Distance between random sets of 5% of the residues in the HRV16 Capsid and the known ICAM receptor interaction residues in HRV16.
Observed Average Minimum Distance for top 5% dN/dS residues [19.528] p=0.27953
Distribution of the Average Minimum Distance between random sets of 5% of the residues in the HRV2 Capsid and the known LDLR receptor interaction residues in HRV2.
Observed Average Minimum Distance for top 5% dN/dS residues [44.033] p=0.65934

Table S2: Selective pressure in pleconaril contacts[a]

Residue number[b]	dN/dS value
1104	0.05
1106	0.06
1128	0.05
1150	0.05
1151	0.05
1152	0.05
1174	0.05
1175	0.05
1176	0.05
1186	0.05
1188	0.05
1191	0.12
1197	0.05
1219	0.05
1221	0.05
1224	0.06
3024	0.05
1098	0.05
1100	0.05
1122	0.05
1124	0.05
1142	0.05
1143	0.05
1144	0.05
1166	0.05
1167	0.05
1168	0.05
1179	0.05
1181	0.05
1184	0.05
1190	0.05
1212	0.05
1214	0.05
1217	0.05
1104	0.05

[a]HRV14 contacts with pleconaril, a capsid 'pocket' binding drug from Zhang et al., 2004 J.Vir 78, 11061-9. [b]First digit in residue number designates the viral capsid protein (here 1=VP1) and subsequent numbers indicate residue within designated viral protein relative to N-terminal proteolytic cleavage site. Residues highlighted in yellow are two residues in the pleconaril binding pocket of VP1 that are consistently different between susceptible and resistant HRVB serotypes (Ledford et al., 2005. Antiviral Research 68, 135-138).
