# Supplementary material for: Genome-wide diversity and selective pressure in the human rhinovirus
Source: Virol J. 2007 May 3;4:40. doi: 10.1186/1743-422X-4-40 (PMC1892812; doi:10.1186/1743-422X-4-40)
Supplement: Additional File 4 — Supplemental Table 3, Supplemental Data S3 and S4, and Supplemental Figure 8. [file 1743-422X-4-40-S4.pdf]

Table S3 - Selective pressure in ruprintrivir contacts.

Data S3 – Consensus structures HRVA and HRVB 5’NCR elements R1-R7.

Consensus secondary structure and sequence of HRVA and HRVB 5’NCR elements identified in Witwer, C et al., 2001). Circled residues, positions where compensatory substitutions are detected in the alignment. Gray residues indicate positions where substitutions that disrupt basepairing potential are detected in the alignment. (PDF)

Data S4 – Consensus structures HRVA and HRVB 3’NCR element.

Consensus secondary structure and sequence of HRVA and HRVB 3’NCR element identified in Witwer, C et al., 2001). Circled residues, positions where compensatory substitutions are detected in the alignment. Gray residues indicate positions where substitutions that disrupt basepairing potential are detected in the alignment. (PDF)

Figure S8 – Analysis of HRVA and HRVB cis-replicating elements (CREs).

ClustalW alignment of HRVA (A) and HRVB (B) region of the P2A gene (HRV002 nucleotides 3268 – 3302) in which the minimal functional CRE has been identified for a member of the HRVA subgroup (Gerber, K. et al., 2001). ClustalX alignment of HRVA (A) and HRVB (B) region of the VP1 gene (HRV014 nucleotides 2353 to 2386) in which the minimal functional CRE has been identified for a member of the HRVB subgroup (McKnight, KL and Lemon, SM, 1998; Yang, Y et al., 2002). Shorthand for consensus secondary structures deduced from each of these alignments (Hofacker, I et al., 2004) are depicted above with parentheses above the clustalx alignment indicate base-paired nucleotides, while dots or commas indicate unpaired nucleotides.. (PDF)

Table S3: Selective pressure in ruprintrivir contacts[a]

Residue number[b]	dN/dS value
25	0.05
40	0.05
71	0.05
125	0.05
127	0.05
128	0.05
130	0.05
142	0.05
147	0.05
161	0.05
163	0.05
164	0.05
165	0.05

[a]HRV2 contacts with ruprintrivir, a protease inhibitor targeted to the 3C protease active site (Binford, SL et al., 2005. Antimicrobial Agents Chemother. 49, 619-26). [b]Residue number indicates residue within the 3C protease relative to N-terminal proteolytic cleavage site.

A. R1 cloverleaf element

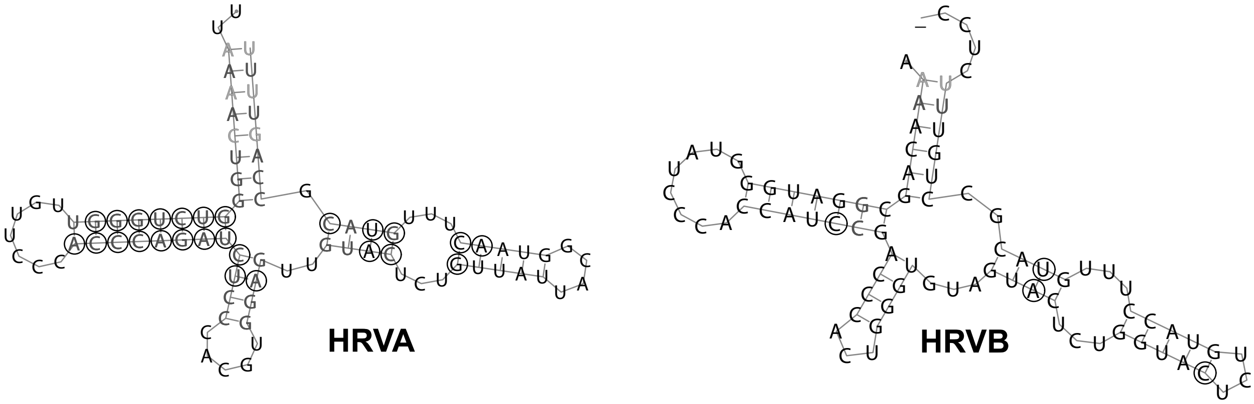

B. R2 element

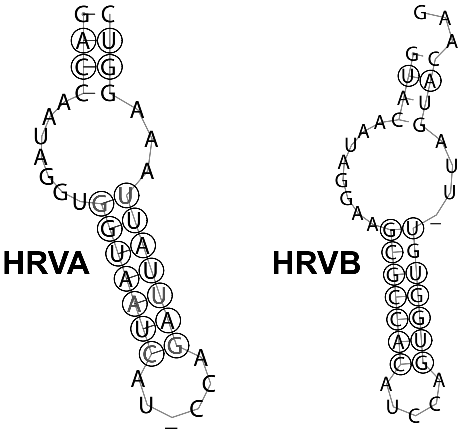

C. R3 element

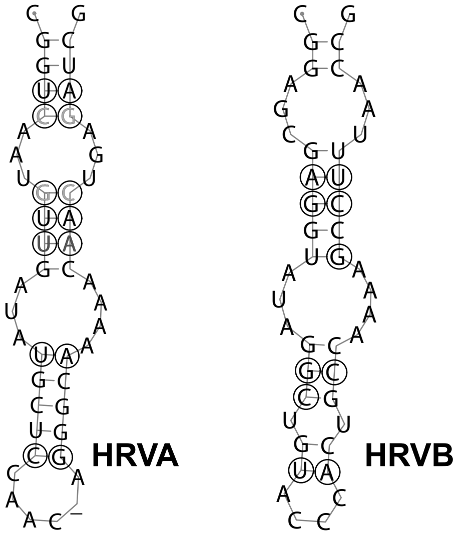

D. R4 element

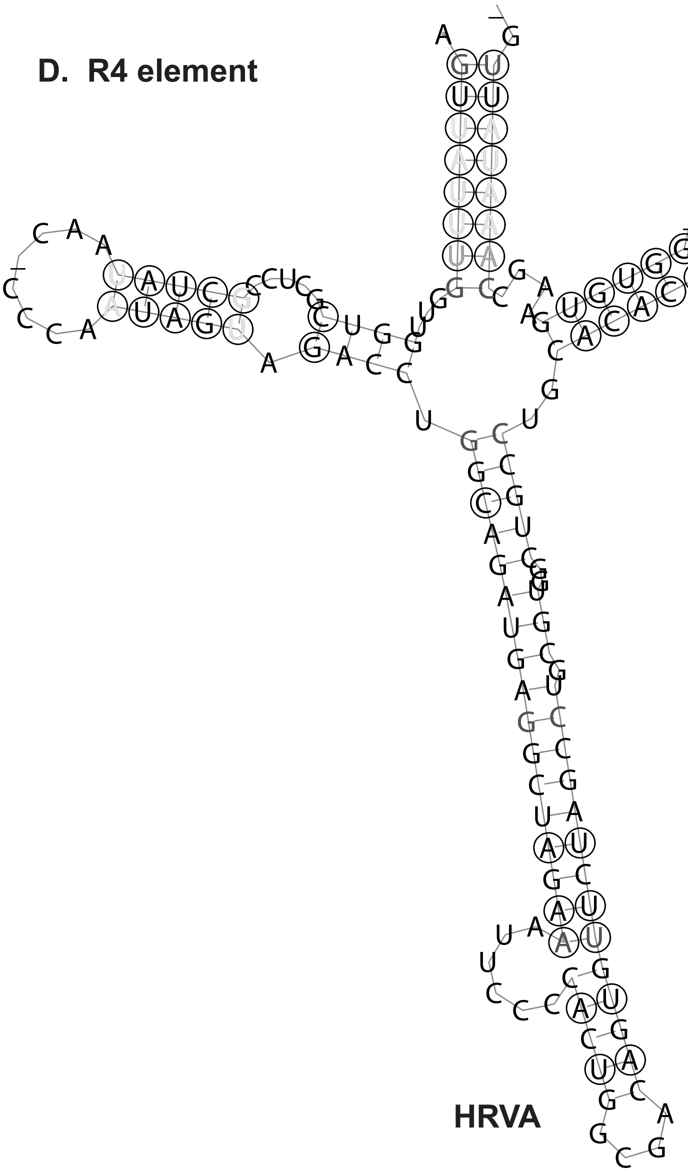

HRVA

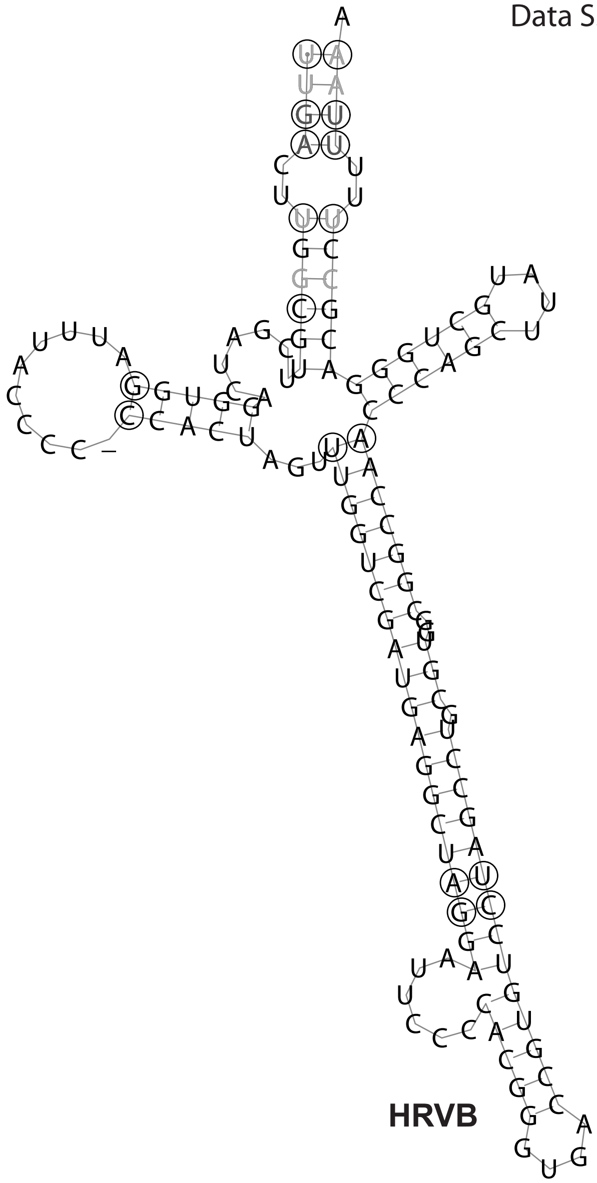

HRVB

E. R5 element

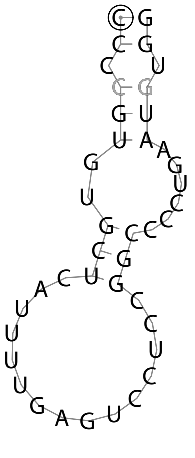

HRVA

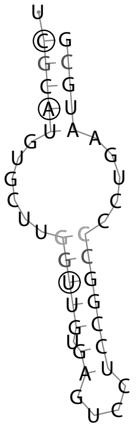

HRVB

F. R6 element

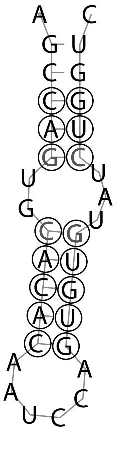

HRVA

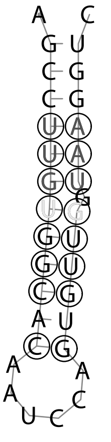

HRVB

G. R7 element

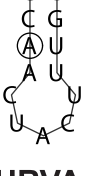

HRVA

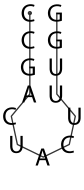

HRVB

3'NCR stem loop element

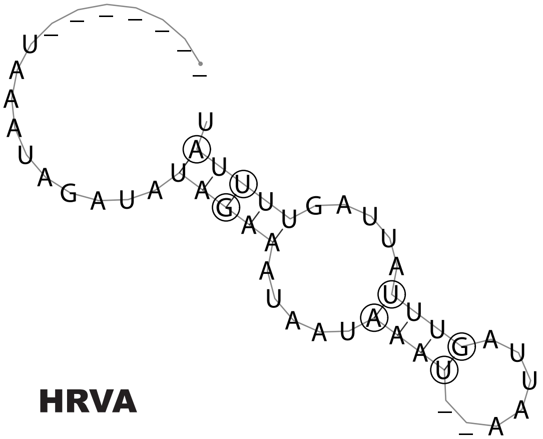

HRVA

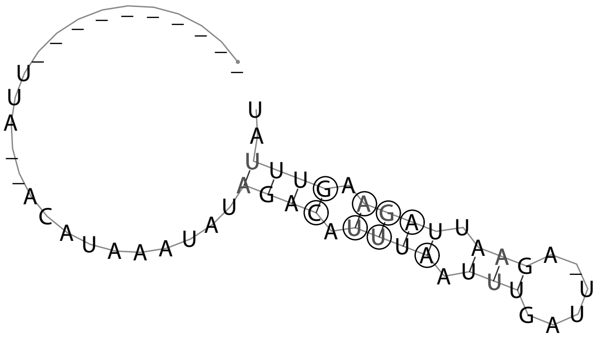

HRVB

Fig. S8AB

A

```
            .((((...(((..............)))...))).
HRV075_     AATCATTTACCGAACAAACACAAAAGGTGATGATT
HRV088_     AATCATTTACCGAACAAACACAAAAGGCGATGATG
HRV030_     AATCATTTACCGAACAAACACTGTAGGTGATGATT
HRV002_     AATCATTTACCGAACAAACACTGTAGGTGATGATT
HRV023_     AATCATTTACCGAACAAACACTATAGGTGATGATT
HRV049_     AATCATTTACCGAACAAACACCATAGGTGATGATT
HRV-Hanks_  AGTCATTTACCGAACAAACACCACAGGAGATGACT
HRV060_     AGTCATTTACCGAACAAACACCACAGGTGATGACT
HRV021_     AGTCATCTACCGAACAAACACCACAGGTGATGACT
HRV082_     AGTCATCTACCGAACAAACACCACAGGTGATGACT
HRV038_     AGTCATTTACCGAACAAACACAACAGGTGATGACT
HRV062_     AGTCATTTACCGAACAAATACAACAGGTGATGACT
HRV031_     AGTCATTTACCGCACAAACACTACAGGCAATGACT
HRV066_     AGTCATTTACCGCACAAATACTACAGGCAATGACT
HRV047_     AGTCATCTACCGCACAAACACTACAGGCAATGACT
HRV056_     AGTCATTTACCGCACAAACACTACAGGTGATGACT
HRV044_     AGTCATTTATCGAACAAACACCACAGGTAATGACT
HRV029_     AGTCATTTATCGAACAAACACCACAGGTAATGACT
HRV025_     AGTCATTTACCGAACAAACACAACAGGTAATGACT
HRV100_     AGTCATTTACCGAACAAACACTACAGGTAATGACT
HRV010_     AGTCATTTACCGAACAAACACTATAGGTAATGACT
HRV020_     TGTCATTTACCGAACAAATACAACAGGTGATGACA
HRV068_     TGTCATCTACCGAACAAATACAACAGGTGATGACA
HRV046_     TGTCATTTACCGCACAAATACTACAGGTGATGACA
HRV053_     TGTCATTTACCGCACAAATACTACAGGTGATGACA
HRV028_     TGTCATTTACCGAACAAACACGACAGGTAATGATG
HRV057_     AGTTATCTACCGAACAAATACTACAGGTGATGACT
HRV022_     AGTCATCTACCGAACAAACACTACAGGTGATGATT
HRV013_     AGTCATCTACCGAACAAACACTATAGGTGATGACT
HRV041_     AGTCATCTACCGAACAAACACTATAGGTGATGACT
HRV015_     AGTCATTTACCGAACAAACACTAAAGGTGATGACT
HRV073_     AGTCATTTACCGAACAAACACTATAGGTGATGACT
HRV033_     AGTCATATACCGAACAAACACTACAGGTGATGACT
HRV076_     AGTCATATACCGAACAAACACTACAGGTGATGACT
HRV018_     AGTCATATACCGAACAAACACAACAGGTGATGACT
HRV074_     AGTCATATACCGAACAAACACCAAAGGTGATGACT
HRV039_     AGTCATATACCGAACAAACACCCAAGGTGATGACT
HRV071_     AGTCATATACCGAACAAACACGGAAGGTGATGACT
```

B

```
            ...................,,..............
HRV006_     GGCAGTTGTGTTTACTGGAGCACATGGCGCAGAGA
HRV079_     AGCCATAGTGTTTACAGGTGCACATGGGGCTGAGA
HRV072_     AGCAATTGTTTCAACAGGAGCACATGGTGCAGAGA
HRV014_     AGCAATAGTCTCAACAGGAGGACATGGTGCAGAAA
HRV037_     AGCAATTGTATACACAGGTGCACATGGTGCGGAAA
HRV035_     AGCCATTGTAGCAACAGGTGCTCATGGTGCAGAAA
HRV092_     AGCAGTAGTATCAACTGGTGCTCATGGAGCAGAGA
HRV003_     AGCAGTAGTTGCTACTGGAGCACATGGAGCAGAAA
HRV070_     AGCAGTAGTAGCAACAGGAGCACAGGGCTCAGAAA
HRV052_     AGCAGTTGTAGCAACCGGAGCACAGGGCTCAGAAA
HRV017_     GGCAGTAGTGGCAACAGGAGCGCTGGGTTCAGAAA
HRV048_     GGCAGTTGTGGCAACAGGAGCGCAAGGCTCAGAAA
HRV091_     GGCAGTGGTAGCAACAGGTGCACAAGGCTCAGAAA
HRV069_     GGCAGTGGTAGCAACGGGGGCACAAGGGTCAGAGA
HRV083_     GGCCGTGGTGTCAACAGGTGCTCATGGTGCTGAAA
HRV042_     TGCCATCGTGAGCACGGGTGCTCACGGTTCAGAGA
HRV093_     AGCAGTGGTTAACATTAATGCCCATGGATCAGAGA
HRV097_     GGCAGTAGTCAACATAAATGCTCATGGTTCGGAGA
HRV027_     AGCAGTAATTAGTATAAATGCTCATGGTTCAGAAA
HRV084_     AGCAATAATCAACCTAAATGGGCATGGTTCAGAAA
HRV005_     AGCCATTATCAACATAGGTGCTCATGGCTCAGAAA
HRV026_     GGCTGTAGTTGGGGTGGGTGCCCATGGTTCAGAAA
HRV004_     GGCAATAGTTGATGCAAGGGCTCACGGTTCTGAAA
```

Fig. S8CD

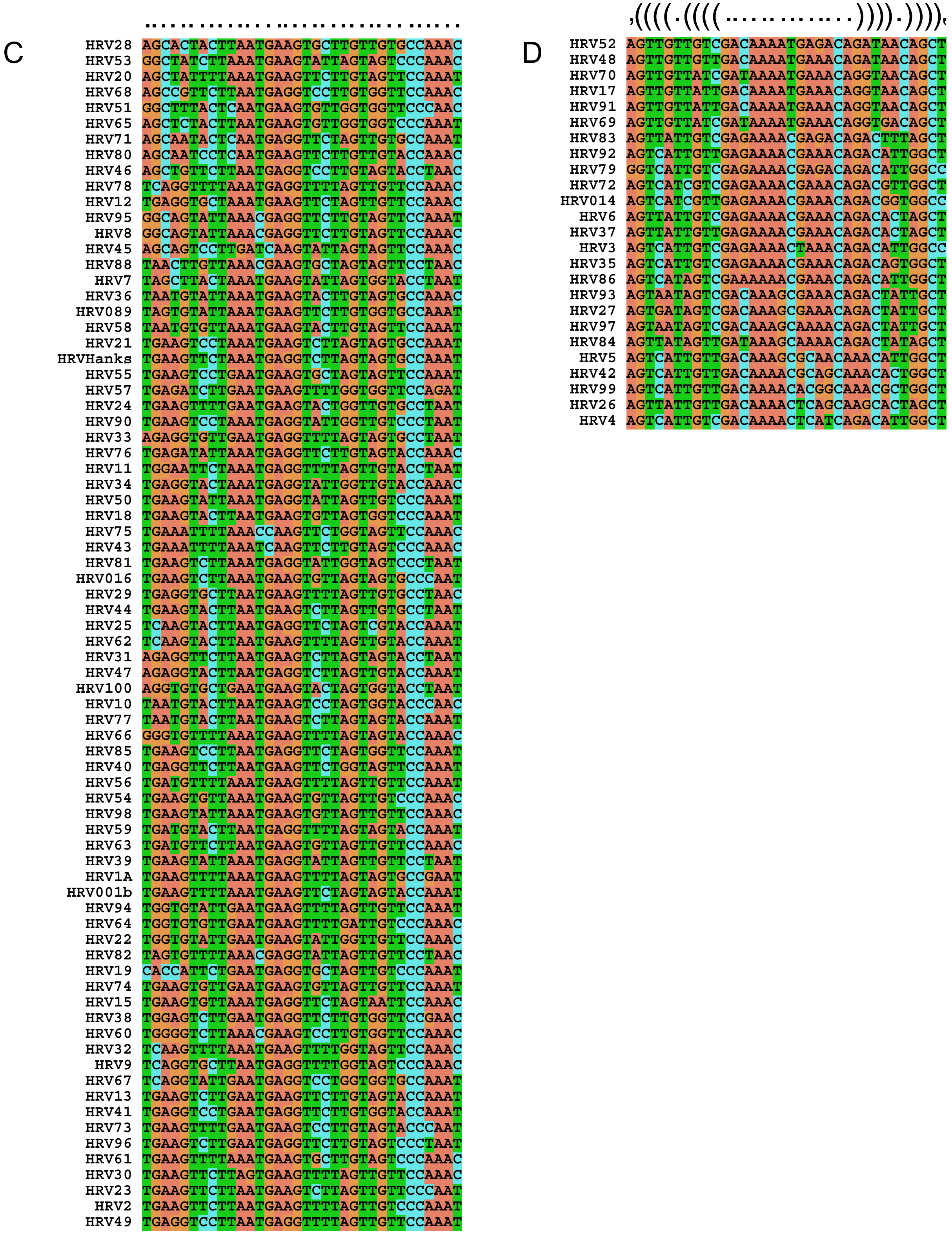
C
....
HRV28 AGCACTACTTAATGAAGTGCTTGTTGTGCCAAAC
HRV53 GGCTATCTTAAATGAAGTATTAGTAGTCCCAAAC
HRV20 AGCTATTTTAAATGAAGTTCTTGTAGTTCCAAAT
HRV68 AGCCGTTCTTAATGAGGTCCTTGTGGTTCCAAAC
HRV51 GGCTTTACTCAATGAAGTGTTGGTGGTTCCCAAC
HRV65 AGCTCTACTTAATGAAGTGTTGGTGGTCCCAAAT
HRV71 AGCAATACTCAATGAGGTTCTAGTTGTGCCAAAT
HRV80 AGCAATCCTCAATGAAGTTCTTGTTGTACCAAAC
HRV46 AGCTGTTCTTAATGAGGTCCTTGTAGTACCTAAC
HRV78 TCAGGTTTTAAATGAGGTTTTAGTTGTTCCAAAC
HRV12 TGAGGTGCTAAATGAAGTTCTAGTTGTTCCAAAC
HRV95 GGCAGTATTAAACGAGGTTCTTGTAGTTCCAAAT
HRV8 GGCAGTATTAAACGAGGTTCTTGTAGTTCCAAAC
HRV45 AGCAGTCCTTGATCAAGTATTAGTAGTTCCAAAC
HRV88 TAACTTGTTAAACGAAGTGCTAGTAGTTCCTAAC
HRV7 TAGCTTACTAAATGAAGTATTAGTGGTACCTAAT
HRV36 TAATGTATTAAATGAAGTACTTGTAGTGCCAAAC
HRV089 TAGTGTATTAAATGAAGTTCTTGTGGTGCCAAAT
HRV58 TAATGTGTTAAATGAAGTACTTGTAGTTCCAAAT
HRV21 TGAAGTCCTAAATGAAGTCTTAGTAGTGCCAAAT
HRVHanks TGAAGTTCTAAATGAGGTCTTAGTAGTGCCAAAT
HRV55 TGAAGTCCTGAATGAAGTGCTAGTAGTTCCAAAT
HRV57 TGAGATCTTGAATGAAGTTTTGGTGGTTCCAGAT
HRV24 TGAAGTTTTGAATGAAGTACTGGTTGTGCCTAAT
HRV90 TGAAGTCCTAAATGAGGTATTGGTTGTCCCTAAT
HRV33 AGAGGTGTTGAATGAGGTTTTAGTAGTGCCTAAT
HRV76 TGAGATATTAAATGAGGTTCTTGTAGTACCAAAC
HRV11 TGGAATTCTAAATGAGGTTTTAGTTGTACCTAAT
HRV34 TGAGGTACTAAATGAGGTATTGGTTGTACCAAAC
HRV50 TGAAGTATTAAATGAGGTATTAGTTGTCCCAAAT
HRV18 TGAAGTACTTAATGAAGTGTTAGTGGTCCCAAAT
HRV75 TGAAATTTTAAACCAAGTTCTGGTAGTTCCAAAC
HRV43 TGAAATTTTAAATCAAGTTCTTGTAGTCCCAAAC
HRV81 TGAAGTCTTAAATGAGGTATTGGTAGTCCCTAAT
HRV016 TGAAGTCTTAAATGAAGTGTTAGTAGTGCCCAAT
HRV29 TGAGGTGCTTAATGAAGTTTTAGTTGTGCCTAAC
HRV44 TGAAGTACTTAATGAAGTCTTAGTTGTGCCTAAT
HRV25 TCAAGTACTTAATGAGGTTCTAGTCGTACCAAAT
HRV62 TCAAGTACTTAATGAAGTTTTAGTTGTACCAAAT
HRV31 AGAGGTTCTTAATGAAGTCTTAGTAGTACCTAAT
HRV47 AGAGGTACTTAATGAGGTCTTAGTTGTACCAAAT
HRV100 AGGTGTGCTGAATGAAGTACTAGTGGTACCTAAT
HRV10 TAATGTACTTAATGAAGTCCTAGTGGTACCCAAC
HRV77 TAATGTACTTAATGAAGTCTTAGTAGTACCAAAT
HRV66 GGGTGTTTTAAATGAAGTTTTAGTAGTACCAAAC
HRV85 TGAAGTCCTTAATGAGGTTCTAGTGGTTCCAAAT
HRV40 TGAGGTTCTTAATGAAGTTCTGGTAGTTCCAAAT
HRV56 TGATGTTTTAAATGAAGTTTTAGTTGTTCCAAAT
HRV54 TGAAGTGTTAAATGAAGTGTTAGTTGTCCCAAAC
HRV98 TGAAGTATTAAATGAAGTGTTAGTTGTTCCAAAC
HRV59 TGATGTACTTAATGAGGTTTTAGTAGTACCAAAT
HRV63 TGATGTTCTTAATGAAGTGTTAGTTGTTCCAAAC
HRV39 TGAAGTATTAAATGAGGTATTAGTTGTTCCTAAT
HRV1A TGAAGTTTTAAATGAAGTTTTAGTAGTGCCGAAT
HRV001b TGAAGTTTTAAATGAAGTTCTAGTAGTACCAAAT
HRV94 TGGTGTATTGAATGAAGTTTTAGTTGTTCCAAAT
HRV64 TGGTGTGTTGAATGAAGTTTTGATTGTCCCAAAC
HRV22 TGGTGTATTGAATGAAGTATTGGTTGTTCCAAAC
HRV82 TAGTGTTTTAAACGAGGTATTAGTTGTTCCTAAC
HRV19 CACCATTCTGAATGAGGTGCTAGTTGTCCCAAAT
HRV74 TGAAGTGTTGAATGAAGTGTTAGTTGTTCCAAAT
HRV15 TGAAGTGTTAAATGAGGTTCTAGTAATTCCAAAC
HRV38 TGGAGTCTTGAATGAGGTTCTTGTGGTTCCGAAC
HRV60 TGGGGTCTTAAACGAAGTCCTTGTGGTTCCAAAC
HRV32 TCAAGTTTTAAATGAAGTTTTGGTAGTTCCAAAC
HRV9 TCAGGTGCTTAATGAGGTTTTGGTAGTCCCAAAC
HRV67 TCAGGTATTGAATGAGGTCCTGGTGGTGCCAAAT
HRV13 TGAAGTCTTGAATGAAGTTCTTGTAGTACCAAAT
HRV41 TGAGGTCCTGAATGAGGTTCTTGTGGTACCAAAT
HRV73 TGAAGTTTTGAATGAAGTCCTTGTAGTACCCAAT
HRV96 TGAAGTCTTGAATGAGGTTCTTGTAGTCCCTAAT
HRV61 TGAAGTTTTAAATGAAGTGCTTGTAGTCCCAAAC
HRV30 TGAAGTTCTTAGTGAAGTTTTAGTTGTTCCAAAC
HRV23 TGAAGTTCTTAATGAAGTCTTAGTTGTTCCCAAT
HRV2 TGAAGTTCTTAATGAAGTTTTAGTTGTCCCAAAT
HRV49 TGAGGTCCTTAATGAGGTTTTAGTTGTTCCAAAT
D
,((((.((((..............)))).)))),
HRV52 AGTTGTTGTCGACAAAATGAGACAGATAACAGCT
HRV48 AGTTGTTGTTGACAAAATGAAACAGATAACAGCT
HRV70 AGTTGTTATCGATAAAATGAAACAGGTAACAGCT
HRV17 AGTTGTTATTGACAAAATGAAACAGGTAACAGCT
HRV91 AGTTGTTATTGACAAAATGAAACAGGTAACAGCT
HRV69 AGTTGTTATCGATAAAATGAAACAGGTGACAGCT
HRV83 AGTTATTGTCGAGAAAACGAGACAGACTTTAGCT
HRV92 AGTCATTGTTGAGAAAACGAAACAGACATTGGCT
HRV79 GGTCATTGTCGAGAAAACGAGACAGACATTGGCC
HRV72 AGTCATCGTCGAGAAAACGAAACAGACGTTGGCT
HRV014 AGTCATCGTTGAGAAAACGAAACAGACGGTGGCC
HRV6 AGTTATTGTCGAGAAAACGAAACAGACACTAGCT
HRV37 AGTTATTGTTGAGAAAACGAAACAGACACTAGCT
HRV3 AGTCATTGTCGAGAAAACTAAACAGACATTGGCC
HRV35 AGTCATTGTCGAGAAAACGAAACAGACAGTGGCT
HRV86 AGTCATAGTCGAAAAAACGAAACAGACATTGGCT
HRV93 AGTAATAGTCGACAAAGCGAAACAGACTATTGCT
HRV27 AGTGATAGTCGACAAAGCGAAACAGACTATTGCT
HRV97 AGTAATAGTCGACAAAGCAAAACAGACTATTGCT
HRV84 AGTTATAGTTGATAAAGCAAAACAGACTATAGCT
HRV5 AGTCATTGTTGACAAAGCGCAACAAACATTGGCT
HRV42 AGTCATTGTTGACAAAACGCAGCAAACACTGGCT
HRV99 AGTCATTGTTGACAAAACACGGCAAACGCTGGCT
HRV26 AGTTATTGTTGACAAAACTCAGCAAGCACTAGCT
HRV4 AGTCATTGTCGACAAAACTCATCAGACATTGGCT
