# Supplementary material for: Genome-wide diversity and selective pressure in the human rhinovirus
Source: Virol J. 2007 May 3;4:40. doi: 10.1186/1743-422X-4-40 (PMC1892812; doi:10.1186/1743-422X-4-40)
Supplement: Additional File 5 — Supplemental Tables 4-6 and Supplemental Figure 9. [file 1743-422X-4-40-S5.pdf]

Table S4 - HRV serotypes used in this study for whole genome sequence analysis.

Table S5 - Genome assembly statistics.

Table S6 - PAML gene-specific codon model parameters.

Figure S9 - Location of most diversifying residues in HRV capsid genes assuming a homogeneous synonymous substitution rate.

Top 5% diversifying residues within the HRV capsid genes mapped onto the viral pentamer structure computed by: A. codonml (Yang, 1997), and B. REL (non-synonymous), or assuming a variable synonymous substitution rate, C. REL (Dual), (Pond and Frost, 2005). (PDF)

TABLE S4. HRV serotypes used for whole genome sequence analysis

HRV serotype	HRV subgroup	Source[†]	Identifier*	Isolation Date**
HRV001b	A	CaDHS	TC-72697	4/23/1973
HRV001b	A	NCBI	D00239	NA
HRV002	A	NCBI	X02316	NA
HRV003	B	CaDHS	TC-64701	10/24/1967
HRV004	B	CaDHS	NA	10/26/1967
HRV006	B	CaDHS	TC-65006	10/31/1967
HRV007	A	CaDHS	TC-65007	11/3/1967
HRV010	A	CaDHS	TC-71602	4/26/1971
HRV014	B	NCBI	K02121	NA
HRV015	A	CaDHS	TC-66919	5/10/1968
HRV016	A	NCBI	L24917	NA
HRV023	A	CaDHS	TC-65847	10/27/1967
HRV028	A	CaDHS	TC-65852	10/30/1967
HRV030	A	CaDHS	TC-67821	5/7/1969
HRV034	A	CaDHS	TC-65856	11/6/1967
HRV035	B	CaDHS	TC-73280	5/9/1972
HRV036	A	CaDHS	TC-74313	10/20/1973
HRV038	A	CaDHS	TC-72617	9/22/1972
HRV039	A	NCBI	AY651783	NA
HRV041	A	CaDHS	TC-66066	6/7/1967
HRV044	A	CaDHS	TC-72548	5/12/1972
HRV046	A	CaDHS	TC-75908	3/3/1975
HRV048	B	CaDHS	TC-70326	11/16/1970
HRV049	A	CaDHS	TC-66958	7/8/1968
HRV053	A	CaDHS	TC-67618	7/26/1968
HRV055	A	CaDHS	TC-64522	9/7/1967
HRV059	A	CaDHS	TC-70403	12/7/1970
HRV070	B	CaDHS	TC-72589	7/25/1972
HRV073	A	CaDHS	TC-73172	3/31/1972
HRV074	A	CaDHS	TC-70882	11/8/1971
HRV075	A	CaDHS	TC-70308	10/26/1970
HRV076	A	CaDHS	TC-70326	11/13/1970
HRV087	---	CaDHS	TC-70827	10/20/1971
HRV087/HEV68	---	NCBI	AY426531	NA
HRV088	A	CaDHS	TC-70782	8/27/1971
HRV089	A	NCBI	A10937	NA
HRVsc	A	CaDHS	TC-4669	11/24/2003

[†]CaDHS=California Department of Health Services Viral and Rickettsial Disease Laboratory; NCBI=National Center for Biotechnology Information Database; * CaDHS vial number or Genbank accession number; **NA=not available.

TABLE S5. Genome Assembly Statistics

			Base Quality			Read Depth		
Serotype	Length	#Reads	Min	Ave	Max	Min	Ave	Max
HRV003	7208	368	23	88.2	90	1	31.2	83
HRV004	7212	503	26	89.1	99	1	27.2	83
HRV006	7215	198	12	79.8	99	1	13.6	60
HRV007	7148	407	19	84.3	90	2	32.9	162
HRV010	7137	210	24	87.9	90	2	17.1	44
HRV015	7134	223	30	84.5	90	1	16.5	79
HRV023	7025	301	10	86.3	90	1	23.2	125
HRV028	7148	333	11	86.6	99	2	28.7	94
HRV030	7015	319	12	82.9	90	1	23.3	138
HRV034	7119	287	20	82.5	90	2	19.3	45
HRV035	7225	221	13	85.5	90	1	15.6	57
HRV036	7140	345	36	87.3	98	2	22.9	97
HRV038	7136	313	44	88.4	90	1	21.8	74
HRV041	7145	332	11	89.1	90	2	23.8	87
HRV044	7123	296	9	88.3	99	1	22.5	75
HRV046	7149	320	11	80.6	90	1	24.2	169
HRV048	7214	206	15	88.6	90	2	15.8	62
HRV049	7106	252	59	89.3	90	3	21.0	66
HRV053	7143	288	15	89.2	99	1	20.5	64
HRV055	6957	211	56	89.8	90	1	18.2	48
HRV059	7135	354	11	87.4	90	1	27.2	90
HRV070	7223	606	12	88.3	90	1	46.0	132
HRV073	7140	297	26	89.3	90	1	21.7	62
HRV074	7120	325	14	88.9	90	1	28.4	88
HRV075	7100	257	73	89.8	99	2	21.6	42
HRV076	7129	317	20	85.1	99	1	21.4	194
HRV087	7320	146	15	77.8	90	1	9.4	32
HRV088	7143	321	17	82.8	90	2	22.5	130
HRVsc	6967	275	12	88.5	99	1	16.8	47
**Average**	**7150**	**304**	**22**	**86.4**	**93**	**1**	**22**	**87**

TABLE S6. PAML gene-specific codon model parameters

		M1	M2			M7	M8			2 x ΔlnL	
**Gene**	**S***	**lnL**	**lnL**	**p(ω2)**	**ω2**	**lnL**	**lnL**	**p(ω1)**	**ω1**	**M2-M1**	**M8-M7**
VP4	A	-2014.06	-2014.06	0.00	7.47	-1971.20	-1971.20	0.00	2.62	0.00	0.00
VP4	B	-766.72	-766.73	0.00	1.00	-764.08	-764.08	0.00	2.14	0.00	0.00
VP2	A	-10922.94	-10922.94	0.01	1.00	-10635.94	-10635.94	0.00	4.47	0.00	0.00
VP2	B	-3681.33	-3681.33	0.00	36.30	-3621.05	-3621.05	0.00	8.06	0.00	0.00
VP3	A	-10956.49	-10956.49	0.00	25.88	-10644.99	-10644.99	0.00	3.00	0.00	0.00
VP3	B	-3312.49	-3312.49	0.00	25.41	-3268.67	-3268.67	0.00	26.76	0.00	0.00
VP1	A	-13303.40	-13303.40	0.00	36.24	-12806.15	-12803.09	0.01	1.00	0.00	6.12
VP1	B	-4230.79	-4230.79	0.00	1.00	-4180.79	-4177.18	0.02	53.36	0.00	7.22
P2A	A	-4814.03	-4814.03	0.00	11.53	-4733.04	-4733.04	0.00	1.00	0.00	0.00
P2A	B	-1986.47	-1986.47	0.00	27.01	-1955.31	-1955.31	0.00	1.00	0.00	0.00
P2B	A	-3460.73	-3460.73	0.00	1.00	-3402.27	-3402.27	0.00	2.99	0.00	0.00
P2B	B	-1248.17	-1248.17	0.00	1.00	-1237.68	-1237.68	0.00	1.00	0.00	0.00
P2C	A	-12247.46	-12247.46	0.00	48.54	-11906.17	-11906.17	0.00	2.97	0.00	0.00
P2C	B	-4149.52	-4149.52	0.00	35.74	-4104.74	-4104.74	0.00	52.56	0.00	0.00
P3A	A	-3011.64	-3011.64	0.00	6.85	-2941.04	-2941.04	0.00	2.85	0.00	0.00
P3A	B	-1022.07	-1022.07	0.00	5.51	-1009.81	-1009.81	0.00	10.24	0.00	0.00
Vpg	A	-763.87	-763.87	0.00	3.21	-748.90	-748.90	0.00	3.20	0.00	0.00
Vpg	B	-325.54	-325.54	0.03	1.00	-322.27	-322.27	0.00	2.18	0.00	0.00
P3C	A	-7093.92	-7093.92	0.00	30.69	-6871.20	-6871.20	0.00	2.39	0.00	0.00
P3C	B	-2503.73	-2503.73	0.03	1.00	-2465.49	-2465.49	0.00	6.75	0.00	0.00
Pol	A	-18753.77	-18753.77	0.00	33.41	-18093.48	-18093.48	0.00	5.91	0.00	0.00
Pol	B	-6293.96	-6293.96	0.00	1.00	-6196.52	-6194.36	0.01	1.82	0.00	4.32

*S=serotype of HRV data set

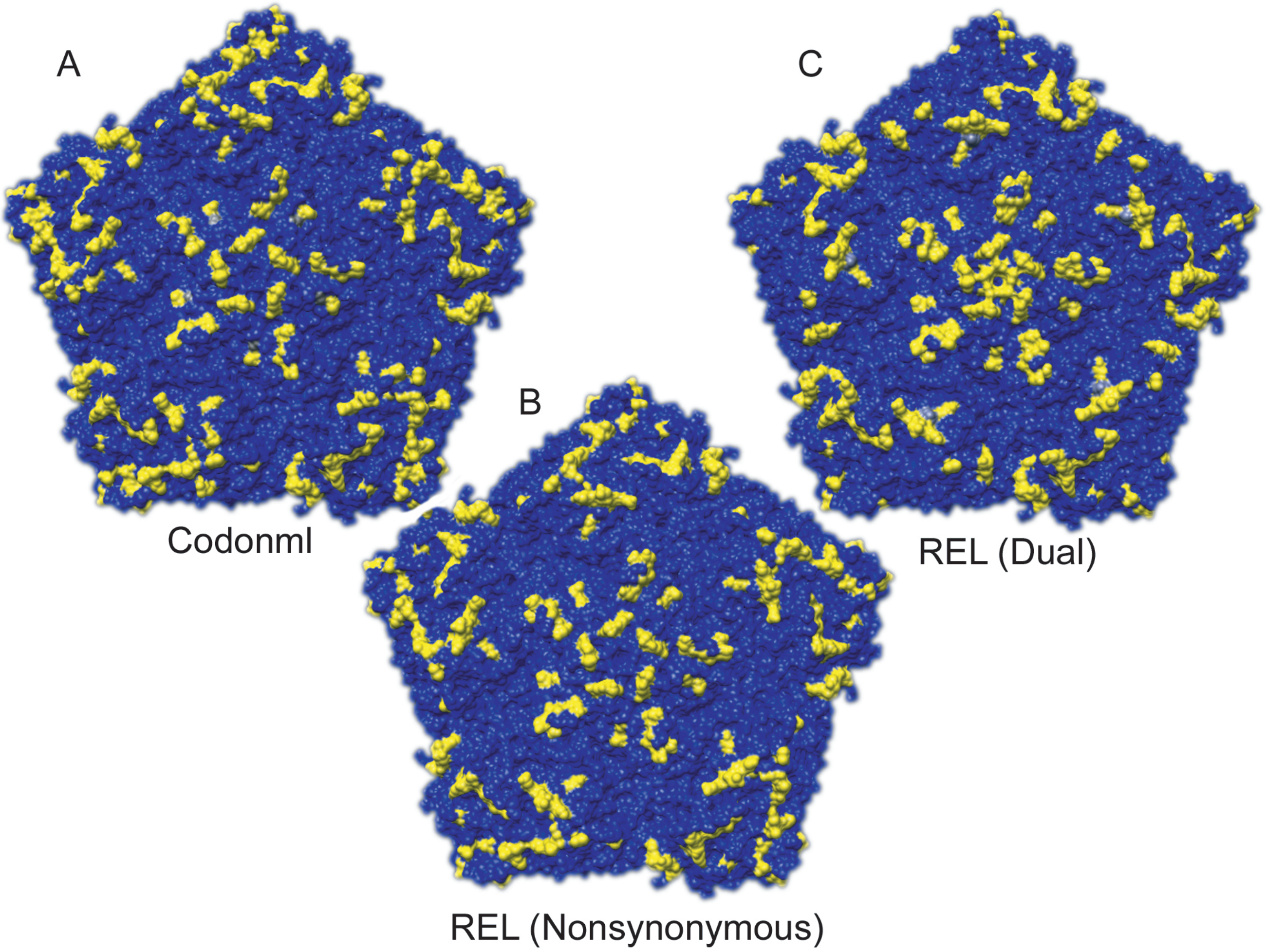

Fig. S9
