# Supplementary material for: Genome-wide diversity and selective pressure in the human rhinovirus
Source: Virol J. 2007 May 3;4:40. doi: 10.1186/1743-422X-4-40 (PMC1892812; doi:10.1186/1743-422X-4-40)
Supplement: Additional File 6 — Supplemental Methods. [file 1743-422X-4-40-S6.pdf]

Supplemental Methods:

Analysis of RNA secondary structures. The locations of the HRV 5’NCR cloverleaf element, the IRES, 3’NCR stem loop structure, and CRE have been previously described {Witwer, 2001 #29}. CLUSTALW alignments of each of these regions were generated and consensus secondary structures based on phylogenetic conservation, covariation analysis, and thermodynamic folding parameters for each element were generated via alifold {Hofacker, 2004 #30}.

Predicted MHC1 ligand analysis. All peptides of length 8 to 10 within the fully-sequenced HRV genomes were scored against a matrix of known MHC1 binding motifs {Rammensee, 1999 #76}. Resulting scores were plotted along the genome and along each gene for visual comparison to the dN/dS data, and Pearson correlation values with the dN/dS data were computed.

Electrostatic surface potential analysis. The electrostatic surface potential for each region of the capsid, 3C, and 3D proteins was calculated using the Adaptive Poisson-Boltzmann solver implemented in the APBS software package {Baker, 2001 #130} and visualized using Chimera.

Genome-wide amino acid covariation analysis. Covariation scores between all pairs of amino acids in the HRV genome were computed using the OMES method (Observed Minus Expected, Squared; {Fodor, 2004 #131; Kass, 2002 #135}). The 100 highest scoring pairs were mapped onto corresponding protein structures where available to

assess the significance of the scores. The highest scoring pairs associated with each diversifying residue in the 3C protease and 3D polymerase proteins were also mapped on to available protein structures to assess the likelihood of covariation involving these residues.
